# ATRX modulates the escape from a telomere crisis

**DOI:** 10.1371/journal.pgen.1010485

**Published:** 2022-11-09

**Authors:** Helene E. B. Geiller, Adam Harvey, Rhiannon E. Jones, Julia W. Grimstead, Kez Cleal, Eric A. Hendrickson, Duncan M. Baird

**Affiliations:** 1 Division of Cancer and Genetics, School of Medicine, Cardiff University, Heath Park, Cardiff, United Kingdom; 2 Department of Biochemistry, Molecular Biology, and Biophysics, University of Minnesota Medical School, Minneapolis, Minnesota, United States of America; Children’s Medical Research Institute, AUSTRALIA

## Abstract

Telomerase activity is the principal telomere maintenance mechanism in human cancers, however 15% of cancers utilise a recombination-based mechanism referred to as alternative lengthening of telomeres (ALT) that leads to long and heterogenous telomere length distributions. Loss-of-function mutations in the Alpha Thalassemia/Mental Retardation Syndrome X-Linked (ATRX) gene are frequently found in ALT cancers. Here, we demonstrate that the loss of ATRX, coupled with telomere dysfunction during crisis, is sufficient to initiate activation of the ALT pathway and that it confers replicative immortality in human fibroblasts. Additionally, loss of ATRX combined with a telomere-driven crisis in HCT116 epithelial cancer cells led to the initiation of an ALT-like pathway. In these cells, a rapid and precise telomeric elongation and the induction of C-circles was observed; however, this process was transient and the telomeres ultimately continued to erode such that the cells either died or the escape from crisis was associated with telomerase activation. In both of these instances, telomere sequencing revealed that all alleles, irrespective of whether they were elongated, were enriched in variant repeat types, that appeared to be cell-line specific. Thus, our data show that the loss of ATRX combined with telomere dysfunction during crisis induces the ALT pathway in fibroblasts and enables a transient activation of ALT in epithelial cells.

## Author summary

Telomeres are nucleoprotein structures that cap the ends of linear chromosomes, they are essential for chromosomal stability, but gradually shorten with ongoing cell division. The loss of telomeric DNA ultimately leads to the loss of the end capping function, the induction of widespread genomic instability and cellular crisis. This period of crisis leads to the acquisition of telomere maintenance mechanisms (TMM) that are required to confer replicative immortality in cancer cells. The majority of tumour types use telomerase as their TMM, but a significant subset of cancers utilise the alternative lengthening of telomeres (ALT) pathway. Here we show that, in the absence of the Alpha Thalassemia/Mental Retardation Syndrome X-Linked (ATRX) gene, fibroblast cells, that very rarely escape crisis, can efficiently escape crisis having induced the ALT pathway. In contrast, epithelial cells that escape crisis having activated telomerase, are restricted by the absence of ATRX, but can induce a transient ALT-like activity in a small proportion of cells. Our data point to an important role of ATRX in conferring telomere stability and restricting clonal evolution during a telomere-driven crisis in cells of a mesenchymal origin.

## Introduction

Telomeres are repetitive DNA:protein elements that protect the ends of linear chromosomes and prevent their recognition as double-stranded DNA breaks [1]. As a consequence of the “end-replication problem”, telomeres shorten with every successive cell cycle and such shortening ultimately limits the proliferative capacity of cells, by eliciting a TP53-dependent G_1_/S cell cycle arrest that acts as a stringent tumour suppressive mechanism [2]. In the absence of a functional cell-cycle arrest, continued cell division and telomere erosion ultimately result in a period of telomere dysfunction, referred to as “crisis” [3]. Telomere dysfunction during crisis leads to large-scale genomic rearrangements from which cells can escape by activating a telomere maintenance mechanism (TMM) that rescues cell viability but drives clonal evolution and tumour progression [4, 5]. The majority of malignancies, as well as stem cells, germ cells and single-celled organisms, almost exclusively utilise the enzyme complex telomerase as their primary TMM [6, 7]. However, 15% of malignancies, predominantly those of mesenchymal origin [8], do not express telomerase, but instead maintain their telomeres via the Alternative Lengthening of Telomeres (ALT) mechanism [9,10].

ALT was originally characterised by extreme telomere length heterogeneity and an absence of telomerase activity [11]. Subsequently, ALT-associated promyelocytic leukaemia (PML) nuclear bodies (APBs) were identified that contain telomeric repeat DNA and the telomere binding proteins telomere repeat factors 1 and 2 (TRF1 and TRF2), associated with the PML protein [12]. APBs also contain components of the homologous recombination machinery [13,14] and associate with chromosome ends [15] implicating these factors and structures in the underlying mechanisms of ALT. Indeed, specifically-tagged telomeres were shown to be copied onto different chromosome ends in ALT cells [16], supporting a role for recombination in ALT. Moreover, ALT can be suppressed by the abrogation of the key recombination complex, Mre11:Rad50:Nbs1 (MRN) [17]. Finally, certain characteristics of the ALT phenotype are consistent with recombination-mediated mechanisms for telomere elongation including Break Induced Replication (BIR), rolling circle amplification and unequal sister chromatid exchange [18,19].

More recent work has uncovered a strong correlation between malignancies exhibiting the ALT phenotype and mutations in the Alpha Thalassemia/Mental Retardation Syndrome X-Linked (ATRX) gene [20,21]. ATRX is a chaperone for histone H3.3, and along with Death Domain Associated Protein (DAXX), is responsible for H3.3’s replication-independent incorporation into the genome [22,23]. Specifically, H3.3 deposition into telomeric regions seems to be altered in ALT cells and, correspondingly, telomeric chromatin dynamics are altered [23–26]. How ATRX might contribute to the onset of ALT and why ALT appears to occur primarily in cells of mesenchymal origin is unclear [27]. The loss of ATRX, however, co-segregates with the ALT phenotype in cell fusion experiments [28]. Additionally, the frequency of ALT immortalisation events increases with the shRNA knock-down of ATRX in human fibroblasts [29,30]. Lastly, ectopic re-expression of ATRX in ALT cells diminishes their ALT activity [30,31].

In order to gain further insights into the mechanism of ALT, we have examined the earliest stages of telomeric elongation in the absence of ATRX during a telomere crisis in primary human fibroblasts and epithelial cancer cells. The absence of ATRX enabled the successful escape from crisis in fibroblasts, of mesenchymal origin, by the initiation of the ALT mechanism, whilst it compromised the ability of non-mesenchymal epithelial cells to escape crisis. However, epithelial cells in crisis exhibited manifestations of the ALT phenotype including the presence of C-circles, which were observed irrespective of whether they were capable of escaping crisis. Intriguingly, a sub-set of cells displayed specific telomeric elongation events, whereby the shortest telomeric alleles were subjected to elongation to chromosome-specific telomere lengths. Despite the presence of C-circles, this “ALT-like” telomeric elongation was not maintained, with elongated alleles ultimately being subjected to end-replication losses. Thus, the loss of ATRX combined with telomere dysfunction during crisis induces an ALT pathway, which confers replicative immortality in fibroblasts, but is transient in HCT116 epithelial cells. We also show an increase in non-canonical variant repeats in ALT telomeres compared to non-ALT controls.

## Materials and methods

### Cell culture

HCA2^HPVE6E7^ skin fibroblasts were provided by James Smith, Houston, US. They were cultured in DMEM supplemented with 10% FCS, 2 mM glutamine, 0.1 mg/ml streptomycin, 100 U/ml penicillin and 4 μg/ml G418. Cells were cultured in T75 flasks and passaged every 7 days.

MRC5^HPVE6E7^ lung fibroblasts were obtained from the Coriell Institute cell repository [32]. They were cultured in MEM supplemented with 10% FCS, 2 mM glutamine, 0.1 mg/ml streptomycin, 100 U/ml penicillin, 4 μg/ml G418, 1x NEAA, 3 mM NaOH and 0.2% NaHCO_3_. Cells were cultured in T75 flasks and passaged every 7 days.

HCT116^ATRX-/0^ cells [30] were cultured in McCoy’s 5A medium supplemented with 10% FCS, 2 mM glutamine, 0.1 mg/ml streptomycin, 100 U/ml penicillin and 4 μg/ml G418. Cells were cultured in T25 flasks and passaged every 7 days.

At each passage, samples were collected for cell counts to track population doublings (PDs), and for DNA and protein extractions.

### CRISPR/Cas9 gene editing

The ATRX gene was targeted by CRISPR/Cas9 gene editing in HCA2^HPVE6E7^ and MRC5^HPVE6E7^ cells [30]. The ATRX target sequence, 5′-GTTTCTGTCGGTCGCCTCAA-3′, was used as the guide RNA to target exon 9 of the *ATRX* gene and ligated into a pSpCas9(BB)-2A-GFP plasmid (Addgene #48138) using Bbs1 restriction enzyme cut sites. 24 hr after nucleofection, cells were sorted according to GFP intensity by flow cytometry and plated for single-cell cloning.

### Lentiviral transfection

Retroviral transfections were used to transfect HCT116^ATRX-/0^ cells with a dominant negative-hTERT (DN-hTERT) cassette to abrogate telomerase activity. Recombinant retroviruses containing a pBABE-puro vector (Addgene) encoding a DN-hTERT and puromycin selection genes were grown using ΨCRIP cells, gifted by Richard Mulligan (Whitehead Institute, Cambridge) [33]. Cells that had successfully integrated the vector were selected in puromycin 72 hr after addition of the retrovirus to the cells (2.5 μg/ml; Calbiochem) and medium containing puromycin was subsequently used for the culturing of these cells. Expanded cells were ultimately plated for single-cell cloning.

### DNA extraction

DNA was extracted using standard RNase A, Proteinase K and phenol:chloroform methods, [34] ethanol precipitated, washed in 70% ethanol, air-dried, resuspended in 50 μl of 10 mM Tris-HCl and quantified in triplicate using Hoechst fluorometry (Bio-Rad). A working stock of 20 μl at a concentration of 10 ng/μl was made for each sample.

### STELA

Single telomere length analysis (STELA) was undertaken as described [35]. Two primers were used to amplify specific telomeres in the HCA2 model: XpYpE2 and 17p6. Three primers were used to amplify specific telomeres in the MRC5 model: XpYpE2, XpYpAT and XpYpGC. Six primers were used to amplify specific telomeres in the HCT116 model: XpYpC, 5p5, 7qK1, 8q2, 9p2 and 17pseq1rev. The PCR conditions were as follows: 94°C for 20 s; 59°C (5p5, 17pseq1rev and 17p6), 61°C (9p2), 65°C (7qK1, 8q2, XpYpC, XpYpE2, XpYpAT and XpYpGC) for 30 s; 68°C for 8 min for 22 cycles. DNA fragments were resolved with 0.5% TAE agarose gel electrophoresis and detected by Southern hybridisation at 55°C overnight with a ^32^P radiolabelled telomere repeat (TTAGGG)_n_-containing probe together with probes to detect the 1 kb and 2.5 kb markers. The blots were washed four times at 55°C with 0.1% SDS and 0.1X SSC, dried, exposed to a phosphor screen and scanned with a Typhoon FLA 9500 phosphoimager (GE Healthcare) and analysed using ImageQuant TL (GE Healthcare).

### Fusion PCR

Fusion PCR was undertaken as described [36]. Three primers were used to amplify inter- and intra- allelic fusion events in the HCA2 model: 17p6, 21q1 and XpYpM. The PCR conditions were as follows: 94°C for 20 s; 62°C for 30 s; and 68°C for 8 min and repeated for 25 cycles. DNA fragments were resolved with 0.5% TAE agarose gel electrophoresis and detected by Southern hybridisation at 55°C overnight with a ^32^P radiolabelled chromosome-specific (17p, 21q and XpYp) probe together with probes to detect the 1 kb and 2.5 kb markers. The blots were then washed four times at 55°C with 0.1% SDS and 0.1X SSC, dried, exposed to a phosphor screen and scanned with a Typhoon FLA 9500 phosphoimager (GE Healthcare) and analysed using ImageQuant TL (GE Healthcare).

### C-circle assay

For detecting C-circles, 20 ng of genomic DNA was incubated with 7.5 U of φ29 DNA polymerase, 1 mM dATP, dGTP and dTTP, 0.2 mg/mL BSA, 0.1% Tween 20 and 1X φ29 buffer for 8 hr at 30°C as described [37]. The samples were then denatured and slot blotted onto a positively-charged hybridisation membrane (Hybond XL; GE Healthcare). The membranes were pre-hybridised in Church’s buffer (1% (w/v) BSA; 1 mM EDTA; 0.5 M phosphate buffer and 7% (w/v) SDS) and hybridised overnight at 55°C with a ^32^P radiolabelled telomere repeat (TTAGGG)_n_ probe. The membranes were then washed, dried, exposed, scanned and quantitated as described above.

### TRAP assay

Telomerase activity in the HCT116 cell line was quantified using the TRAPeze XL telomerase detection kit according to the manufacturer’s instructions (Millipore). Protein was extracted using CHAPS lysis buffer. Protein concentrations were determined by spectrophotometry and a working stock of 30 μl at 100 ng/μl was prepared for each sample to be amplified by PCR. The excitation and emission state wavelengths for fluorescein (485 nm and 535 nm, respectively) and sulforhodamine (585 nm and 620 nm, respectively) were measured on a Cytation3 plate reader (BioTek). All subsequent calculations were done in GraphPad Prism 5 and telomerase activity was expressed as Total Product Generated (TPG).

Telomerase activity in the HCA2 and MRC5 cell lines was quantified as described [38]. Following protein extraction and quantification as described above, 500 ng of protein was incubated with 1X TRAP buffer (2X stock: 40 mM Tris HCl pH 8; 3 mM MgCl_2_; 126 mM KCl; 0.01% Tween 20; 2 mM EGTA; 0.2 mg/ml BSA; 0.1 mM dNTPs); 0.36 μM TS primer; 1 μl primer mix (stock 0.10 μM ACX primer; 0.19 μM NT primer; and 0.0025 pM TSNT internal control primer: 5′-AATCCGTCGAGCAGAGTTAAAAGGCCGAGAAGCGAT-3′); 0.4X Titanium Taq polymerase (Clontech); and ddH_2_O to make up 50 μl. Reactions were processed using a Tetrad thermal cycler (Bio-Rad) at the following conditions: 25°C for 40 min; 95°C for 5 min for 1 cycle; 95°C for 30 s; 52°C for 30 s; and 72°C for 45 s for 29 cycles followed by 72°C for 10 min for 1 cycle. 10 μl of 6X Ficoll gel loading solution (5% bromophenol blue; 5% xylene; and 15% Ficoll) were added to each reaction. TRAP PCR products were resolved on acrylamide gels (12.5% acrylamide 19:1; 0.06% APS; 0.125% TEMED; and 0.6X Tris-borate-EDTA (TBE)). Gels were electrophoresed in 1X TBE (400 ml of dH_2_O and 100 ml of 5X TBE stock: 0.45 M Tris; 0.45 M boric acid; and 10 mM EDTA pH 8) for 1 to 2 hrs at 100 V. To visualise telomerase activity, gels were incubated in 1:10,000 SYBR-gold for 10 min on a rocker at room temperature. Gels were then scanned using the typhoon FLA 9500 scanner using the SYBR-Gold filter (473 nm laser wavelength; 200 micron pixel size).

### Oligonucleotides

Telorette2; 5′-TGCTCCGTGCATCTGGCATCTAACCCT-3′

Teltail; 5′-TGCTCCGTGCATCTGGCAT-3′

5p5; 5′-GGAGCAGCATTCTCTTCACCACAG-3′

7qK1; 5′-GGGCACTGCCTCGCTTTGA-3′

9p2; 5′-CACATTCCTCATGTGCTTACG-3′

17pseq1rev; 5′-GAATCCACGGATTGCTTTGTGTAC-3′

17p6; 5′-GGCTGAACTATAGCCTCTGC-3′

21q1; 5′-CTTGGTGTCGAGAGAGGTAG-3′

XpYpC; 5′-CAGGGACCGGGACAAATAGAC-3′

XpYpE2; 5′-TTGTCTCAGGGTCCTAGTG-3′

XpYp-427G/415C; 5′-GGTTATCGACCAGGTGCTCC-3′

XpYp-427A/415T; 5′-GGTTATCAACCAGGTGCTCT-3′

XpYpM; 5′-ACCAGGTTTTCCAGTGTGTT-3′

### Whole genome sequencing

27 HCT116 DNA samples (a combination of ALT-positive and telomerase-positive clones) were whole genome sequenced using the BGISEQ-500 platform, providing paired end (2X 100 bps) sequencing with a 15X coverage. A minimum of 20 μl at a concentration of 1 μg per sample was assayed. The QC, library preparation, sequencing and data filtering were carried out by BGI. Sequence mapping and analysis were carried out as described [39].

### PacBio SMRT sequencing analysis—generation of sequencing samples

To generate PCR amplicons to be sequenced, specific chromosome ends were amplified using STELA. A minimum of 500 ng of DNA were required for PacBio sequencing and therefore 1,600 reactions were generated for each sample. To amplify multiple telomeres in one reaction, multiple primers were added to the master mix (XpYpC, 7qK1 and 17pseq1rev for HCT116 samples; XpYpE2 and 17p6 for HCA2 samples; and XpYpE2 and 17pseq1rev for the U2OS sample) adjusting the volume of ddH_2_O accordingly to limit the amount of input genomic DNA for optimal sequencing. The reactions were processed using a Tetrad thermal cycler (Bio-Rad) at the following conditions: 94°C for 20 s; 63°C for 30 s; and 68°C for 8 min for 24 cycles. Following PCR amplification, sample reactions were pooled together and concentrated using an ISS110 Speedvac system (Thermo Fisher Scientific) to evaporate excess water using a vacuum. All samples were then purified using AMPure XP beads (Beckman Coulter) according to the manufacturer’s manual. Samples were then processed for PacBio library preparations.

### PacBio SMRT sequencing analysis—data processing

The raw reads were filtered to retain only reads with a sub-telomere primer at one end and a telorette primer at the other end. This was accomplished by aligning the reads using Edlib. Then, all sequences were labelled using a Hidden Markov Model (HMM) to highlight and dissociate telomere repeat arrays, sub-telomere sequences and insertions. Sequences were broken down into 6 bp kmers and given scores upon comparison to the canonical telomere repeat TTAGGG allowing an edit distance of 2 bps. The scores were as follows: 0 for background sequence (sub-telomere and insertions); 1 for forward strand telomere repeats (CCCTAA); and 2 for reverse strand telomere repeats (TTAGGG). Therefore, variant repeats with a maximum of 2 bp substitutions compared to TTAGGG were classed as telomere sequence. To clean the data further, subsequent filtering steps were added to the pipeline, which were aimed at removing sequencing and PCR artefacts generated during the process. By this means, the following classes of reads were removed from the analysis: unexpected non-sub-telomeric sequences amplified by low homology with primers; STELA products that appeared to have undergone primer swapping; products that did not have a detectable sub-telomere sequence; and concatemers of STELA products. All of the retained sequences were then compiled into an Excel spreadsheet for manual curation and analysis. The spreadsheet included the sub-telomere length, trimmed telomere length as well as the extension lengths and sequences amongst other features. More detail of the methods used can be found in S1 Methods.

## Results

### The loss of ATRX induces crisis survival through ALT in primary human fibroblasts

**Fig 1:**
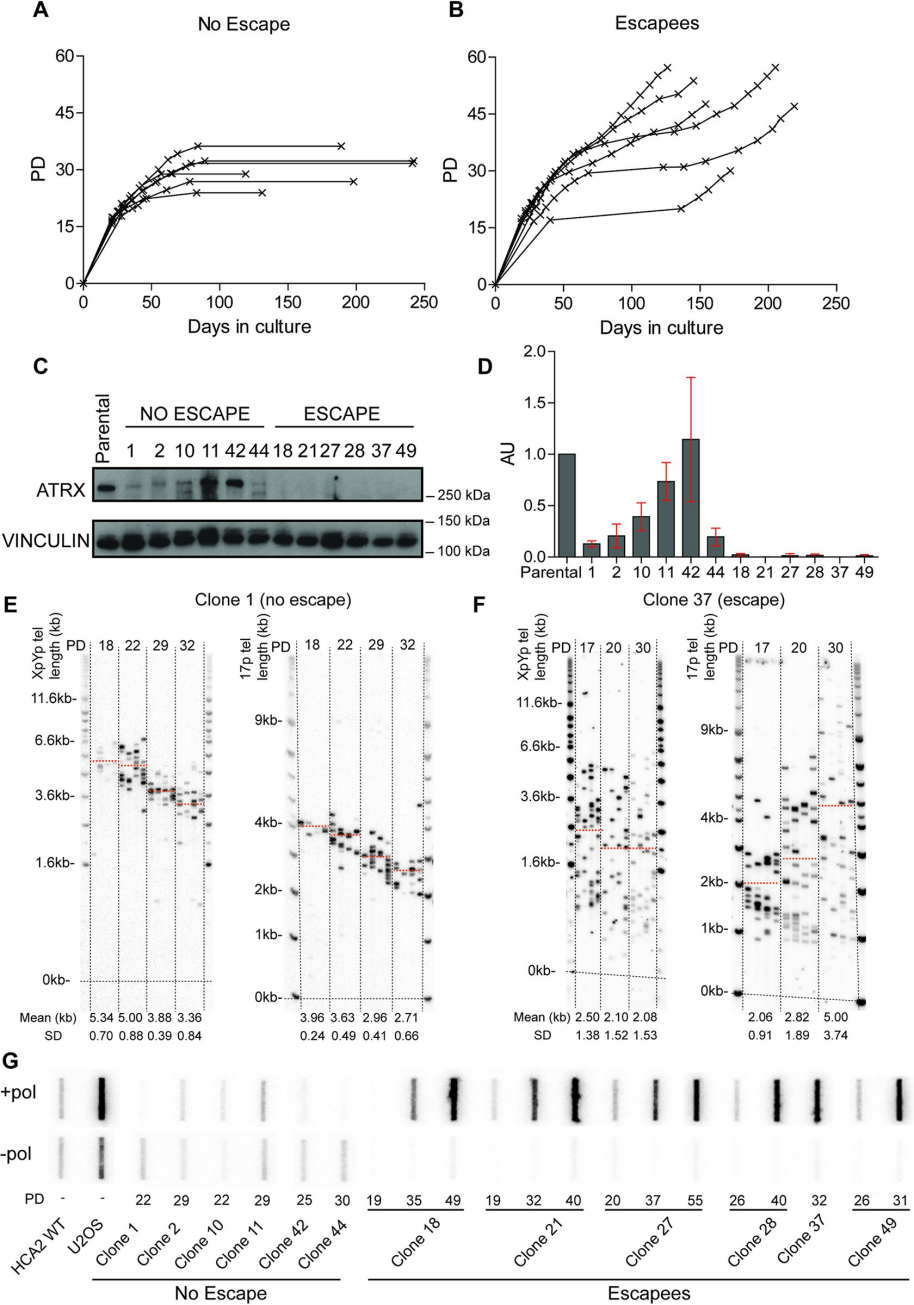
ALT upregulation and maintenance in the absence of ATRX in primary fibroblasts undergoing a telomere-driven “crisis”. Growth curves displaying PDs graphed against days in culture for (A) HCA2^HPVE6E7^ clones (n = 6) that failed to escape crisis and (B) HCA2^HPVE6E7^ clones (n = 6) that successfully escaped crisis. (C) Western blots displaying ATRX protein expression in “no escape” and “escape” clones with vinculin expression used as a loading control. (D) Quantification of the ATRX protein expression using the ATRX:vinculin ratio normalised to the parental cell line expressed in arbitrary units (AU) with the standard deviation (SD) used as error bars and the clone number displayed across the bottom. STELA profiles at the XpYp and 17p chromosome ends for (E) clone 1 that failed to escape crisis and (F) clone 37 that successfully escaped crisis; with the PD indicated across the top and the mean telomere length in kb, also represented as orange dotted lines on the blot, and SD across the bottom. (G) C-circle assay slot blots with (+ pol) and without (- pol) polymerase samples with the PD and clone number stated across the bottom.

We examined telomere dynamics during crisis in the absence of ATRX and queried whether this impacts the ability of mesenchymal cells to achieve replicative immortality. Telomere crisis was initiated in two primary fibroblast cell lines, HCA2 skin fibroblasts and MRC5 lung fibroblasts, following infection with amphotropic retroviral vectors encoding HPV16 E6 and E7 to abrogate TP53 and retinoblastoma (Rb). Approximately 20 to 30 population doublings (PDs) prior to the onset of crisis, an ATRX-targeted Clustered Regularly Interspaced Palindromic Repeat/CRISPR-associated 9 (CRISPR/Cas9) vector was used to functionally inactivate the ATRX gene [30]. Following transfection with the ATRX CRISPR/Cas9 vector, single-cell clones were isolated and monitored as they transited through crisis (Figs 1A and 1B and S1A and S1B). A total of 6 and 9 clones survived crisis for the HCA2 and MRC5 cell lines respectively (11.5% and 7.1% survival rate, n = 52 and n = 127 respectively). ATRX protein expression was monitored by Western blot analysis to establish the effects of the CRISPR vector on cell survival. Strikingly, clones that retained ATRX protein expression failed to escape crisis, whilst a complete loss of the ATRX protein resulted in replicative immortality in both cell lines (Figs 1C and 1D and S1C). The status of the ATRX gene was sequence verified in two randomly selected clones: clone 10 and clone 18, which confirmed that a clone (clone 10) that did not escape, expressed wild-type ATRX (likely due to incomplete cutting by CRISPR) whereas a clone that did escape (clone 18), did not express ATRX due to a -2 bp frameshift (S2 Fig).

The immortalisation was dependent upon the loss of ATRX expression as, consistent with numerous previous observations [35,40], HCA2^HPVE6E7^ control clones (n = 6), which express a WT ATRX failed to escape crisis and died after a prolonged period of crisis (S3 Fig). In conclusion, it appeared as if even low-level residual ATRX activity hindered the ability of cells to escape crisis whereas a complete loss of ATRX correlated with successful long-term survival (*i*.*e*., immortalisation) in primary human fibroblasts (Figs 1A–1D and S1A–1C)).

Telomere dynamics were analysed at the XpYp and 17p chromosome ends using STELA (Figs 1E and 1F and S1D and S1E; and S4). Consistent with previous observations in clonal WT fibroblast cultures [35,41], homogeneous allelic telomere-length distributions were observed in clones that failed to escape crisis, as well as control clones, with all telomeric alleles exhibiting a gradual loss of telomere length as cells approached crisis (Figs 1E and S1D and S4). In stark contrast, the clones that escaped crisis upon loss of ATRX displayed heterogeneous telomere lengths, with no distinguishable allelic telomere length distributions, even at the earliest sampling points (Figs 1F and S1E and S5). MRC5 cells exhibit telomere-adjacent sequence polymorphisms that allow for allele-specific (GC or AT) STELA at the XpYp telomere [41]. MRC5 clones 121, 9 and 46 that escaped crisis in the absence of ATRX, displayed heterogeneous telomere-length distributions at both alleles (S1E and S5B Figs), in contrast to clone 1, that failed to escape crisis and maintained distinct homogeneous allelic distributions (S1D Figs). Therefore, the loss of ATRX induces telomere length heterogeneity during crisis at both alleles, irrespective of their length prior to crisis, and this heterogeneity is maintained following their escape from crisis, suggesting that these cells may have induced an ALT-like phenotype.

Telomere fusion analysis of HCA2 clones revealed that fusion events could readily be detected, even at the earliest sampling points, indicating that these cells had entered a telomere crisis and thus the generation of telomere length heterogeneity occurs within the period of crisis (S6 Fig). These data also demonstrate that short telomeres during crisis, in the absence of ATRX, are subjected to repair activity, as observed in wild-type cells undergoing crisis [33,35]. Moreover, as the cells escaped crisis, the frequency of fusion events was reduced in all but one clone (S6B Fig; clone 49) and the analysis of post-crisis ALT^+^ U2OS cells revealed no detectable telomere fusion events despite these cells exhibiting extreme telomere length heterogeneity. These data indicate that the telomere fusions occur early in crisis and that this is likely necessary (clones 18, 21, 27, 28 and 49) but not sufficient (clone 1) for the establishment of an ALT phenotype. Moreover, the data indicate that once ALT is established, it is sufficient to prevent the subsequent fusion of short telomeres even though they are relatively abundant.

One of the hallmarks of ALT is the presence of extrachromosomal partially single-stranded DNA, referred to as C-circles [37]. We used a C-circle assay to establish whether the telomere elongation events observed during crisis may coincide with the presence of C-circles and thus be consistent with ALT activity [37]. C-circles were absent in the parental HCA2 and MRC5 cells, but increased during the escape from crisis in all HCA2^HPVE6E7;ATRX-/o^ and MRC5^HPVE6E7;ATRX-/o^ clones to levels greater than that observed in the ALT-positive control U2OS (Figs 1G, S1F and S7). In contrast, clones that failed to escape crisis were negative for C-circles. In addition, telomerase activity was not detected in any of the fibroblast clones following immortalisation, irrespective of whether they subsequently escaped crisis or not (S8 Fig). Taken together these data reveal a key role for ATRX in suppressing the ability of fibroblast cells to escape a telomere-driven crisis by normally inhibiting telomeric elongation events during crisis. Moreover, the induction of ALT activity is sufficient to ultimately confer functional telomeres that, subsequent to crisis survival, are no longer subjected to fusion.

### ATRX facilitates the escape from a telomere-driven crisis in epithelial cancer cells

**Fig 2:**
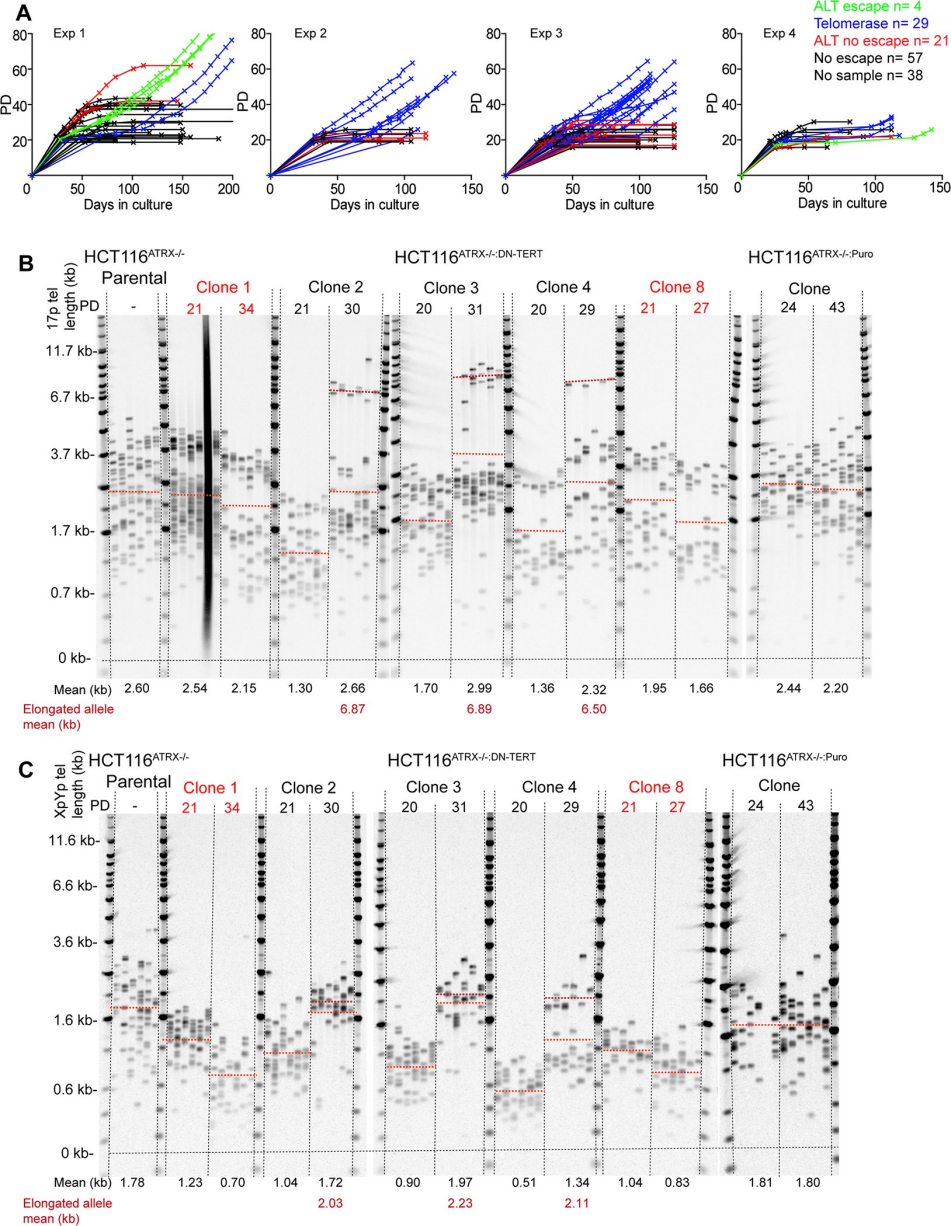
ALT-like initiation in the absence of ATRX in HCT116 epithelial cancer cells undergoing a telomere-driven “crisis”. (A) Growth curves displaying PDs versus days in culture for the 149 HCT116^ATRX-/0:DN-hTERT^ single cell clones picked across four DN-hTERT transfections represented by the four separate panels; green clones that presented ALT-like telomere elongation; blue clones are telomerase-positive escapees; red clones showed ALT-like characteristics, but did not survive; and black clones are clones that did not survive. STELA profiles at the (B) 17p and (C) XpYp chromosome ends for the HCT116^ATRX-/o^ parental; HCT116^ATRX-/o:DN-hTERT^ clones 1 and 8 that did not survive and clones 2, 3 and 4 that exhibited an ALT-like elongation event; and HCT116^ATRX-/o:Puro^ clone as a transfection control with the PD indicated across the top and the mean telomere length alongside the elongated allele mean in kb shown across the bottom; also represented as orange and red dotted lines, respectively, on the blots.

We have previously generated ATRX-null telomerase-positive human HCT116 colorectal epithelial cancer cells using both recombinant adeno-associated virus (rAAV)- and CRISPR/Cas9-mediated gene targeting [30]. We demonstrated that the genetic deletion of ATRX alone did not lead to the activation of the ALT phenotype even when these cells were forced through crisis; these cells were negative for C-circles, did not display heterogenous telomere length profiles and continued to express telomerase [30]. Thus, the loss of ATRX had a different outcome for the non-mesenchymal HCT116 epithelial cells than it did for the mesenchymal HCA2 and MRC5 fibroblast cell lines. To assess whether the loss of ATRX combined with telomere dysfunction during crisis could at least initiate the ALT mechanism for survival we transfected HCT116^ATRX-/o^ with a dominant-negative hTERT (DN-hTERT) construct [42] to abrogate telomerase activity and induce a telomere-driven crisis. A total of 149 single cell clones were picked from four separate DN-hTERT transfections; clones were continuously passaged in culture and were monitored for changes in growth rate and morphology. All the clones entered a period of crisis, defined as a slowing in the rate of expansion of the culture (Fig 2A) and a change in morphology from small, actively-dividing cells to large, multi-nucleated cells (S9 Fig). Unlike WT HCT116^DN-hTERT^ clones, in which 100% of clones (11 of 11) rapidly escaped crisis after having re-established telomerase activity [33], only 33 out of 149 (22%) of the HCT116^ATRX-/o:DN-hTERT^ clones escaped a telomere-driven crisis and appeared to gain replicative immortality (Fig 2A). These 33 clones, of which 29 appeared to be telomerase positive and 4 ALT, were cultured until a normal growth rate had resumed after which the cultures were terminated. The remaining 116 clones entered crisis and died, including 38 clones that entered crisis prior to a sample being taken (summarised in S1 Table). No evidence of crisis was observed in HCT116^ATRX-/o:Puro^ control clones (n = 12) expressing the puromycin drug resistance selection cassette (*i*.*e*., without DN-hTERT) only (S10 Fig). Thus, the absence of ATRX compromised the ability of HCT116 epithelial cells to escape crisis (where only 22% of the ATRX-null clones escaped crisis)—a phenotype diametrically opposed to the one observed in fibroblast cells, where 100% of the surviving clones were ATRX-null.

### Specific telomeric elongation events and C-circles consistent with ALT activity in the absence of ATRX

We monitored the telomere dynamics of HCT116^ATRX-/0:DN-hTERT^ clones undergoing crisis in culture. Telomere length profiles at the 17p and XpYp chromosome ends were obtained using STELA at sampling points both pre- and post-crisis for 82 clones. The majority of clones (78 clones: 95%) exhibited telomere erosion prior to crisis (mean of 60 bps/PD at 17p and 80 bps/PD at XpYp), consistent with the abrogation of telomerase activity following the expression of DN-hTERT. Strikingly, three clones displayed telomere erosion followed by, at the point of crisis, an abrupt elongation event at both chromosome ends analysed (mean of elongated allele: 6.68 kb at 17p and 1.87 kb at XpYp) (Fig 2B and 2C, clones 2, 3 and 4). A fourth clone exhibited a similar sized elongation event at the XpYp chromosome end (1.07 kb), but not at 17p (S11 Fig). The bimodal distributions observed at the 17p chromosome end before crisis were consistent with allelic telomere length variation, we thus considered that the elongated telomeres arose from the extension of a single allele. However, it was also possible that the bimodal distributions arose from subsets of cells with distinct telomere length profiles with the extension events occurring in a specific subset of cells and were thus not allelic. To test this, we subcloned clone 3 at the point of crisis and examined the telomere length distributions. All the surviving subclones displayed bi-modal telomere length distributions, consistent with allele-specific telomeric elongation (S12 Fig). Interestingly, a single subclone died (subclone 2) at PD17 whilst displaying no telomere extension events (S12 Fig).

The clones that failed to escape crisis, showed telomere erosion prior to crisis, but no change in telomere length at crisis (Figs 2B and 2C, clones 1 and 8 and S13). HCT116^ATRX-/o:Puro^ control clones displayed no significant change in mean telomere length (Fig 2B and 2C). A further 17 clones that escaped crisis, exhibited a change from homogeneous to more heterogeneous telomere-length distributions, whilst clones for which no sample was available pre-crisis (n = 8) also showed similar heterogeneity post-crisis (S14 Fig), these telomere dynamics are consistent with a reactivation of telomerase following the escape from crisis, as described [33].

**Fig 3:**
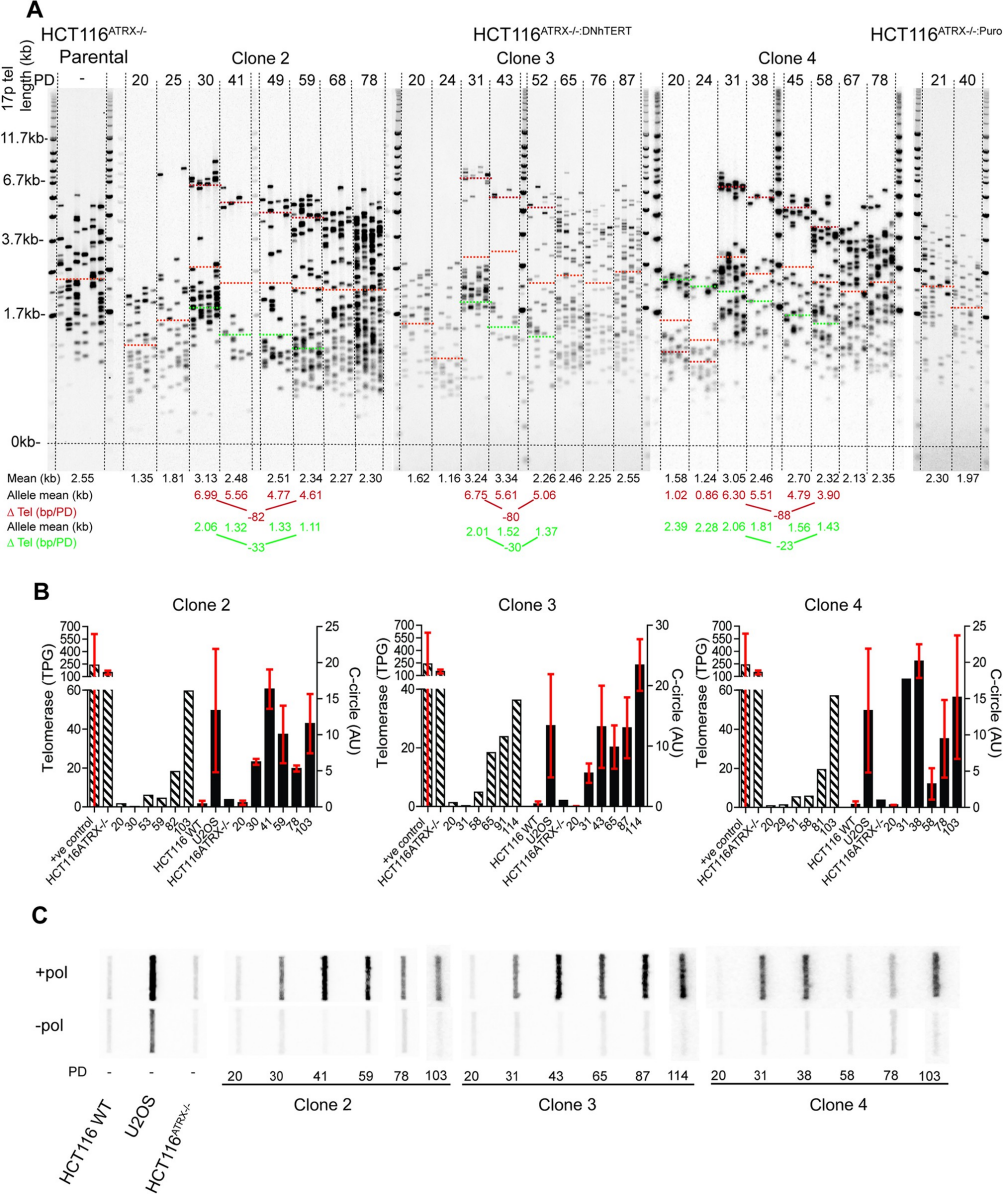
ALT-like telomere elongation and long-term telomerase upregulation in HCT116 epithelial cells. (A) STELA profiles at the 17p chromosome end for the HCT116^ATRX-/o^ parental; HCT116^ATRX-/o:DN-hTERT^ ALT-like clones 2, 3 and 4; and HCT116^ATRX-/o:Puro^ with PD points indicated above and the overall mean telomere length in black (represented as orange dotted lines on the blot) displayed below together with allelic mean telomere lengths (red and green) also represented as dotted lines on the blot. The rate of erosion is represented by ΔTel and is expressed in bp/PD. (B) Quantification of telomerase activity (expressed in total product generated: TPG) on the left axis and the C-circle intensity (expressed in arbitrary units: AU) on the right axis. SD was used as error bars and the PD is indicated on the X-axis. (C) C-circle assay slot blots with (+ pol) and without (- pol) polymerase samples with the PD and clone number shown across the bottom. Striped bars indicate telomerase activity and solid bars C-circles.

We next examined the longer-term maintenance of the extended telomeres in the three clones that had exhibited elongation at both chromosome ends studied. These clones were kept in culture for 219 days until they had obtained over 100 PDs and the telomere length profiles were examined at serial sampling points. At the 17p telomere, we observed bi-modal telomere-length distributions pre- and post-crisis consistent with two telomeric alleles [41]. We hypothesised that the short allele, prior to crisis, became elongated to a mean of 6.68 kb (*i*.*e*., an extension of 5.40 kb) whilst the longer allele continued to erode (Fig 3A). At the XpYp telomere, a single allele was detected that underwent elongation to a mean of 1.87 kb (*i*.*e*., an extension of 1.06 kb) (S15 Fig). Following the initial elongation event, both telomeres continued to erode, with the longer telomeric allele at 17p exhibiting an erosion rate (mean for all 3 clones examined: 83 bps/PD, Fig 3A) that was indistinguishable from that observed in primary cells in the absence of telomerase [41], whilst the shorter 17p telomeric alleles exhibited a slower rate of erosion (mean for all 3 clones examined: 29 bps/PD). The single XpYp telomeric allele was also subject to telomere erosion (mean rate of erosion of 27 bps/PD; S15 Fig). Telomere erosion continued until the cultures upregulated telomerase at PD 59, 60 and 58 in clones 2, 3 and 4, respectively, as determined by the TRAP assay. The restoration of telomerase activity was accompanied by an increase in the heterogeneity of the telomere length distributions resulting in a loss of bimodal distributions and an increase in mean telomere length (Fig 3A and 3B). These telomere dynamics were recapitulated in subclone 11 and were consistent with the action of telomerase preferentially elongating the shorter allele, but not the longer allele (S12 Fig) [36,43].

To assess if the initial elongation event observed was consistent with ALT upregulation, clones were subjected to the C-circle assay. The intensity of the signal was quantified in duplicate and all results were normalised to the HCT116^ATRX-/o^ parental cell line background. The three clones (clones 2, 3 and 4) that displayed elongation at the XpYp and 17p telomeres showed a gradual increase in C-circles from the initial telomere elongation event, although these cells continued to exhibit C-circles during the re-establishment of telomerase activity (Fig 3C). A further 3 clones (32, 48 and 131), for which no telomeric elongation event was observed at the XpYp telomeres, were also strongly positive for C-circles post-crisis, a state which diminished at later PDs (S16A–S16C Fig); these cells also upregulated telomerase activity (S16D Fig). Overall the levels of C-circles was consistently less than that observed in the ALT-positive cell line U2OS and less than that observed in fibroblast cultures (S7 Fig). In summary, the presence of C-circles correlated with the ALT-like telomere elongations observed in clones 2, 3 and 4 but C-circles were also present in clones 32, 48 and 131, which did not elongate. Thus, we concluded that while the presence of C-circles might be necessary for these ALT-like elongations, they aren’t sufficient.

It was clear that in the HCT116^ATRX-/o:DN-hTERT^ system, single telomeric elongation events were not sufficient to confer replicative immortality following the escape from crisis. We therefore considered that other clones that failed to escape crisis may also have shown evidence of telomeric elongation events. On this basis, STELA profiles were obtained at 17p and XpYp for all clones that died and for which samples where available (n = 46). Two clones (108 and 132) exhibited elongation events at the XpYp telomere of 0.74 kb on average, similar to that observed in clones 2, 3, 4 and 147 (S17A Fig). No evidence of telomeric extension was observed at the 17p chromosome end in these two clones, instead the shorter telomeric allele was lost during crisis (S17B Fig). Both these clones were positive for C-circles (S17C and F17D Fig) whilst telomerase activity was undetectable (S17D Fig). Importantly, these data imply that neither C-circle activity, nor single telomeric elongation, is sufficient for these cells to escape a telomere-driven crisis. All HCT116^ATRX-/o:DN-hTERT^ clones that ultimately obtained replicative immortality did so by regaining telomerase as their principle TMM.

Overall, these data indicate that the combination of the loss of ATRX and telomere dysfunction during crisis is sufficient to initiate an “ALT-like” mechanism in a subset of clones (21% of surviving clones and 16% of clones that died; 19% of total clones). Whilst this rapid telomere elongation and C-circle activity is “ALT-like”, in the HCT116 DN-hTERT model system this is insufficient for the maintenance of ALT activity and telomerase activity is ultimately required for long-term survival.

### Chromosome specific elongation

**Fig 4:**
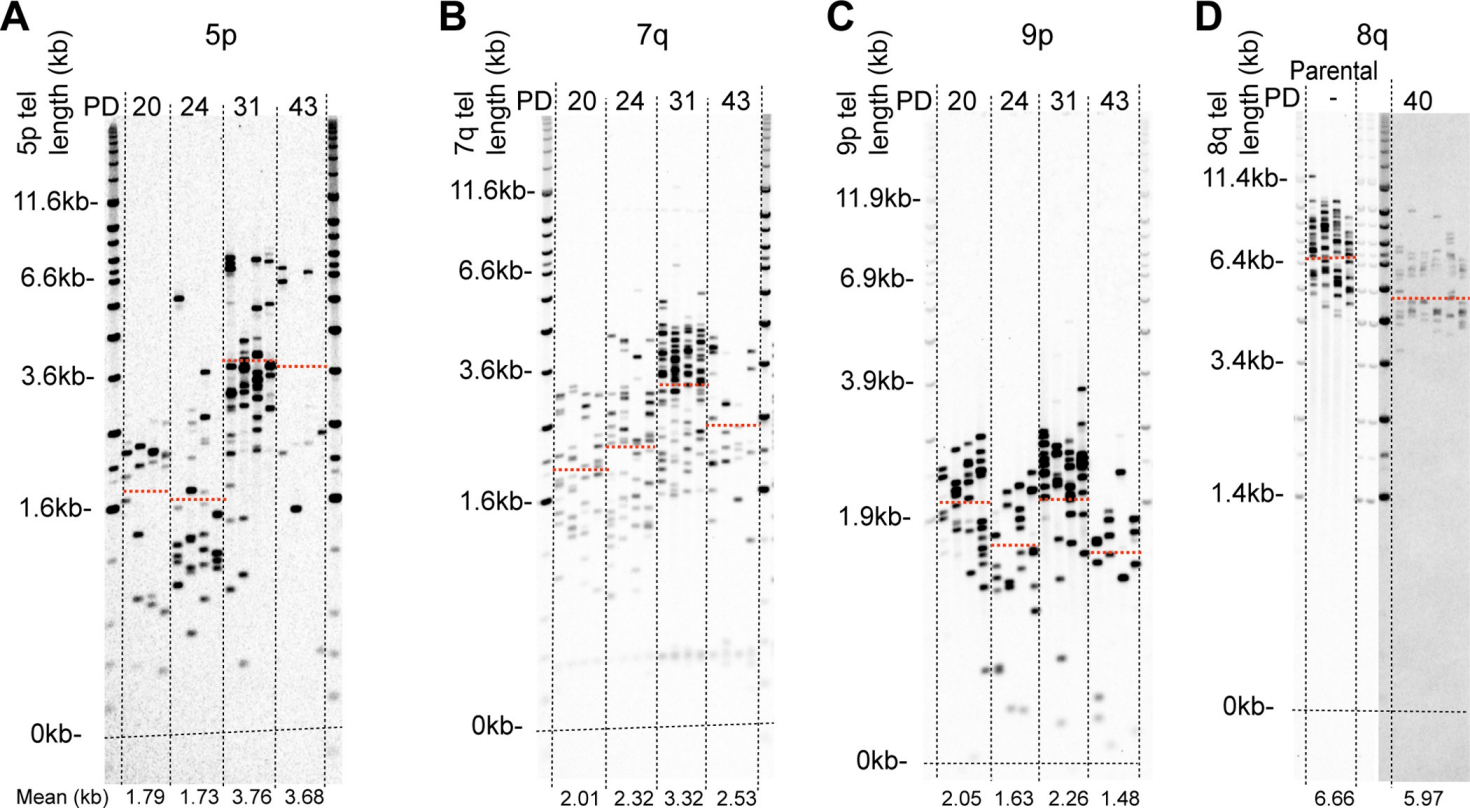
Chromosome specific elongation of short telomeres upon ALT upregulation in HCT116 epithelial cells. STELA profiles at the (A) 5p, (B) 7q, (C) 9p and (D) 8q chromosome ends for HCT116^ATRX-/o:DN-hTERT^ ALT-like clone 3 alongside the HCT116^ATRX-/o^ parental clone at the 8q chromosome end for reference with the PD indicated across the top and the mean telomere length indicated in kb across the bottom and also represented as orange dotted lines on the blot.

Having observed telomeric elongation events in multiple HCT116^ATRX-/o:DN-hTERT^ clones that appeared to be chromosome specific, with a mean of 5.40 kb added to the shorter allele at 17p and 1.06 kb at the single XpYp allele (Fig 2B and 2C), we investigated whether chromosome-specific telomeric elongation events occurred at other chromosome ends. To address this, we applied STELA at the 5p, 7q, 8q and 9p chromosome ends in clones that showed telomere elongation at both the XpYp and 17p chromosome ends (clones 2, 3 and 4) (Figs 4 and S18). We observed specific elongation events in all three clones at the 5p, 7q and 9p telomeres, with mean extensions to: 3.58 kb (extension of 1.9 kb) at 5p; 3.22 kb (extension of 1.14 kb) at 7q; 1.93 kb (extension of 0.52 kb) at 9p (S18D Fig). In contrast, no extension events were observed at the telomeres at the 8q chromosome end, which were long relative to the other telomeres analysed (6.66 kb) in the parental cell line; instead, this telomere simply eroded as a function of cell division post-crisis. Thus, there appeared to be a lack of unified specificity in the telomere elongation, with 5 of the 6 telomere ends examined demonstrating telomere elongation. With that said, there clearly was a rather profound difference in the amount of telomere elongation associated with each end, ranging from a ~5.5 kb addition at 17p to only ~0.5 kb at 9p. The basis for the variable elongation is not understood, but most chromosome ends appeared affected.

### Elongation affects all alleles and arises from multiple independent events

Our STELA data indicated that some HCT116 clones might be ALT-like and that short telomeric alleles were specifically elongated during crisis. To establish if this was the case, and to examine the nature of telomere-specific extensions, we characterised telomeric alleles using PacBio single-molecule real time (SMRT) long-read sequencing of multiplexed STELA amplicons from the 7q, 17p and XpYp telomeres obtained by pooling 1,600 reactions for each sample analysed (HCT116^ATRX-/o^ parental and clone 3; HCA2^HPVE6E7^ parental and clone 21^ATRX-/o^; and U2OS). We utilised the hypervariable telomere variant repeat (TVR) patterns within the first 100 base pairs of the telomere repeat array to differentiate telomeric alleles from HCT116, HCA2 and U2OS cells [44–46]. The TVR content was determined for each read to establish any differences in variant repeat distributions between the ALT-like clones and their respective parental controls. We observed an overall increase in TVRs in the ALT-like clone, with a specific enrichment of TTCGGG TVR in the HCT116 clone at all chromosome ends and alleles analysed (S19A Fig), whilst an increase in the TGAGGG TVR was observed in the HCA2 clone analysed that was most apparent at the XpYp chromosome end (S19B Fig). Consistent with previous findings [47], the telomeric alleles in U2OS contained relatively few TVRs being composed predominately of TTAGGG repeats (S19C Fig). These data indicated that the increase in TVR interspersion patterns were specific to each cell clone and consistent with a utilisation of a clone-specific telomeric DNA template.

**Fig 5:**
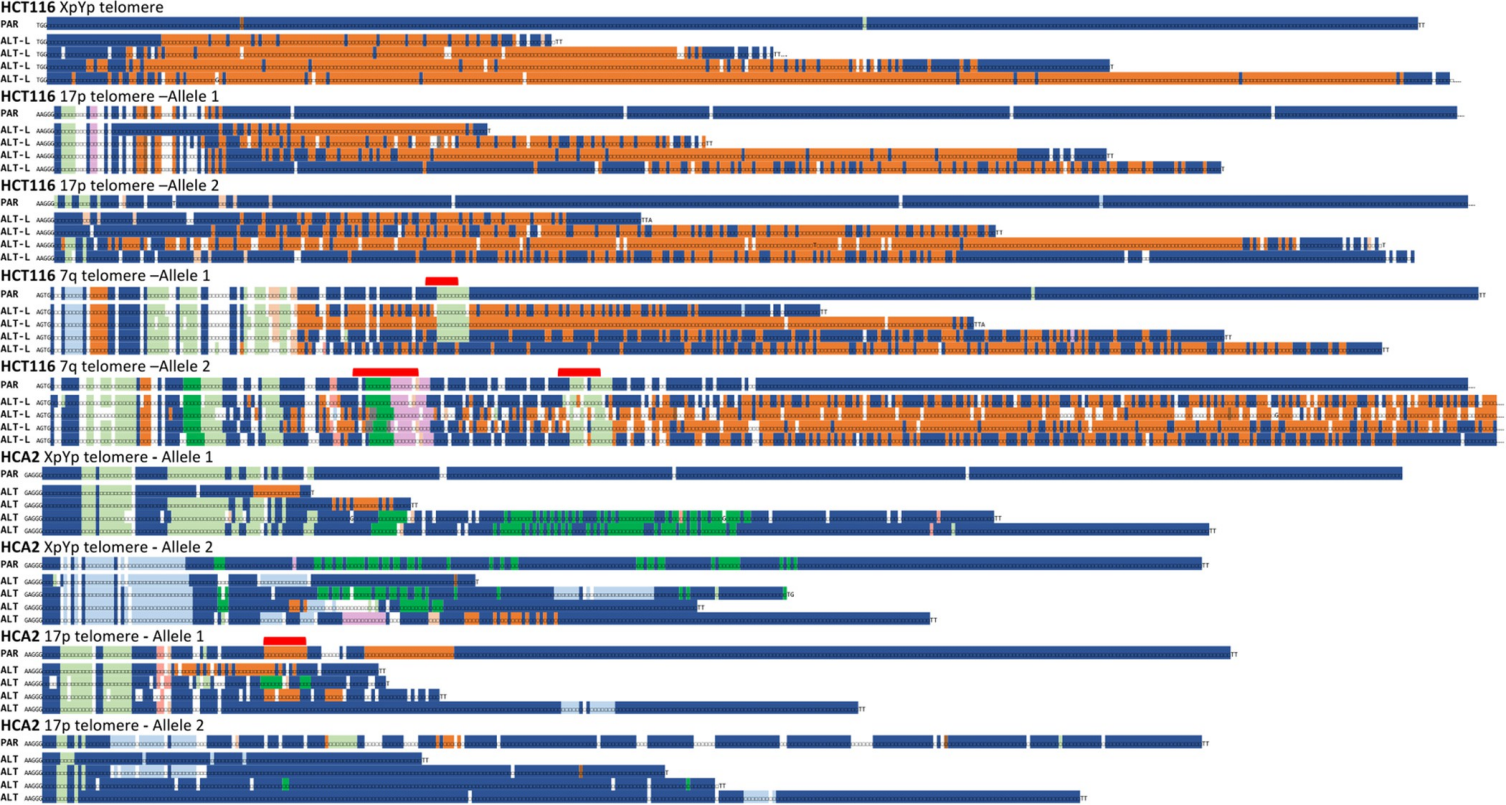
ALT elongation arises from multiple independent events. Examples of telomere sequences obtained from PacBio sequencing of STELA amplicons obtained from HCT116^ATRX-/o^ and HCA2^HPVE6E7;ATRX-/o^ cells at the XpYp, 17p and 7q chromosome ends prior to crisis (Parental) and a single clone of each exhibiting characteristics of ALT post crisis, denoted as ALT from HCA2^HPVE6E7;ATRX-/o^ fibroblasts and ALT-L (ALT-like) from HCT116^ATRX-/o^. Each telomeric allele is displayed separately with the parental (PAR) telomeric allele above and the derived ALT allele (ALT) below. The red bar displayed above the parental allele sequences indicates distinct TVR patterns conserved in ALT alleles. The telomere and 6 nt TVR sequences are coded as follows: dark blue □ = TTAGGG; light blue □ = TCAGGG; dark orange □ = TTCGGG; beige □ = GTAGGG; dark green □ = TGAGGG; light green □ = TTGGGG; ochre □ = TAAGGG; lilac □ = CTAGGG; brown □ = TTTGGG; yellow □ = AGAGGG; white □ TVRs ≶ 6 nt.

Examination of the sequence composition of individual telomeric alleles revealed several notable features (Figs 5 and S20). In HCT116 cells, a dramatic increase in TTCGGG variant repeats was observed in all alleles, at each of the three telomeres analysed, irrespective of the length of the elongated allele. In HCA2 cells, an insertion of a characteristic TGAGGG interspersion pattern was observed in XpYp allele 1, with different TVR patterns in XpYp allele 2 and both 17p alleles sequenced. Together these observations indicate that the insertion of TVRs does not lead to specific elongation events, with alleles exhibiting changes in TVR composition in the absence of elongation events. Moreover, each individual telomere sequenced displayed distinct TVR interspersion patterns that disrupted the parental allele at different points with respect to the beginning of the telomere repeat array. These data indicate the occurrence of multiple different mutational events, occurring within different telomeric alleles, with the majority of events resulting in the replacement of distal sequences, leaving the repeat distributions at the beginning of the telomere repeat array intact (Figs 5 and S20). The diversity of TVR distributions within a cell and between cells that escape crisis in the absence of ATRX is consistent with the view that telomeric mutation during crisis occurs multiple times and in multiple independent cells.

### Initiation of ALT in HCT116^ATRX-/o:DNhTERT^ is not associated with increased genomic complexity

The transit through a telomere crisis is associated with the induction of increased genomic complexity with distinct topologies observed in the context of specific DNA repair deficiencies [33,39,48]. We therefore examined whether the absence of ATRX modulates genomic complexity in cells that escape crisis and whether this was impacted by the telomeric elongation events observed. We generated whole genome sequence data from HCT116^ATRX-/o:DNhTERT^ clones that exhibited “ALT-like” telomere elongation, sampling the cells pre- and post-crisis. We also analysed clones that showed no evidence of telomere elongation, nor C-circles, but had upregulated telomerase activity during crisis (total n = 30). All HCT116^ATRX-/o:DNhTERT^ clones displayed genome stability prior to crisis, as did all the clones that exhibited transient “ALT-like” activity. Consistent with our previous observations [39], all the HCT116^ATRX-/o:DNhTERT^ clones that had upregulated telomerase showed a higher rate of structural variants, including an example of chromothripsis (S21 Fig). Thus, whilst the initiation of “ALT-like” activity in HCT116^ATRX-/o:DNhTERT^ cells undergoing crisis in the absence of ATRX is associated with rapid, telomere-specific elongation, this did not appear to be accompanied by large-scale genomic copy number changes, or at least no more so than was observed in immortalised clones lacking the “ALT-like” phenotype.

## Discussion

A strong link between ATRX and the ALT phenotype in various malignancies and cell culture models has been well established [21,28,30,49]. Whilst there has been a focus on the association of ATRX with such phenotypes or mechanistic contributions to repressing ALT activity, thus far it has only been demonstrated once that the loss ATRX alone is sufficient to induce ALT, and only in specific glioma cell lines [50]. This lack of ALT inducing-activity in ATRX-null cells was surprising particularly given the association of the lack of ATRX expression with ALT cancers. Adding to this complexity was the finding that genetic ablation of another histone H3.3-H4 dimer chaperone, anti-silencing function 1 (ASF-1), readily generated ALT-activity in telomerase-positive cells [51]. Here we show that the loss of ATRX allows primary human fibroblast cultures undergoing a telomere-driven crisis to readily escape and gain replicative immortality, following the establishment of ALT-activity. Importantly, even low-level, residual ATRX activity, is sufficient to prevent these cells from initiating ALT and achieving immortality (Fig 2C). In contrast, the loss of ATRX alone was not sufficient to induce ALT in epithelial cancer cells when experimentally manipulated to undergo a second crisis. Nonetheless, even in these cells, an “ALT-like” phenotype could be initiated, albeit ultimately not stably maintained. These data are consistent with the view, that ATRX is a *bona fide* ALT inhibitor in the context of telomere dysfunction [31]. Moreover, by demonstrating that there are different immortalisation outcomes depending upon the type of cell in which the loss of ATRX activity occurs, we provide clarity to a literature that was opaque.

ATRX plays an important role in replication fork protection and restart, and its loss leads to an increase in replication fork stalling and collapse [52,53]. Furthermore, ATRX, along with its binding partner DAXX, prevents the accumulation of secondary structures such as R-loops and G quadruplexes, that arise at repetitive regions of the genome (including telomeres), by incorporating histone H3.3 into nucleosomes [54,55]. These secondary structures form a further barrier to the replication fork machinery thereby increasing replication stress, which can be repaired by BIR [54,56]. Somewhat counterintuitively, replication stress is actually required in ALT cells because the repair induced by BIR leads to telomeric elongation. We therefore propose that the loss of ATRX and the presence of short telomeres during crisis may trigger telomere lengthening via the accumulation of replication stress and the induction of BIR at telomeres [57]. A corollary of this conclusion is that the factors required for replication stress and/or BIR are likely to vary between fibroblasts (more permissive) and epithelial (non-permissive) cells. Our model also postulates that mutations that diminish or inactivate BIR are likely to impede the establishment of ALT; a model that we are currently trying to test. While we believe that differences in BIR is the most likely, and parsimonious, explanation for the cell type and TMM disparities observed in these studies, it should be emphasised that the distinction between fibroblastic and epithelial cell lines we have observed could also be related to the fact that the HCA2 and MRC5 fibroblast cell lines had not, unlike the HCT116 epithelial cell line, undergone a previous transformation event. The impact of a previous transformation event on the ability of a cell to establish a new TMM is unknown, but clearly worth future investigation.

Our data show that the ALT mechanism induces elongation events at multiple chromosome ends only in cells that have entered a telomere-driven crisis. In fibroblasts, telomere lengthening and heterogeneity were observed in all telomeric alleles, whereas in epithelial cancer cells, lengthening appeared to be more specific to the shortest alleles. The underlying mechanism for these differences is not apparent from our data, although, it has recently been shown, through single molecule analysis, that telomere length and content heterogeneity varied according to the sub-telomere studied in a panel of ALT cell lines [58], consistent with chromosome-specific events. However, our study suggests a different possibility; namely that the difference may be a manifestation of different levels of BIR factors/ALT activity given that fibroblasts fully activated the ALT pathway, whereas, the cancer epithelial cells only transiently activated it. In this situation, the extreme telomere length heterogeneity generated following the full activation of the ALT pathway may mask subtler and specific elongation events that may be more apparent at lower levels of activity. Interestingly, our data suggest that these events are chromosome specific, as independent clones exhibited similar elongation events at each telomere in the HCT116 model. We hypothesise that telomeric elongation may be regulated, with a specific and consistent DNA fragment length, consisting of telomere variant repeats derived from telomeric and interstitial telomeric sequences [59] being inserted into the telomere, potentially mediated by a recombination-based mechanism [16,19,60–62] and/or by BIR, which is known to be highly mutagenic. Consistent with recombination-based mechanisms, a recent study has shown the presence of large linear extrachromosomal DNA, alongside the C-circles and short linear tracts already well established as ALT markers, that accounts for 40% of the total telomere signal in U2OS. These DNA structures have the potential to play an active role in telomere maintenance by acting as templates in BIR-mediated lengthening [58].

Alterations in TVR patterns in ALT clones were specific to each cell clone, which is consistent with previous studies investigating the telomere repeat content in ALT-positive cancer samples [63,64]. We therefore hypothesise that a common initiating event is required to seed the clone-specific TVR patterns, forming ALT precursors as described in yeast models [62] that subsequently provide a template to enable elongation of short telomeres by inter-allelic exchanges [16,61]. Interestingly, TVR replacement was also observed both proximally and distally to distinctive conserved TVR patterns (highlighted in red, Fig 5), indicating a possible gene conversion type mechanism that can conserve existing TVR patterns, although this remains to be fully tested. Whilst normal human cells harbour non-canonical variant repeat interspersion patterns within the proximal 2 kb of the telomere repeat array [45], ALT telomeres display TVRs throughout the telomere [65]. It is considered that these enable the anchoring of nuclear receptors such as COUP-TF2, which play a role in the ALT phenotype [65]. We observed a similar interspersion pattern of variant repeats in the fibroblast ALT-like clones. Interestingly, PacBio sequencing of the HCT116 ALT-like clones showed a stark increase in the TTCGGG variant repeat, even at the distal end of the chromosome. The presence of these non-canonical TVRs may reduce the binding of the telomere-associated proteins telomere recognition factors 1 and 2 (TRF1 and TRF2) to the telomere and compromise telomere function. Despite that our data provides some insight in telomere variant repeat pattern content, more remains to be done to elucidate the mechanism of elongation in the context of ALT.

In summary, in fibroblasts, the induction of ALT in the absence of ATRX occurs early in crisis at the point that telomere fusions are detected. This facilitates a rapid and efficient escape from crisis, the abrogation of telomere fusions and genomic stabilisation. We suggest these may represent the types of events that occur during the initiation of ALT-positive tumours. Further understanding of the upregulation of the ALT pathway and the mechanism of elongation is crucial for the development of treatments as well as diagnostic and prognostic tests.

## Supporting information

S1 Fig ALT activation and maintenance in the absence of ATRX in MRC5 primary fibroblasts undergoing a telomere-driven “crisis”. Growth curves displaying population doublings (PDs) against days in culture for (A) MRC5^HPVE6E7^ clones (n = 5) that failed to escape crisis and (B) MRC5^HPVE6E7^ clones (n = 9) that successfully escaped crisis. (C) Western blots displaying ATRX protein expression in “no escape” and “escape” clones with Vinculin expression used as loading control. STELA profiles (overall and GC or AT allele-specific) at the XpYp chromosome end for clone 1 (D) that failed to escape crisis and clone 121 (E) that successfully escaped crisis; with the PD points stated across the top and the mean of the telomere length distributions detailed across the bottom, with the mean also represented as orange dotted lines on the blot. (F) C-circle assay slot blots of the with (+ pol) and without (- pol) φ29 DNA polymerase samples with the PD and clone number stated across the bottom.
(DOCX)

S2 Fig Sequence verification of selected clones exposed to ATRX CRISPR. Examples of three HCA2^HPVE6E7^ clones that presented a mutated ATRX gene upon screening that were subsequently analysed by sequencing. In yellow is indicated the CRISPR target site as well as a Sml1 restriction site; dashes represent deletions.
(DOCX)

S3 Fig Induction of a telomere-driven crisis upon transfection of E6E7 viral oncoproteins in fibroblasts WT for ATRX. Growth curves displaying population doublings (PDs) against days in culture of the 6 HCA2^HPVE6E7^ single cell clones.
(DOCX)

S4 Fig Homogeneous telomere length distributions in control and no escape clones. STELA profiles at the XpYp and 17p chromosome ends in HCA2^HPVE6E7^ cells for (A) clones 1 and 4 used as controls and (B) clones 2 and 44 that failed to escape crisis; with the PD points stated across the top and the mean of the telomere length distributions detailed across the bottom, with the mean also represented as orange dotted lines on the blot.
(DOCX)

S5 Fig Heterogeneous telomere length distributions upon loss of ATRX and escape from crisis. STELA profiles for (A) HCA2^HPVE6E7 ATRX-/-^ clones 18 and 21 at the XpYp and 17p chromosome end; and (B) MRC5^HPVE6E7 ATRX-/-^ clones 9 and 46 at the XpYp chromosome end for the combined alleles as well as for specific (GC or AT as indicated above) alleles; with the PD points stated across the top and the mean and standard deviation of the telomere length distributions detailed across the bottom, with the mean also represented as orange dotted lines on the blot.
(DOCX)

S6 Fig Fusion profiles reveal an increase of end-to-end fusions in escapees during crisis. A) Example of fusion profiles for clone 1 (no escape) and clone 21 (escape) across multiple PD points (detailed across the top) and U2OS as an ALT-positive control. Blots were serially Southern hybridised with the telomere-adjacent DNA probes indicated on the left and the number of unique fusions stated below the blots. B) Bar charts depicting the number of XpYp (purple), 17p (white), 21q (grey) and total (black) fusion events as escapees are transiting through crisis and immortalising. (Number of diploid genome equivalents analysed = 2 x 10^4^).
(DOCX)

S7 Fig Quantification of the C-circle slot blot intensity by subtracting the background (-pol) to the +pol sample and normalised to the HCA2^HPVE6E7;ATRX-/o^ fibroblasts parental cell line expressed in arbitrary unit (AU). The PD and clone number are stated across the bottom.
(DOCX)

S8 Fig An absence of detectable telomerase activity in fibroblast cells that escaped crisis in the absence of ATRX. TRAP assay results at the indicated PD points after the escape from crisis in A) HCA2^HPVE6E7 ATRX-/-^ cells and B) MRC5^HPVE6E7 ATRX-/-^ cells with the WT HCT116 cell line used as a positive control.
(DOCX)

S9 Fig Crisis induces visible phenotypic changes to cells. 4X magnification of HCT116^ATRX-/-:DN-hTERT^ cells (A) prior to crisis where they display small and healthy morphologies; and (B) large and multi-nucleated cells characteristic of cells undergoing crisis.
(DOCX)

S10 Fig Cell growth of puromycin control HCT116^ATRX-/-^ clones. Growth curve displaying PDs against days in culture for the puromycin control clones (n = 12) that were transfected with a puromycin selection gene to query the effects of a viral integration on HCT116^ATRX-/-^ cells survival.
(DOCX)

S11 Fig ALT-like elongation does not occur at all short telomeres. STELA profiles at the XpYp and 17p chromosome ends for HCT116^ATRX-/-:DN-hTERT^ clone 147 that underwent telomere elongation at the XpYp chromosome end, but not at 17p, despite achieving replicative immortality. PD points are detailed across the top and the overall mean telomere length in black (represented by orange dotted lines on the blot) together with the estimated allelic telomere length distributions (red and green) across the bottom, also represented as dotted lines on the blot.
(DOCX)

S12 Fig Telomeric elongation during crisis is allele specific. 17p STELA of sub-clonal populations from Clone 3 (PD31) that successfully escaped crisis following ALT-like elongation of short telomeres. PD points from the point of single-cell cloning are indicated above, with the allele-specific mean telomere length detailed below. Sub-clone 2 highlighted in red died at PD17. Sub-clone 11 was serially passaged in culture, Δ telomere allelic telomere lengths are detailed below.
(DOCX)

S13 Fig Telomere erosion following the expression of DN-hTERT in HCT116^ATRX-/-:DN-hTERT^ clones. STELA profiles at the (A) 17p and (B) XpYp chromosome ends with the PD stated across the top and the overall mean telomere length in black (represented as orange dotted lines on the blot), the longer allele in green, the shorter allele in red across the bottom also represented as dotted lines on the blot. The rate of erosion is represented by ΔTel in bp/PD. (C) Scatter plot depicting the mean telomere length of all available samples at the first sampling point and last sampling points for the 17p and XpYp chromosome ends with the p value stated above derived from a Mann-Whitney test (p-value < 0.05, highlighted in red).
(DOCX)

S14 Fig Increased heterogeneity of telomere length distributions upon the escape from crisis in HCT116^ATRX-/-:DN-hTERT^ clones. STELA profiles at the (A) XpYp and (B) 17p chromosome ends for clones 92 and 111 which successfully escaped crisis activity with the PD stated across the top and the overall mean telomere length and standard deviation in black (represented by orange dotted lines on the blot), the longer allele in green, the shorter allele in red across the bottom also represented as dotted lines on the blot. (C) Scatter plot depicting the standard deviation for all available escaping clones before (black circles) and after crisis (black triangles) or after crisis only if no pre-crisis sample was available (red triangles) at the XpYp and 17p chromosome ends. The p-value stated above were derived from a student’s t-test (p-value < 0.05, highlighted in red).
(DOCX)

S15 Fig Consistent ALT-like telomere elongation at the XpYp chromosome end in HCT116^ATRX-/-:DN-hTERT^ clones following the escape from crisis. STELA profiles at the XpYp chromosome end for the HCT116^ATRX-/-^ parental and the HCT116^ATRX-/-:DN-hTERT^ ALT-like clones 2, 3 and 4 with the PD stated across the top and the mean telomere length in black (represented as orange dotted lines on the blot) and the allele that underwent telomere extension in red across the bottom also represented as dotted lines on the blot. The rate of erosion is represented by ΔTel in bp/PD.
(DOCX)

S16 Fig C-circles detected in the absence of telomeric elongation in HCT116^ATRX-/-:DN-hTERT^ clones that escaped crisis. (A) C-circle assay slot blots with (+ pol) and without (- pol) polymerase with the PD and clone number stated across the bottom. (B) Quantification of the slot blot intensity by subtracting the background (-pol) to the +pol sample and normalised to the HCT116^ATRX-/-^ parental cell line expressed in arbitrary unit (AU) with the standard deviation used as error bars. The PD and clone number are stated across the bottom. (C) STELA profiles at the XpYp chromosome end with the PD across the top and the mean telomere length across the bottom also represented as orange dotted lines on the blot. (D) Telomerase activity quantification expressed in total product generated (TPG) with the standard deviation used as error bars where possible.
(DOCX)

S17 Fig ALT-like activity does not always confer replicative immortality. STELA profiles at (A) XpYp and (B) 17p chromosome ends for HCT116^ATRX-/-:DN-hTERT^ clones 108 and 132, which underwent an ALT-like elongation at XpYp, but failed to escape crisis. PD is detailed across the top; the mean telomere length in black (represented as orange dotted lines on the blot), the shorter allele prior to crisis in red and the longer allele prior to crisis in green across the bottom also represented as dotted lines on the blot. (C) C-circle assay slot blots with (+ pol) and without (- pol) polymerase samples with the PD and clone number stated across the bottom. (D) Quantification of C-circle intensity by subtracting the background (-pol) to the +pol sample normalised to the HCT116^ATRX-/-^ parental expressed in arbitrary unit (AU) with the standard deviation used as error bars. The PD and clone number is stated across the bottom.
(DOCX)

S18 Fig Consistent chromosome-specific elongation of telomeres. STELA profiles at the 5p, 7q, 9p and 8q chromosome ends for HCT116^ATRX-/-:DN-hTERT^ (A) clone 2 and (B) clone 4 with the PD across the top and the mean telomere length across the bottom also represented as orange dotted lines on the blot. (C) Scatter plot displaying the elongated telomere distributions at the XpYp, 17p, 7q, 5p and 9p chromosome ends of the three clones that successfully escaped crisis using the ALT mechanism with standard deviation used as error bars. (D) Scatter plot displaying the insertion lengths (mean telomere length post-elongation minus the mean telomere length prior to crisis) at the XpYp, 17p, 7q, 5p and 9p chromosome ends.
(DOCX)

S19 Fig Altered telomere variant repeat patterns in ALT-positive clones. The total proportion of specific variant repeats combining all reads expressed in percentage (calculated by combining and averaging the number of a specific variant normalised to the telomere length) for the parental and the ALT clone with corresponding bar charts expressing the fold change in variant repeat proportion when comparing parental and ALT clone using a log scale (with the score of 1 representing no change) for (A) HCT116 model; (B) HCA2 model; and (C) U2OS.
(DOCX)

S20 Fig Insertion of TVRs is cell line specific and arises from multiple events. Examples of telomere sequences obtained from PacBio sequencing of STELA amplicons obtained from HCT116^ATRX-/o^ and HCA2^HPVE6E7;ATRX-/o^ cells at the XpYp, 17p and 7q chromosome ends prior to crisis (Parental) and a single clone of each exhibiting characteristics of ALT post crisis, denoted as ALT from HCA2^HPVE6E7;ATRX-/o^ fibroblasts and ALT-L (ALT-like) from HCT116^ATRX-/o^ and ALT positive U2OS cells. Telomere and 6 nt variant repeat sequences are coded as follows: dark blue □ = TTAGGG; light blue □ = TCAGGG; dark orange □ = TTCGGG; beige □ = GTAGGG; dark green □ = TGAGGG; light green □ = TTGGGG; ochre □ = TAAGGG; lilac □ = CTAGGG; brown = □ TTTGGG; yellow = □ AGAGGG; white = □ TVRs ≶ 6 nt.
(DOCX)

S21 Fig Increased rate of structural variants in telomerase escapees but not in cells exhibiting ALT-like characteristics. A) Structural variant counts for ALT-surviving, ALT-died and telomerase-positive clones with the P value as determined by a Mann-Whitney test stated above. Statistical difference highlighted in red (P value < 0.05). Clones and timepoints at which telomerase was active are highlighted in orange. B) Complex rearrangements on chromosomes 3 and 13 in ALT-like clone 147.
(DOCX)

S1 Table Summarising data from HCT116 clones. (DOCX)

S1 Methods Supplementary methods describing PacBio SMRT sequencing analysis data processing and telomere variant repeat analysis. (PDF)
