# Supplementary material for: ATRX modulates the escape from a telomere crisis
Source: PLoS Genet. 2022 Nov 9;18(11):e1010485. doi: 10.1371/journal.pgen.1010485 (PMC9678338; doi:10.1371/journal.pgen.1010485)
Supplement: S1 Fig — Growth curves displaying population doublings (PDs) against days in culture for (A) MRC5HPVE6E7 clones (n = 5) that failed to escape crisis and (B) MRC5HPVE6E7 clones (n = 9) that successfully escaped crisis. (C) Western blots displaying ATRX protein expression in “no escape” and “escape” clones with Vinculin expression used as loading control. STELA profiles (overall and GC or AT allele-specific) at the XpYp chromosome end for clone 1 (D) that failed to escape crisis and clone 121 (E) that successfully escaped crisis; with the PD points stated across the top and the mean of the telomere length distributions detailed across the bottom, with the mean also represented as orange dotted lines on the blot. (F) C-circle assay slot blots of the with (+ pol) and without (- pol) φ29 DNA polymerase samples with the PD and clone number stated across the bottom. (DOCX) [file pgen.1010485.s001.docx]

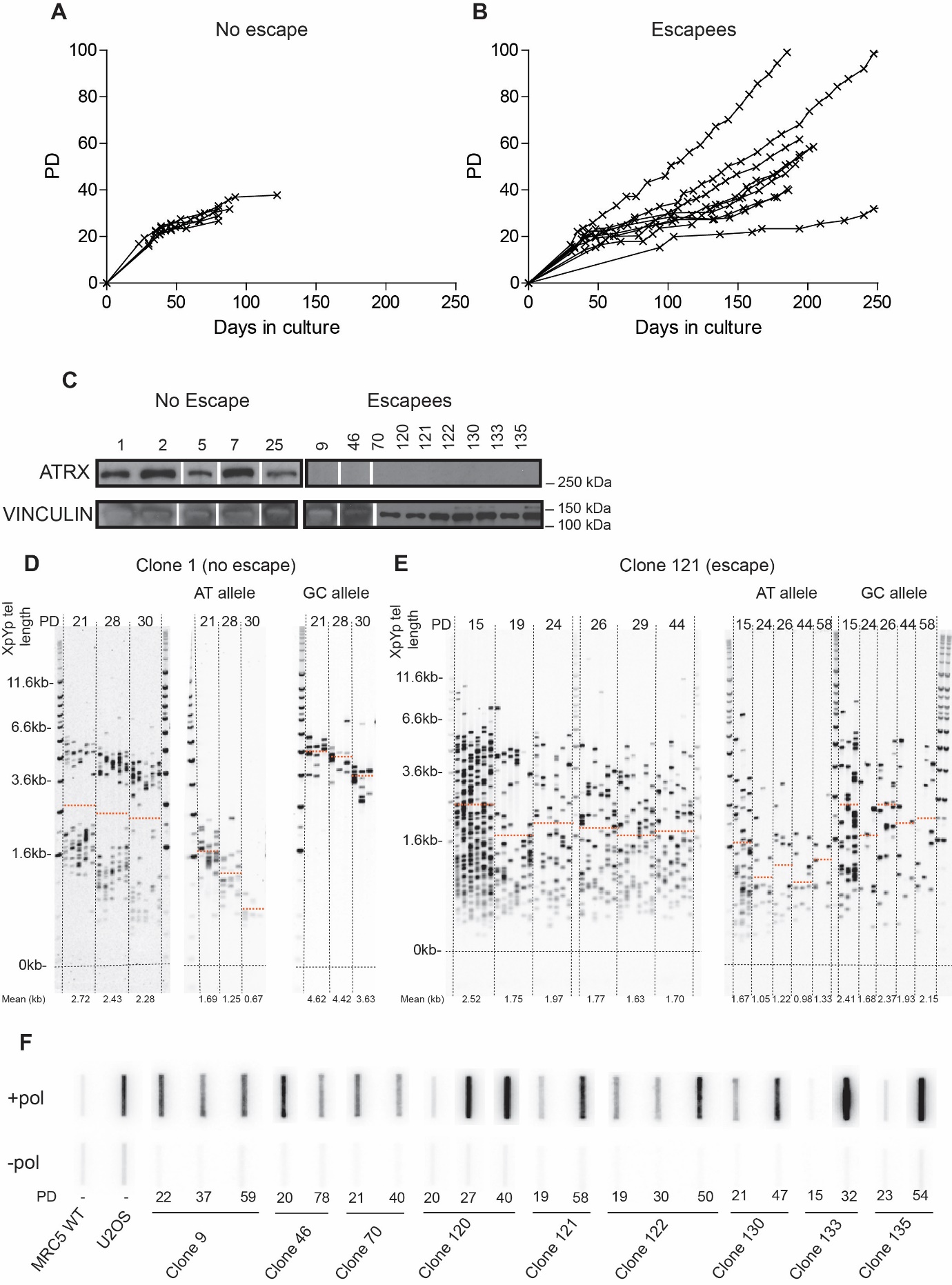


**S1 Fig: ALT activation and maintenance in the absence of ATRX in MRC5 primary fibroblasts undergoing a telomere-driven “crisis”.** Growth curves displaying population doublings (PDs) against days in culture for (A) MRC5^HPVE6E7^ clones (n = 5) that failed to escape crisis and (B) MRC5^HPVE6E7^ clones (n = 9) that successfully escaped crisis. (C) Western blots displaying ATRX protein expression in “no escape” and “escape” clones with Vinculin expression used as loading control. STELA profiles (overall and GC or AT allele-specific) at the XpYp chromosome end for clone 1 (D) that failed to escape crisis and clone 121 (E) that successfully escaped crisis; with the PD points stated across the top and the mean of the telomere length distributions detailed across the bottom, with the mean also represented as orange dotted lines on the blot. (F) C-circle assay slot blots of the with (+ pol) and without (- pol) φ29 DNA polymerase samples with the PD and clone number stated across the bottom.
