# Supplementary material for: ATRX modulates the escape from a telomere crisis
Source: PLoS Genet. 2022 Nov 9;18(11):e1010485. doi: 10.1371/journal.pgen.1010485 (PMC9678338; doi:10.1371/journal.pgen.1010485)
Supplement: S2 Fig — Examples of three HCA2HPVE6E7 clones that presented a mutated ATRX gene upon screening that were subsequently analysed by sequencing. In yellow is indicated the CRISPR target site as well as a Sml1 restriction site; dashes represent deletions. (DOCX) [file pgen.1010485.s002.docx]

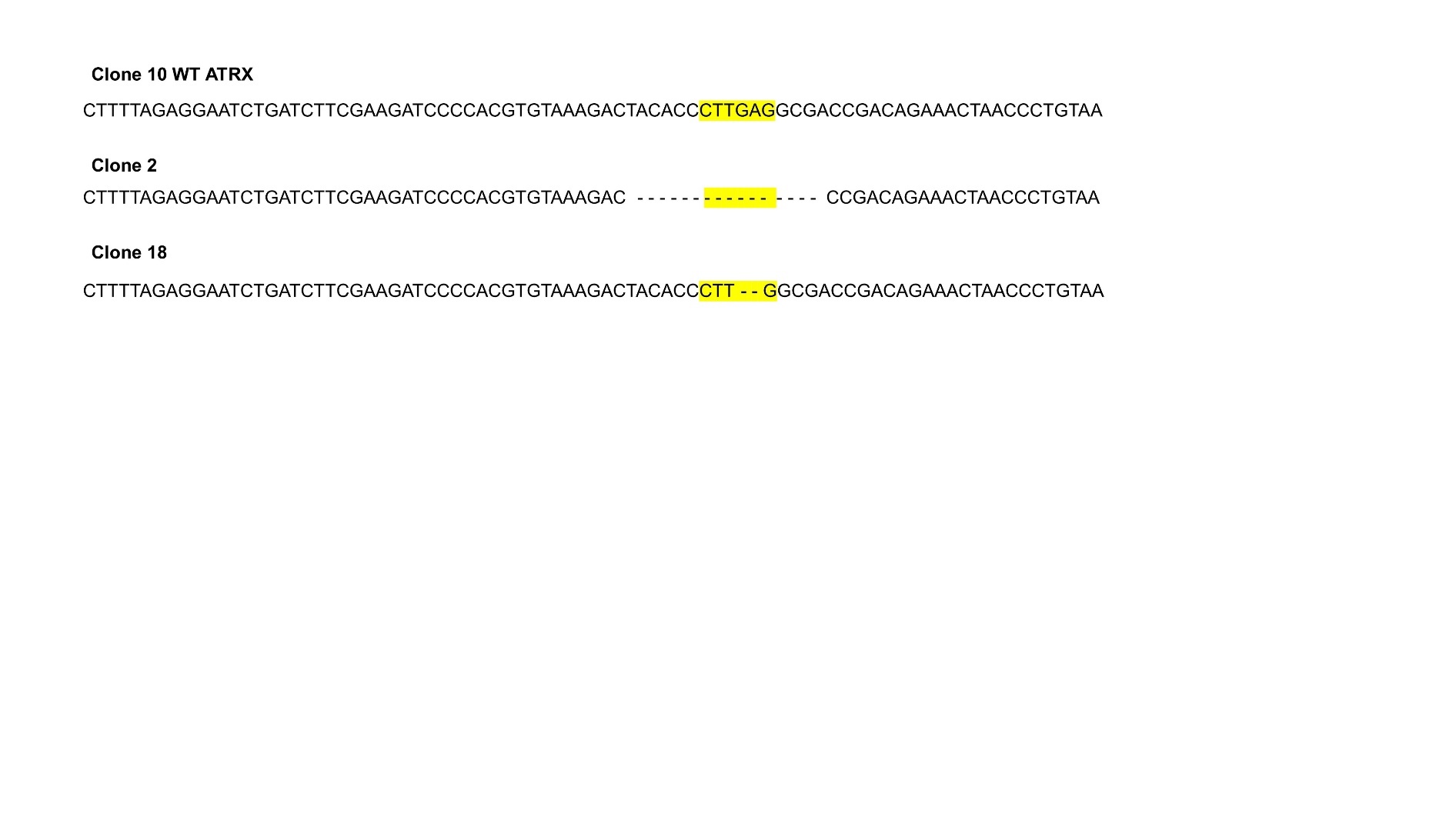


**S2 Fig: Sequence verification of selected clones exposed to ATRX CRISPR.** Examples of three HCA2^HPVE6E7^ clones that presented a mutated ATRX gene upon screening that were subsequently analysed by sequencing. In yellow is indicated the CRISPR target site as well as a Sml1 restriction site; dashes represent deletions.
