# Supplementary material for: ATRX modulates the escape from a telomere crisis
Source: PLoS Genet. 2022 Nov 9;18(11):e1010485. doi: 10.1371/journal.pgen.1010485 (PMC9678338; doi:10.1371/journal.pgen.1010485)
Supplement: S3 Fig — Growth curves displaying population doublings (PDs) against days in culture of the 6 HCA2HPVE6E7 single cell clones. (DOCX) [file pgen.1010485.s003.docx]

**
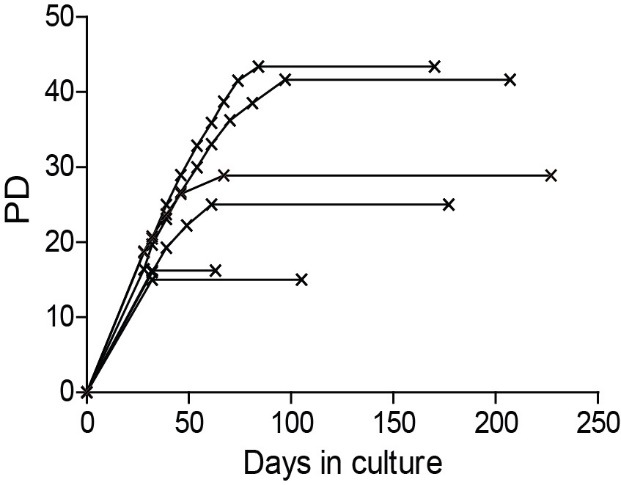
**

**S3 Fig: Induction of a telomere-driven crisis upon transfection of E6E7 viral oncoproteins in fibroblasts WT for ATRX.** Growth curves displaying population doublings (PDs) against days in culture of the 6 HCA2^HPVE6E7^ single cell clones.
