# Supplementary material for: ATRX modulates the escape from a telomere crisis
Source: PLoS Genet. 2022 Nov 9;18(11):e1010485. doi: 10.1371/journal.pgen.1010485 (PMC9678338; doi:10.1371/journal.pgen.1010485)
Supplement: S4 Fig — STELA profiles at the XpYp and 17p chromosome ends in HCA2HPVE6E7 cells for (A) clones 1 and 4 used as controls and (B) clones 2 and 44 that failed to escape crisis; with the PD points stated across the top and the mean of the telomere length distributions detailed across the bottom, with the mean also represented as orange dotted lines on the blot. (DOCX) [file pgen.1010485.s004.docx]

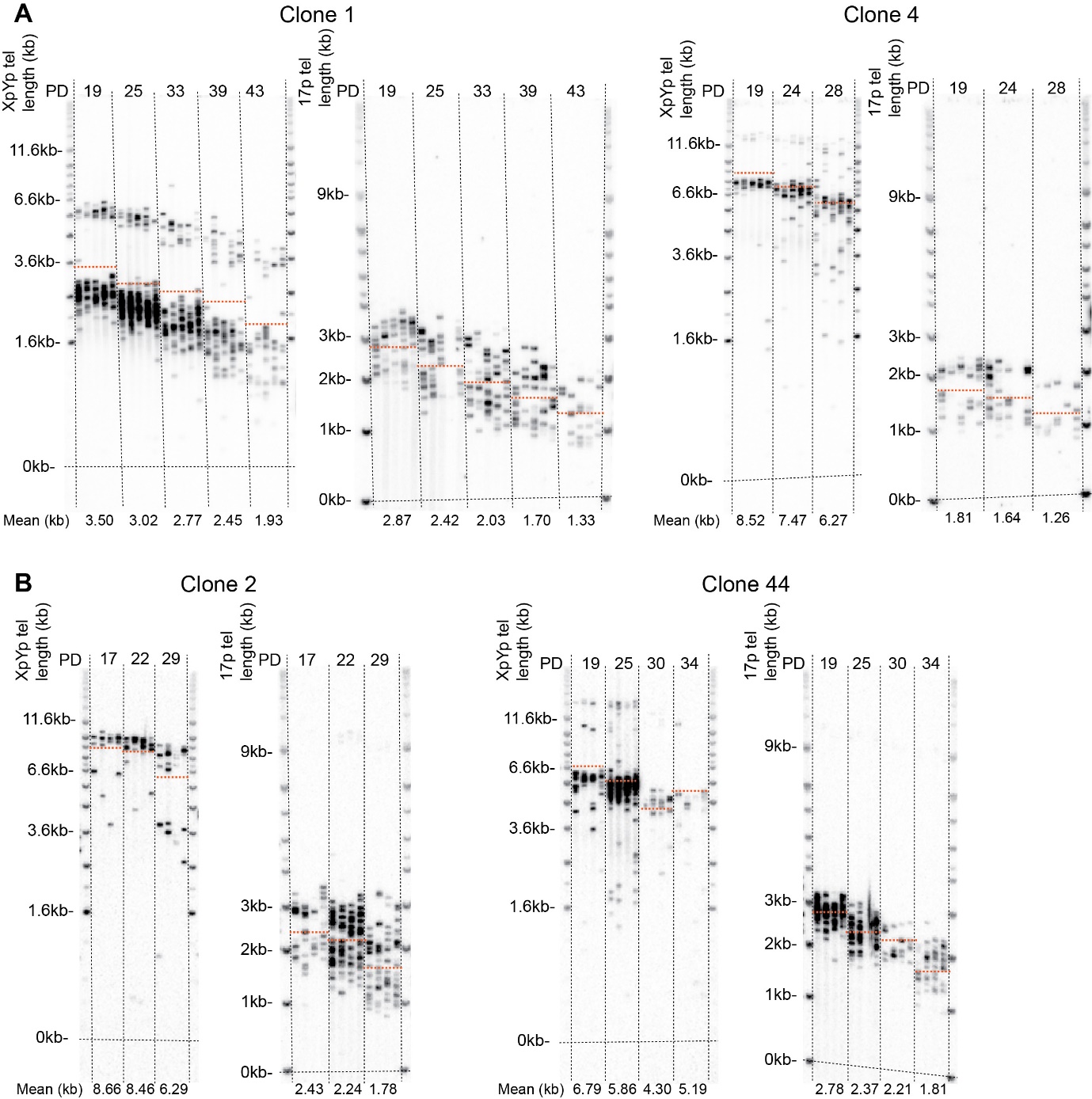


**S4 Fig: Homogeneous telomere length distributions in control and no escape clones**. STELA profiles at the XpYp and 17p chromosome ends in HCA2^HPVE6E7^ cells for (A) clones 1 and 4 used as controls and (B) clones 2 and 44 that failed to escape crisis; with the PD points stated across the top and the mean of the telomere length distributions detailed across the bottom, with the mean also represented as orange dotted lines on the blot.
