# Supplementary material for: ATRX modulates the escape from a telomere crisis
Source: PLoS Genet. 2022 Nov 9;18(11):e1010485. doi: 10.1371/journal.pgen.1010485 (PMC9678338; doi:10.1371/journal.pgen.1010485)
Supplement: S5 Fig — STELA profiles for (A) HCA2HPVE6E7 ATRX-/- clones 18 and 21 at the XpYp and 17p chromosome end; and (B) MRC5HPVE6E7 ATRX-/- clones 9 and 46 at the XpYp chromosome end for the combined alleles as well as for specific (GC or AT as indicated above) alleles; with the PD points stated across the top and the mean and standard deviation of the telomere length distributions detailed across the bottom, with the mean also represented as orange dotted lines on the blot. (DOCX) [file pgen.1010485.s005.docx]

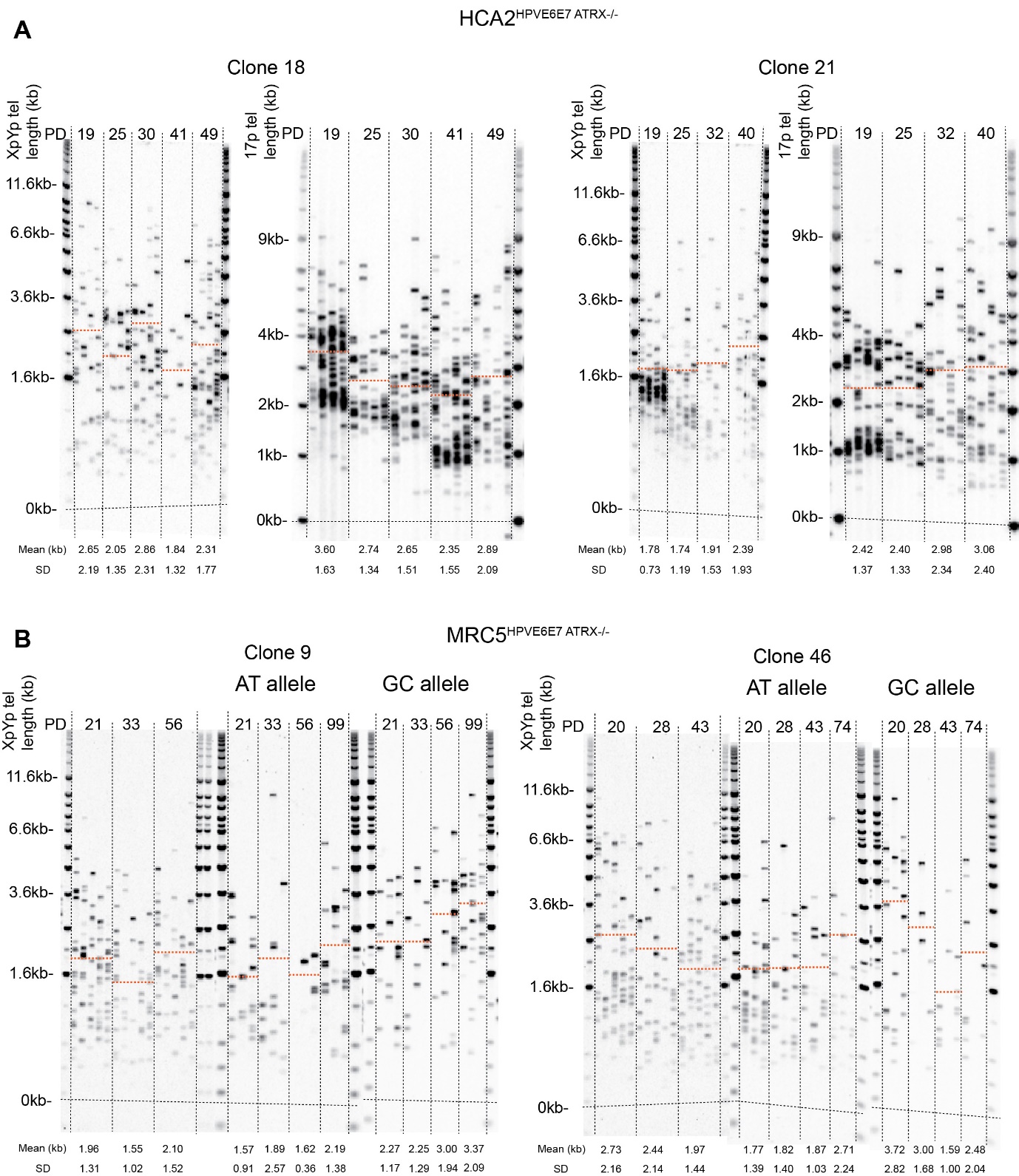


**S5 Fig: Heterogeneous telomere length distributions upon loss of ATRX and escape from crisis**. STELA profiles for (A) HCA2^HPVE6E7 ATRX-/-^ clones 18 and 21 at the XpYp and 17p chromosome end; and (B) MRC5^HPVE6E7 ATRX-/-^ clones 9 and 46 at the XpYp chromosome end for the combined alleles as well as for specific (GC or AT as indicated above) alleles; with the PD points stated across the top and the mean and standard deviation of the telomere length distributions detailed across the bottom, with the mean also represented as orange dotted lines on the blot.
