# Supplementary material for: ATRX modulates the escape from a telomere crisis
Source: PLoS Genet. 2022 Nov 9;18(11):e1010485. doi: 10.1371/journal.pgen.1010485 (PMC9678338; doi:10.1371/journal.pgen.1010485)
Supplement: S6 Fig — A) Example of fusion profiles for clone 1 (no escape) and clone 21 (escape) across multiple PD points (detailed across the top) and U2OS as an ALT-positive control. Blots were serially Southern hybridised with the telomere-adjacent DNA probes indicated on the left and the number of unique fusions stated below the blots. B) Bar charts depicting the number of XpYp (purple), 17p (white), 21q (grey) and total (black) fusion events as escapees are transiting through crisis and immortalising. (Number of diploid genome equivalents analysed = 2 x 10^4^). (DOCX) [file pgen.1010485.s006.docx]

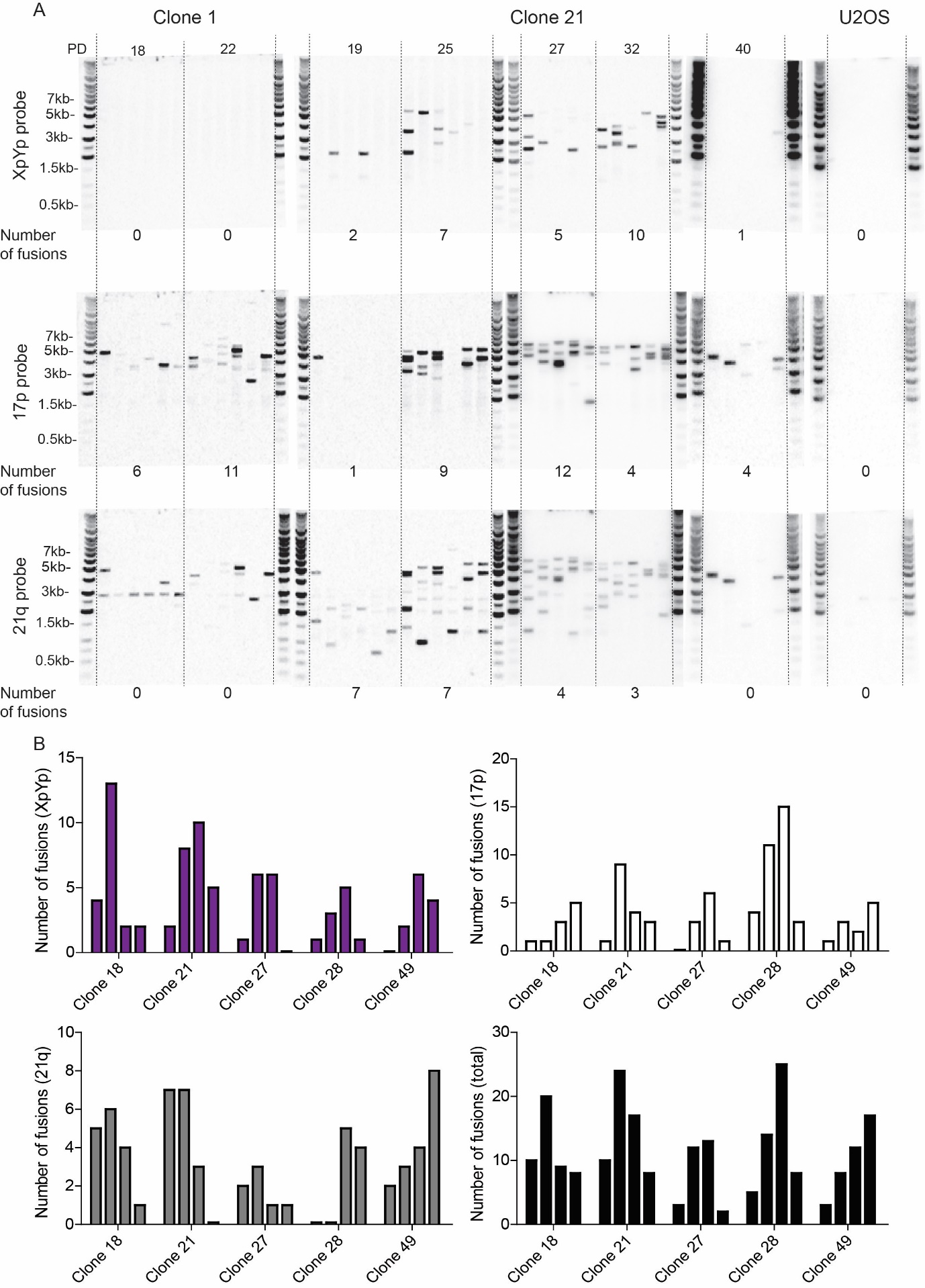


**S6 fig**: Fusion profiles reveal an increase of end-to-end fusions in escapees during crisis. A) Example of fusion profiles for clone 1 (no escape) and clone 21 (escape) across multiple PD points (detailed across the top) and U2OS as an ALT-positive control. Blots were serially Southern hybridised with the telomere-adjacent DNA probes indicated on the left and the number of unique fusions stated below the blots. B) Bar charts depicting the number of XpYp (purple), 17p (white), 21q (grey) and total (black) fusion events as escapees are transiting through crisis and immortalising. (Number of diploid genome equivalents analysed = 2 x 10^4^).
