# Supplementary material for: ATRX modulates the escape from a telomere crisis
Source: PLoS Genet. 2022 Nov 9;18(11):e1010485. doi: 10.1371/journal.pgen.1010485 (PMC9678338; doi:10.1371/journal.pgen.1010485)
Supplement: S7 Fig — The PD and clone number are stated across the bottom. (DOCX) [file pgen.1010485.s007.docx]

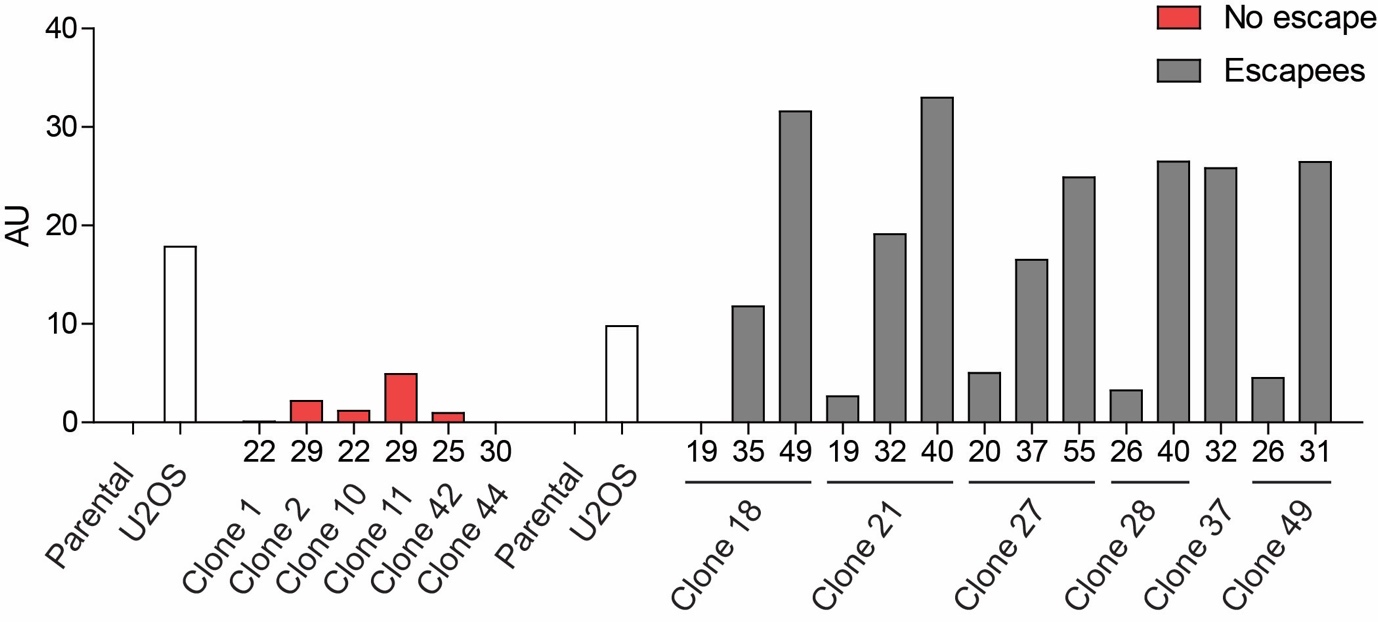


**S7 Fig:** Quantification of the C-circle slot blot intensity by subtracting the background (-pol) to the +pol sample and normalised to the HCA2^HPVE6E7;ATRX-/o^ fibroblasts parental cell line expressed in arbitrary unit (AU). The PD and clone number are stated across the bottom.
