# Supplementary material for: ATRX modulates the escape from a telomere crisis
Source: PLoS Genet. 2022 Nov 9;18(11):e1010485. doi: 10.1371/journal.pgen.1010485 (PMC9678338; doi:10.1371/journal.pgen.1010485)
Supplement: S8 Fig — TRAP assay results at the indicated PD points after the escape from crisis in A) HCA2HPVE6E7 ATRX-/- cells and B) MRC5HPVE6E7 ATRX-/- cells with the WT HCT116 cell line used as a positive control. (DOCX) [file pgen.1010485.s008.docx]

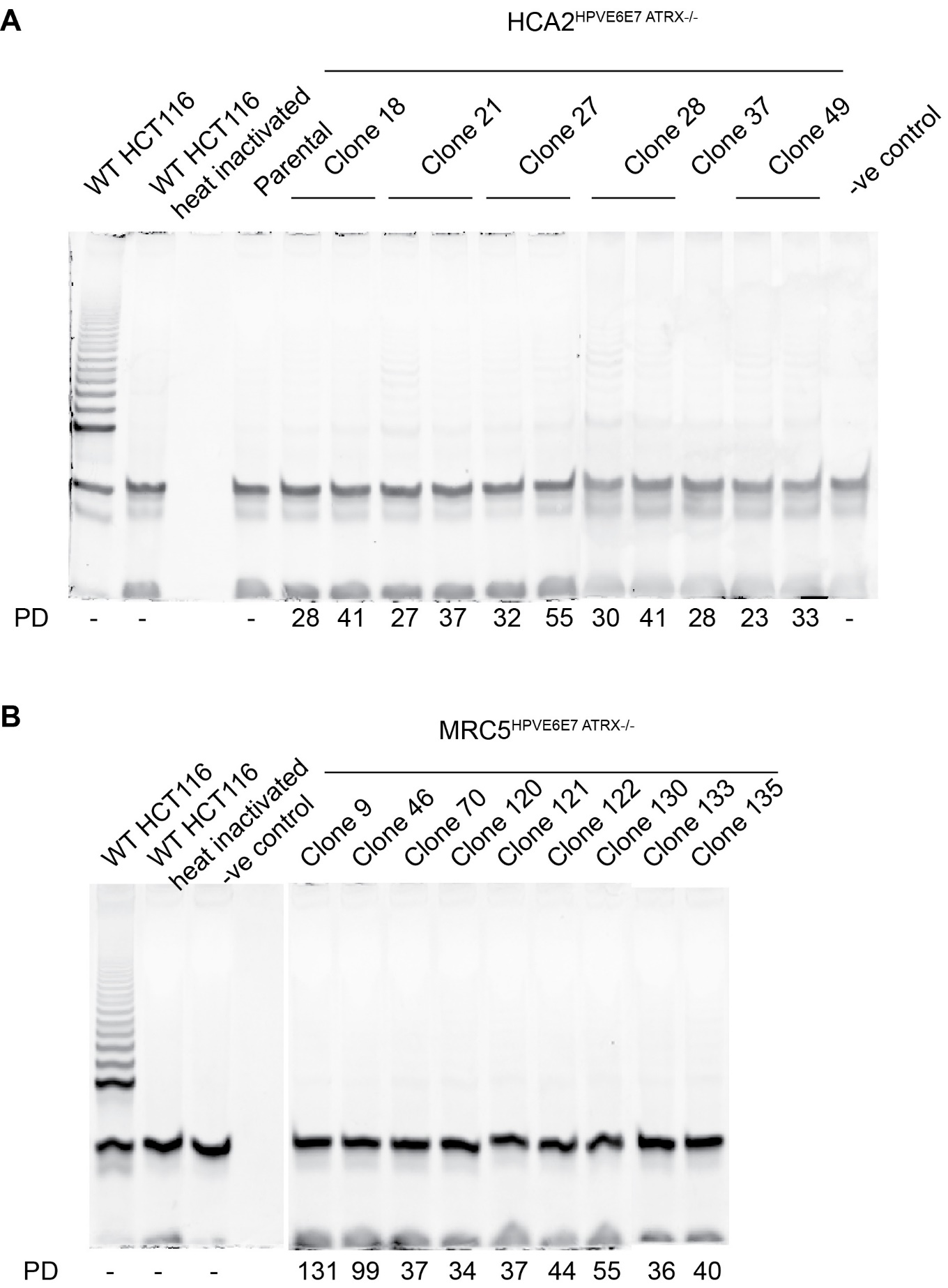


**S8 Fig: An absence of detectable telomerase activity in fibroblast cells that escaped crisis in the absence of ATRX.** TRAP assay results at the indicated PD points after the escape from crisis in A) HCA2^HPVE6E7 ATRX-/-^ cells and B) MRC5^HPVE6E7 ATRX-/-^ cells with the WT HCT116 cell line used as a positive control.
