# Supplementary material for: ATRX modulates the escape from a telomere crisis
Source: PLoS Genet. 2022 Nov 9;18(11):e1010485. doi: 10.1371/journal.pgen.1010485 (PMC9678338; doi:10.1371/journal.pgen.1010485)
Supplement: S9 Fig — 4X magnification of HCT116ATRX-/-:DN-hTERT cells (A) prior to crisis where they display small and healthy morphologies; and (B) large and multi-nucleated cells characteristic of cells undergoing crisis. (DOCX) [file pgen.1010485.s009.docx]

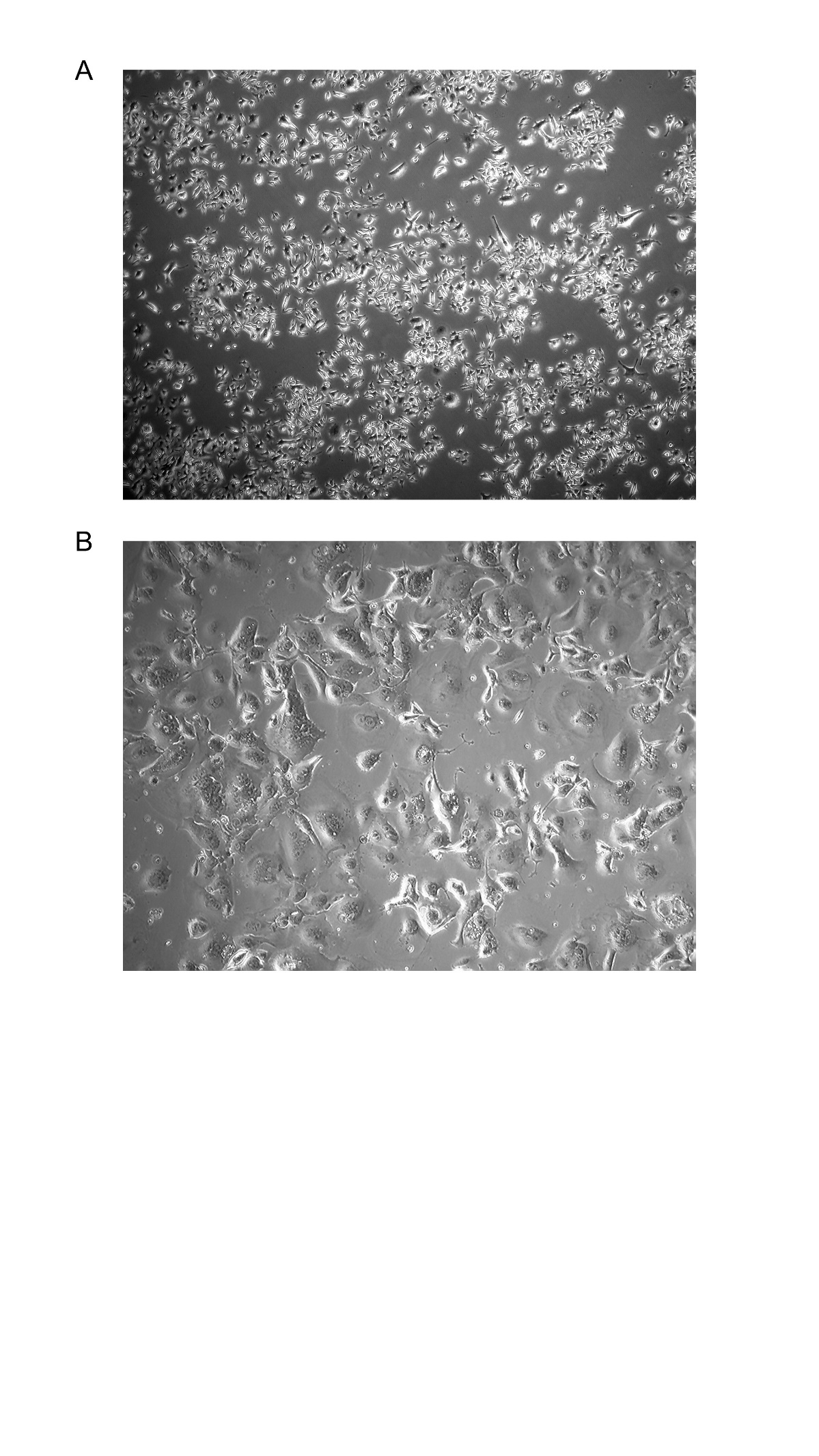


**S9 Fig: Crisis induces visible phenotypic changes to cells.** 4X magnification of HCT116^ATRX-/-:DN-hTERT^ cells (A) prior to crisis where they display small and healthy morphologies; and (B) large and multi-nucleated cells characteristic of cells undergoing crisis.
