# Supplementary material for: ATRX modulates the escape from a telomere crisis
Source: PLoS Genet. 2022 Nov 9;18(11):e1010485. doi: 10.1371/journal.pgen.1010485 (PMC9678338; doi:10.1371/journal.pgen.1010485)
Supplement: S10 Fig — Growth curve displaying PDs against days in culture for the puromycin control clones (n = 12) that were transfected with a puromycin selection gene to query the effects of a viral integration on HCT116ATRX-/- cells survival. (DOCX) [file pgen.1010485.s010.docx]

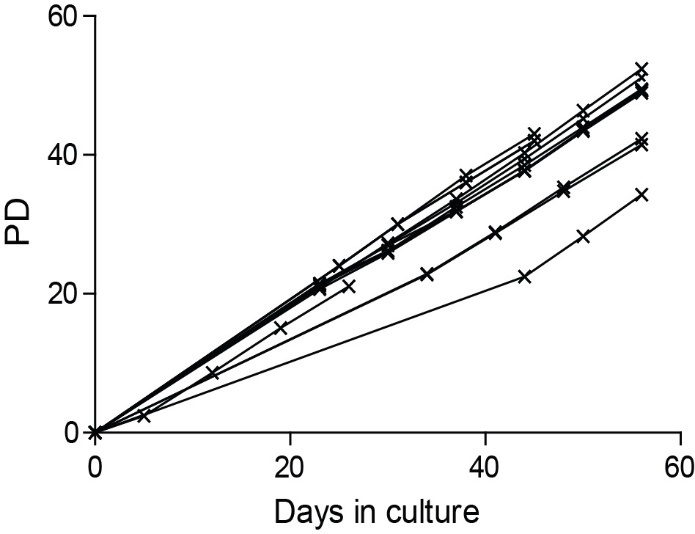


**S10 Fig: Cell growth of puromycin control HCT116^ATRX-/-^ clones.** Growth curve displaying PDs against days in culture for the puromycin control clones (n = 12) that were transfected with a puromycin selection gene to query the effects of a viral integration on HCT116^ATRX-/-^ cells survival.
