# Supplementary material for: ATRX modulates the escape from a telomere crisis
Source: PLoS Genet. 2022 Nov 9;18(11):e1010485. doi: 10.1371/journal.pgen.1010485 (PMC9678338; doi:10.1371/journal.pgen.1010485)
Supplement: S11 Fig — STELA profiles at the XpYp and 17p chromosome ends for HCT116ATRX-/-:DN-hTERT clone 147 that underwent telomere elongation at the XpYp chromosome end, but not at 17p, despite achieving replicative immortality. PD points are detailed across the top and the overall mean telomere length in black (represented by orange dotted lines on the blot) together with the estimated allelic telomere length distributions (red and green) across the bottom, also represented as dotted lines on the blot. (DOCX) [file pgen.1010485.s011.docx]

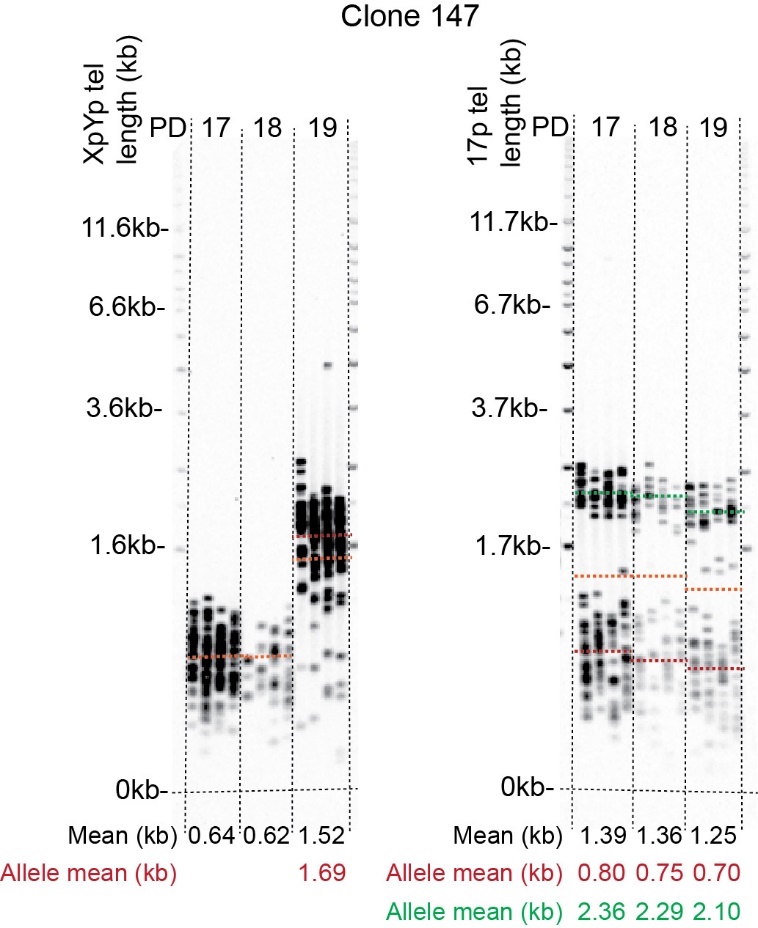


**S11 Fig: ALT-like elongation does not occur at all short telomeres.** STELA profiles at the XpYp and 17p chromosome ends for HCT116^ATRX-/-:DN-hTERT^ clone 147 that underwent telomere elongation at the XpYp chromosome end, but not at 17p, despite achieving replicative immortality. PD points are detailed across the top and the overall mean telomere length in black (represented by orange dotted lines on the blot) together with the estimated allelic telomere length distributions (red and green) across the bottom, also represented as dotted lines on the blot.
