# Supplementary material for: ATRX modulates the escape from a telomere crisis
Source: PLoS Genet. 2022 Nov 9;18(11):e1010485. doi: 10.1371/journal.pgen.1010485 (PMC9678338; doi:10.1371/journal.pgen.1010485)
Supplement: S12 Fig — 17p STELA of sub-clonal populations from Clone 3 (PD31) that successfully escaped crisis following ALT-like elongation of short telomeres. PD points from the point of single-cell cloning are indicated above, with the allele-specific mean telomere length detailed below. Sub-clone 2 highlighted in red died at PD17. Sub-clone 11 was serially passaged in culture, Δ telomere allelic telomere lengths are detailed below. (DOCX) [file pgen.1010485.s012.docx]

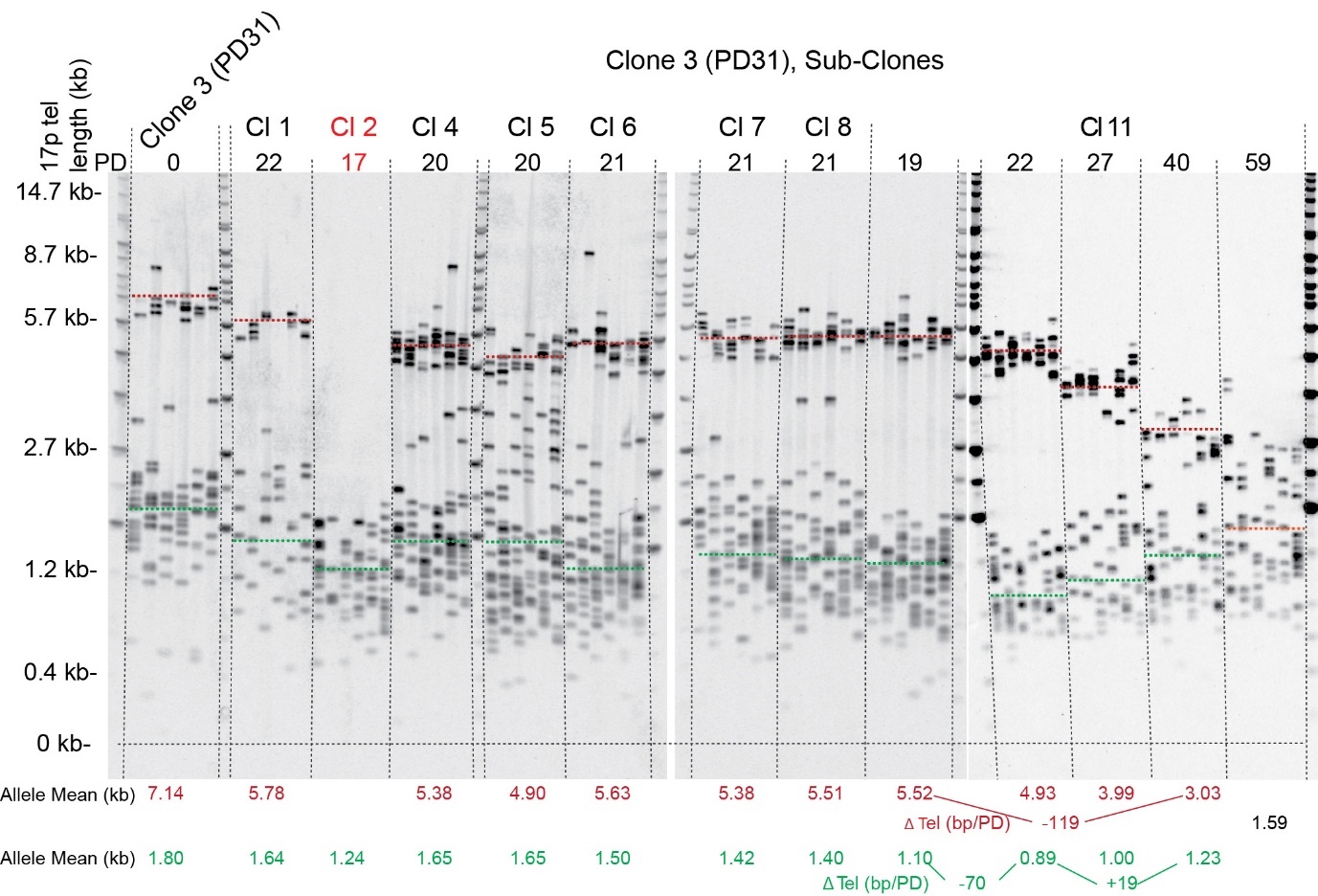


**S12 Fig: Telomeric elongation during crisis is allele specific.** 17p STELA of sub-clonal populations from Clone 3 (PD31) that successfully escaped crisis following ALT-like elongation of short telomeres. PD points from the point of single-cell cloning are indicated above, with the allele-specific mean telomere length detailed below. Sub-clone 2 highlighted in red died at PD17. Sub-clone 11 was serially passaged in culture, Δ telomere allelic telomere lengths are detailed below.
