# Supplementary material for: ATRX modulates the escape from a telomere crisis
Source: PLoS Genet. 2022 Nov 9;18(11):e1010485. doi: 10.1371/journal.pgen.1010485 (PMC9678338; doi:10.1371/journal.pgen.1010485)
Supplement: S13 Fig — STELA profiles at the (A) 17p and (B) XpYp chromosome ends with the PD stated across the top and the overall mean telomere length in black (represented as orange dotted lines on the blot), the longer allele in green, the shorter allele in red across the bottom also represented as dotted lines on the blot. The rate of erosion is represented by ΔTel in bp/PD. (C) Scatter plot depicting the mean telomere length of all available samples at the first sampling point and last sampling points for the 17p and XpYp chromosome ends with the p value stated above derived from a Mann-Whitney test (p-value < 0.05, highlighted in red). (DOCX) [file pgen.1010485.s013.docx]

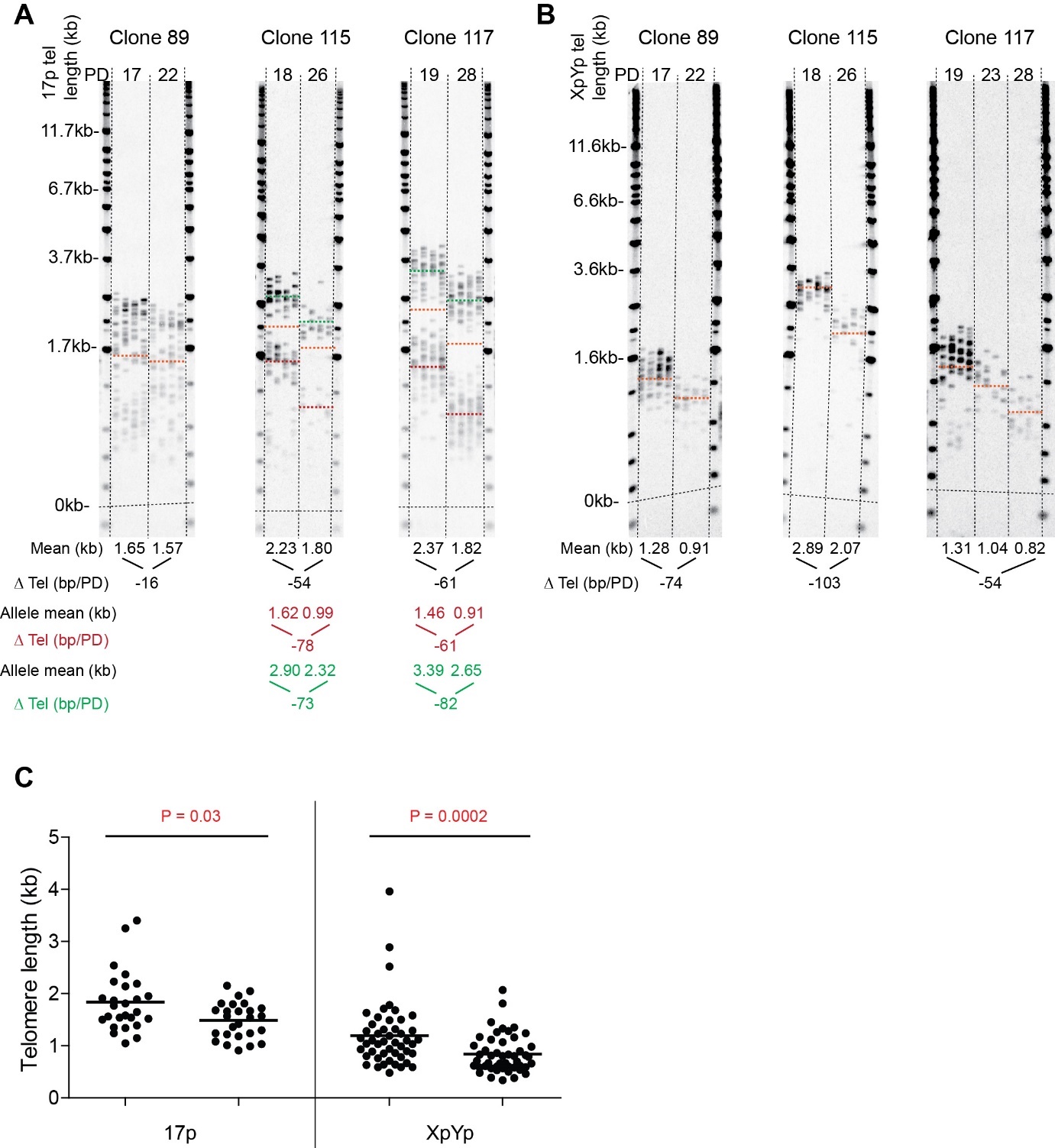


**S13 Fig: Telomere erosion following the expression of DN-hTERT in HCT116^ATRX-/-:DN-hTERT^ clones.** STELA profiles at the (A) 17p and (B) XpYp chromosome ends with the PD stated across the top and the overall mean telomere length in black (represented as orange dotted lines on the blot), the longer allele in green, the shorter allele in red across the bottom also represented as dotted lines on the blot. The rate of erosion is represented by ΔTel in bp/PD. (C) Scatter plot depicting the mean telomere length of all available samples at the first sampling point and last sampling points for the 17p and XpYp chromosome ends with the p value stated above derived from a Mann-Whitney test (p-value < 0.05, highlighted in red).
