# Supplementary material for: ATRX modulates the escape from a telomere crisis
Source: PLoS Genet. 2022 Nov 9;18(11):e1010485. doi: 10.1371/journal.pgen.1010485 (PMC9678338; doi:10.1371/journal.pgen.1010485)
Supplement: S14 Fig — STELA profiles at the (A) XpYp and (B) 17p chromosome ends for clones 92 and 111 which successfully escaped crisis activity with the PD stated across the top and the overall mean telomere length and standard deviation in black (represented by orange dotted lines on the blot), the longer allele in green, the shorter allele in red across the bottom also represented as dotted lines on the blot. (C) Scatter plot depicting the standard deviation for all available escaping clones before (black circles) and after crisis (black triangles) or after crisis only if no pre-crisis sample was available (red triangles) at the XpYp and 17p chromosome ends. The p-value stated above were derived from a student’s t-test (p-value < 0.05, highlighted in red). (DOCX) [file pgen.1010485.s014.docx]

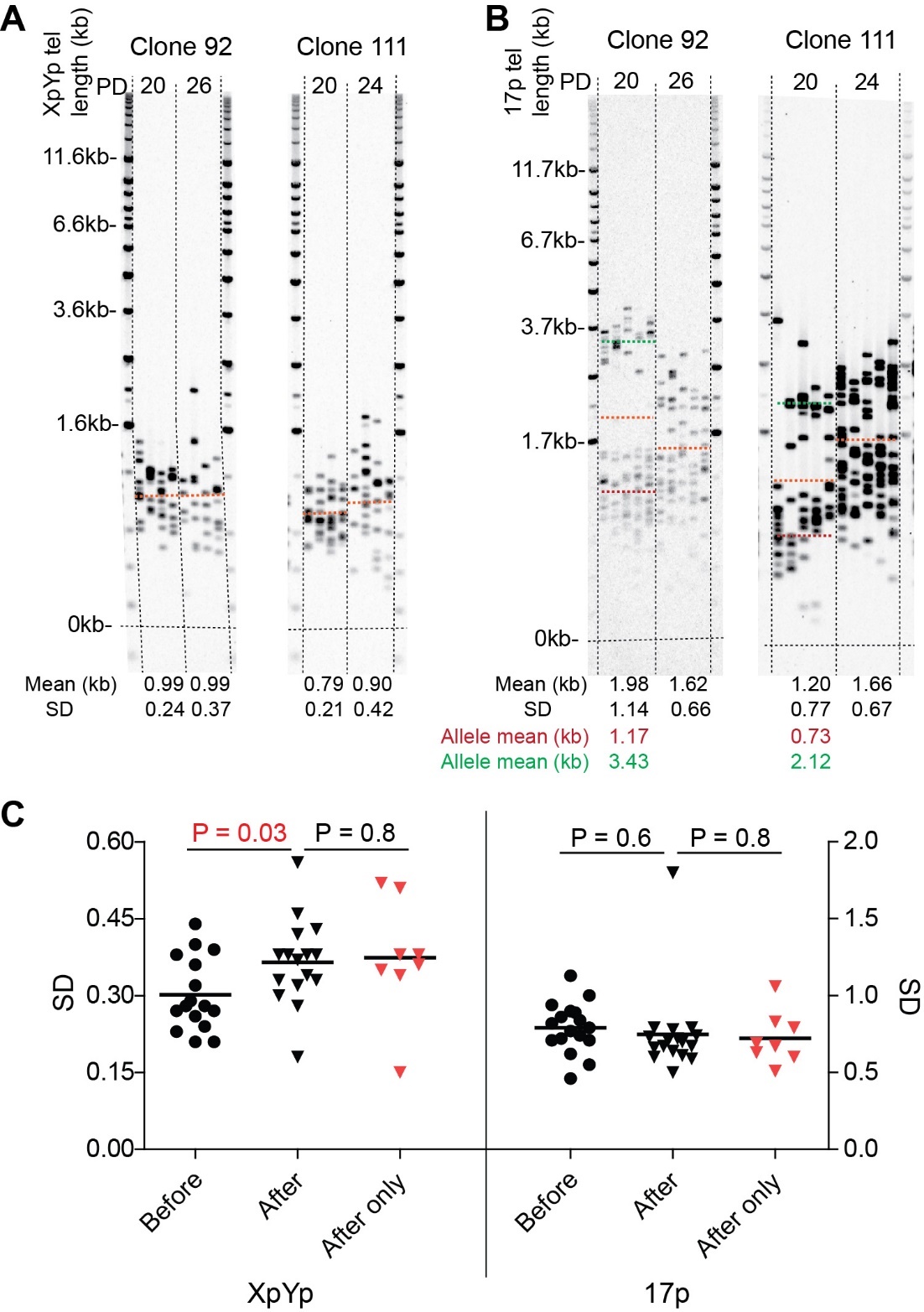


**S14 Fig: Increased heterogeneity of telomere length distributions upon the escape from crisis in HCT116^ATRX-/-:DN-hTERT^ clones.** STELA profiles at the (A) XpYp and (B) 17p chromosome ends for clones 92 and 111 which successfully escaped crisis activity with the PD stated across the top and the overall mean telomere length and standard deviation in black (represented by orange dotted lines on the blot), the longer allele in green, the shorter allele in red across the bottom also represented as dotted lines on the blot. (C) Scatter plot depicting the standard deviation for all available escaping clones before (black circles) and after crisis (black triangles) or after crisis only if no pre-crisis sample was available (red triangles) at the XpYp and 17p chromosome ends. The p-value stated above were derived from a student’s t-test (p-value < 0.05, highlighted in red).
