# Supplementary material for: ATRX modulates the escape from a telomere crisis
Source: PLoS Genet. 2022 Nov 9;18(11):e1010485. doi: 10.1371/journal.pgen.1010485 (PMC9678338; doi:10.1371/journal.pgen.1010485)
Supplement: S15 Fig — STELA profiles at the XpYp chromosome end for the HCT116ATRX-/- parental and the HCT116ATRX-/-:DN-hTERT ALT-like clones 2, 3 and 4 with the PD stated across the top and the mean telomere length in black (represented as orange dotted lines on the blot) and the allele that underwent telomere extension in red across the bottom also represented as dotted lines on the blot. The rate of erosion is represented by ΔTel in bp/PD. (DOCX) [file pgen.1010485.s015.docx]

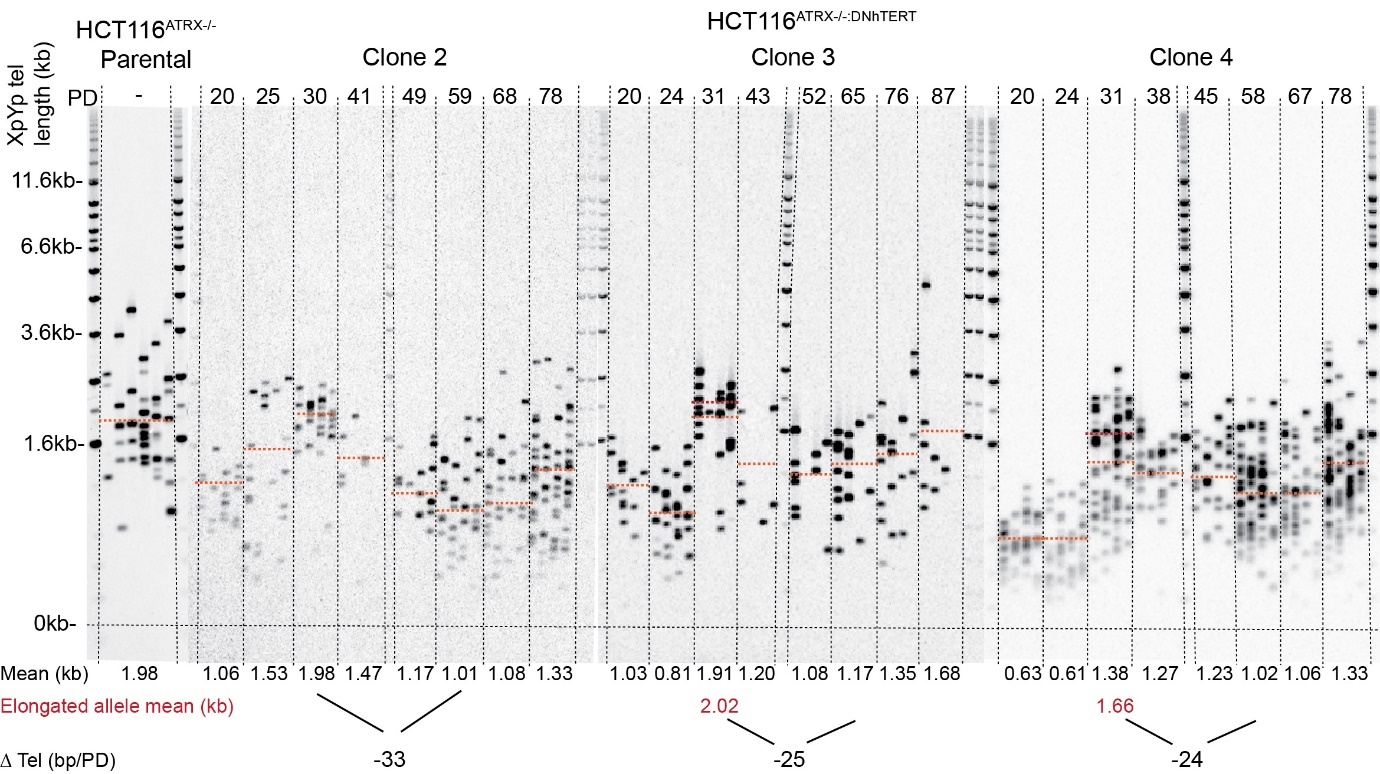


**S15 Fig: Consistent ALT-like telomere elongation at the XpYp chromosome end in HCT116^ATRX-/-:DN-hTERT^ clones following the escape from crisis**. STELA profiles at the XpYp chromosome end for the HCT116^ATRX-/-^ parental and the HCT116^ATRX-/-:DN-hTERT^ ALT-like clones 2, 3 and 4 with the PD stated across the top and the mean telomere length in black (represented as orange dotted lines on the blot) and the allele that underwent telomere extension in red across the bottom also represented as dotted lines on the blot. The rate of erosion is represented by ΔTel in bp/PD.
