# Supplementary material for: ATRX modulates the escape from a telomere crisis
Source: PLoS Genet. 2022 Nov 9;18(11):e1010485. doi: 10.1371/journal.pgen.1010485 (PMC9678338; doi:10.1371/journal.pgen.1010485)
Supplement: S16 Fig — (A) C-circle assay slot blots with (+ pol) and without (- pol) polymerase with the PD and clone number stated across the bottom. (B) Quantification of the slot blot intensity by subtracting the background (-pol) to the +pol sample and normalised to the HCT116ATRX-/- parental cell line expressed in arbitrary unit (AU) with the standard deviation used as error bars. The PD and clone number are stated across the bottom. (C) STELA profiles at the XpYp chromosome end with the PD across the top and the mean telomere length across the bottom also represented as orange dotted lines on the blot. (D) Telomerase activity quantification expressed in total product generated (TPG) with the standard deviation used as error bars where possible. (DOCX) [file pgen.1010485.s016.docx]

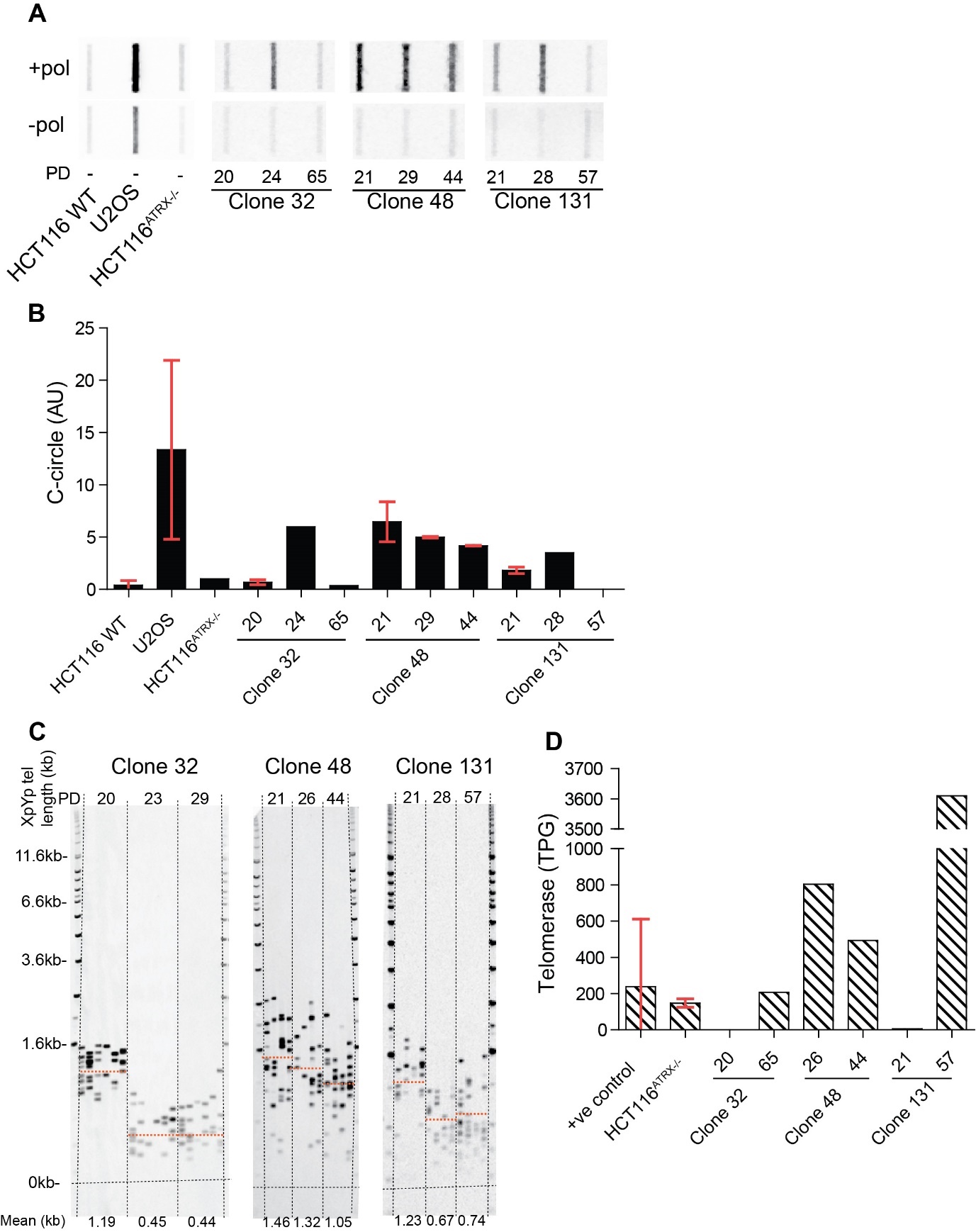


**S16 Fig: C-circles detected in the absence of telomeric elongation in HCT116^ATRX-/-:DN-hTERT^ clones that escaped crisis.** (A) C-circle assay slot blots with (+ pol) and without (- pol) polymerase with the PD and clone number stated across the bottom. (B) Quantification of the slot blot intensity by subtracting the background (-pol) to the +pol sample and normalised to the HCT116^ATRX-/-^ parental cell line expressed in arbitrary unit (AU) with the standard deviation used as error bars. The PD and clone number are stated across the bottom. (C) STELA profiles at the XpYp chromosome end with the PD across the top and the mean telomere length across the bottom also represented as orange dotted lines on the blot. (D) Telomerase activity quantification expressed in total product generated (TPG) with the standard deviation used as error bars where possible.
