# Supplementary material for: ATRX modulates the escape from a telomere crisis
Source: PLoS Genet. 2022 Nov 9;18(11):e1010485. doi: 10.1371/journal.pgen.1010485 (PMC9678338; doi:10.1371/journal.pgen.1010485)
Supplement: S17 Fig — STELA profiles at (A) XpYp and (B) 17p chromosome ends for HCT116ATRX-/-:DN-hTERT clones 108 and 132, which underwent an ALT-like elongation at XpYp, but failed to escape crisis. PD is detailed across the top; the mean telomere length in black (represented as orange dotted lines on the blot), the shorter allele prior to crisis in red and the longer allele prior to crisis in green across the bottom also represented as dotted lines on the blot. (C) C-circle assay slot blots with (+ pol) and without (- pol) polymerase samples with the PD and clone number stated across the bottom. (D) Quantification of C-circle intensity by subtracting the background (-pol) to the +pol sample normalised to the HCT116ATRX-/- parental expressed in arbitrary unit (AU) with the standard deviation used as error bars. The PD and clone number is stated across the bottom. (DOCX) [file pgen.1010485.s017.docx]

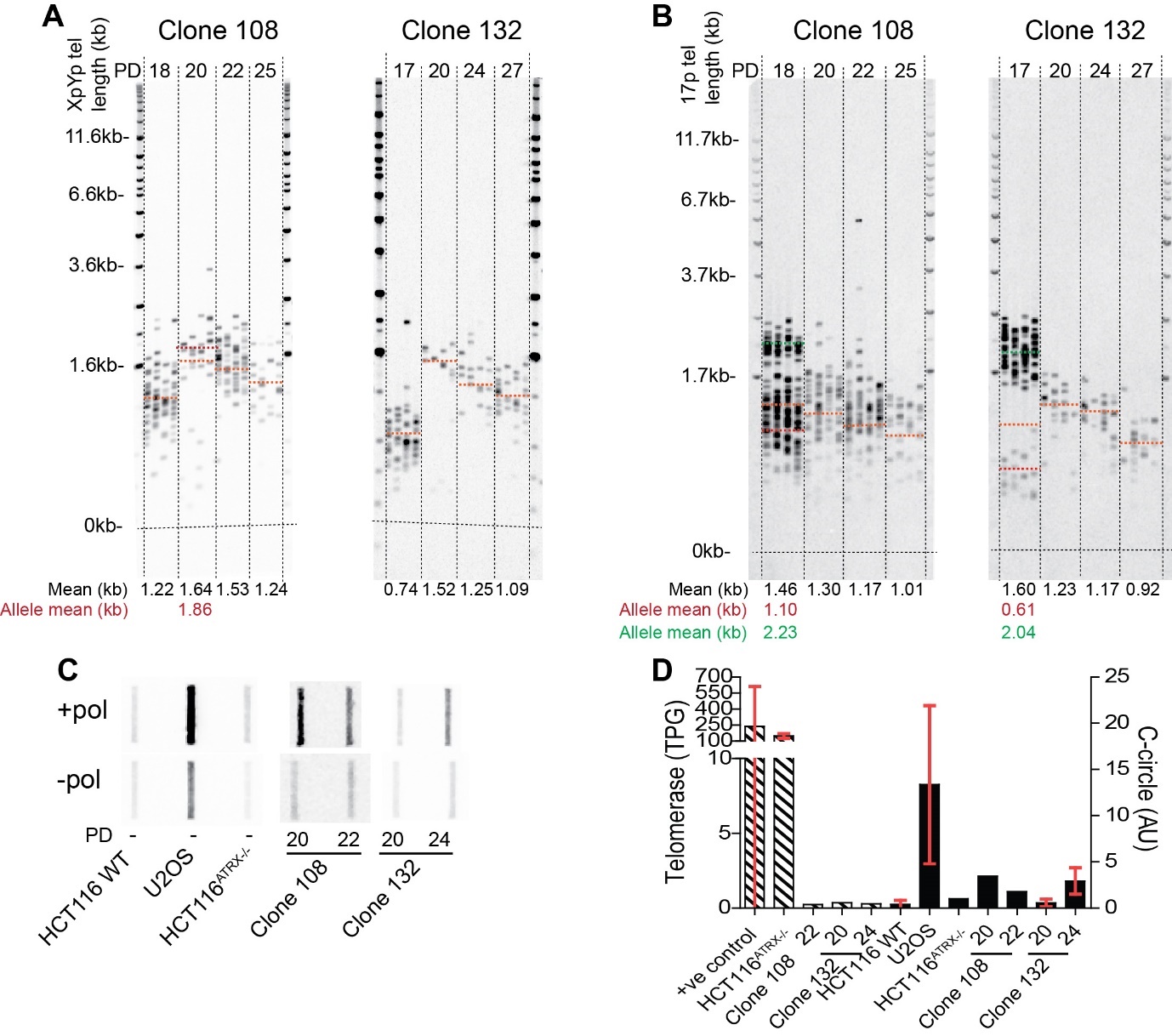


**S17 Fig: ALT-like activity** **does not** **always confer replicative immortality.** STELA profiles at (A) XpYp and (B) 17p chromosome ends for HCT116^ATRX-/-:DN-hTERT^ clones 108 and 132, which underwent an ALT-like elongation at XpYp, but failed to escape crisis. PD is detailed across the top; the mean telomere length in black (represented as orange dotted lines on the blot), the shorter allele prior to crisis in red and the longer allele prior to crisis in green across the bottom also represented as dotted lines on the blot. (C) C-circle assay slot blots with (+ pol) and without (- pol) polymerase samples with the PD and clone number stated across the bottom. (D) Quantification of C-circle intensity by subtracting the background (-pol) to the +pol sample normalised to the HCT116^ATRX-/-^ parental expressed in arbitrary unit (AU) with the standard deviation used as error bars. The PD and clone number is stated across the bottom.
