# Supplementary material for: ATRX modulates the escape from a telomere crisis
Source: PLoS Genet. 2022 Nov 9;18(11):e1010485. doi: 10.1371/journal.pgen.1010485 (PMC9678338; doi:10.1371/journal.pgen.1010485)
Supplement: S18 Fig — STELA profiles at the 5p, 7q, 9p and 8q chromosome ends for HCT116ATRX-/-:DN-hTERT (A) clone 2 and (B) clone 4 with the PD across the top and the mean telomere length across the bottom also represented as orange dotted lines on the blot. (C) Scatter plot displaying the elongated telomere distributions at the XpYp, 17p, 7q, 5p and 9p chromosome ends of the three clones that successfully escaped crisis using the ALT mechanism with standard deviation used as error bars. (D) Scatter plot displaying the insertion lengths (mean telomere length post-elongation minus the mean telomere length prior to crisis) at the XpYp, 17p, 7q, 5p and 9p chromosome ends. (DOCX) [file pgen.1010485.s018.docx]

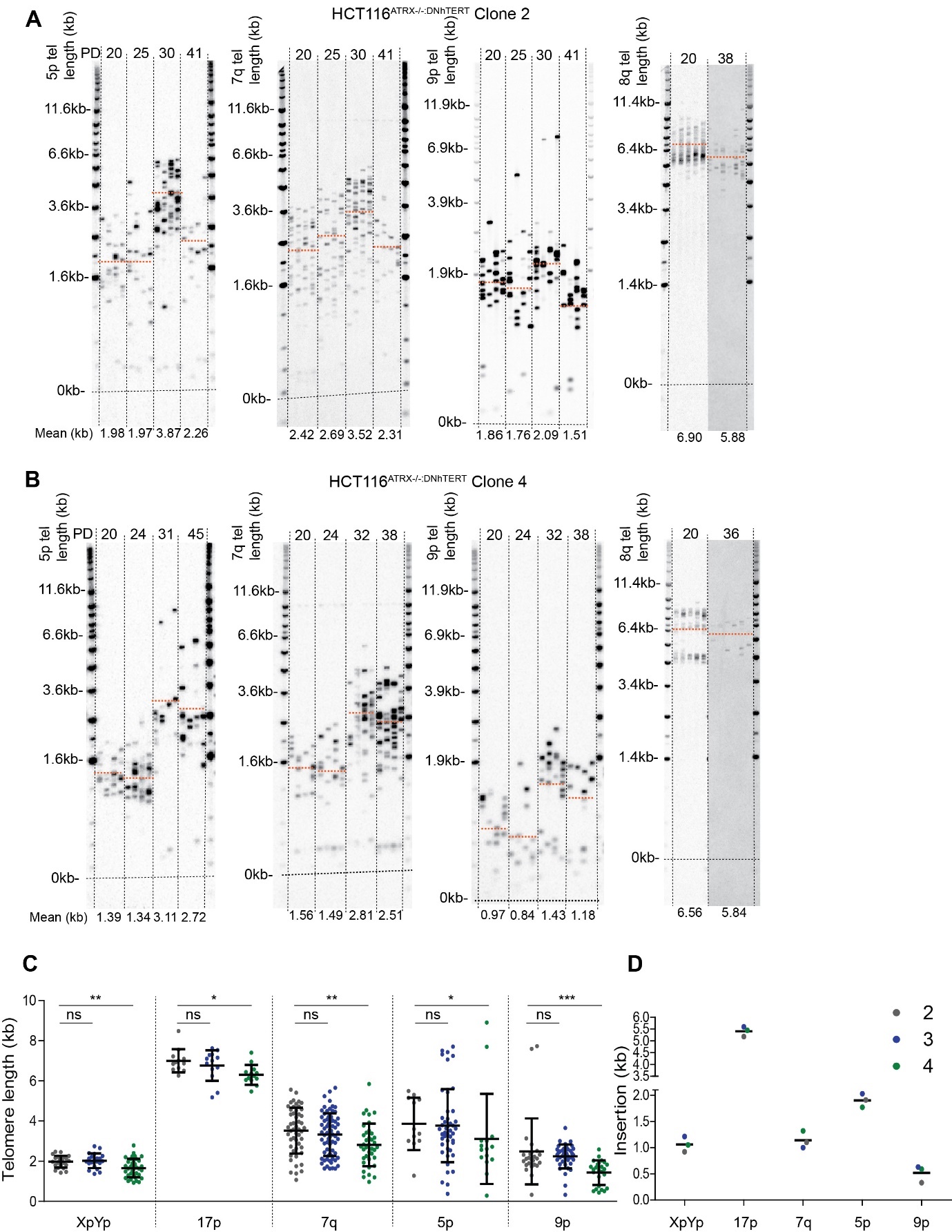


**S18 Fig: Consistent chromosome-specific elongation of telomeres.** STELA profiles at the 5p, 7q, 9p and 8q chromosome ends for HCT116^ATRX-/-:DN-hTERT^ (A) clone 2 and (B) clone 4 with the PD across the top and the mean telomere length across the bottom also represented as orange dotted lines on the blot. (C) Scatter plot displaying the elongated telomere distributions at the XpYp, 17p, 7q, 5p and 9p chromosome ends of the three clones that successfully escaped crisis using the ALT mechanism with standard deviation used as error bars. (D) Scatter plot displaying the insertion lengths (mean telomere length post-elongation minus the mean telomere length prior to crisis) at the XpYp, 17p, 7q, 5p and 9p chromosome ends.
