# Supplementary material for: ATRX modulates the escape from a telomere crisis
Source: PLoS Genet. 2022 Nov 9;18(11):e1010485. doi: 10.1371/journal.pgen.1010485 (PMC9678338; doi:10.1371/journal.pgen.1010485)
Supplement: S19 Fig — The total proportion of specific variant repeats combining all reads expressed in percentage (calculated by combining and averaging the number of a specific variant normalised to the telomere length) for the parental and the ALT clone with corresponding bar charts expressing the fold change in variant repeat proportion when comparing parental and ALT clone using a log scale (with the score of 1 representing no change) for (A) HCT116 model; (B) HCA2 model; and (C) U2OS. (DOCX) [file pgen.1010485.s019.docx]

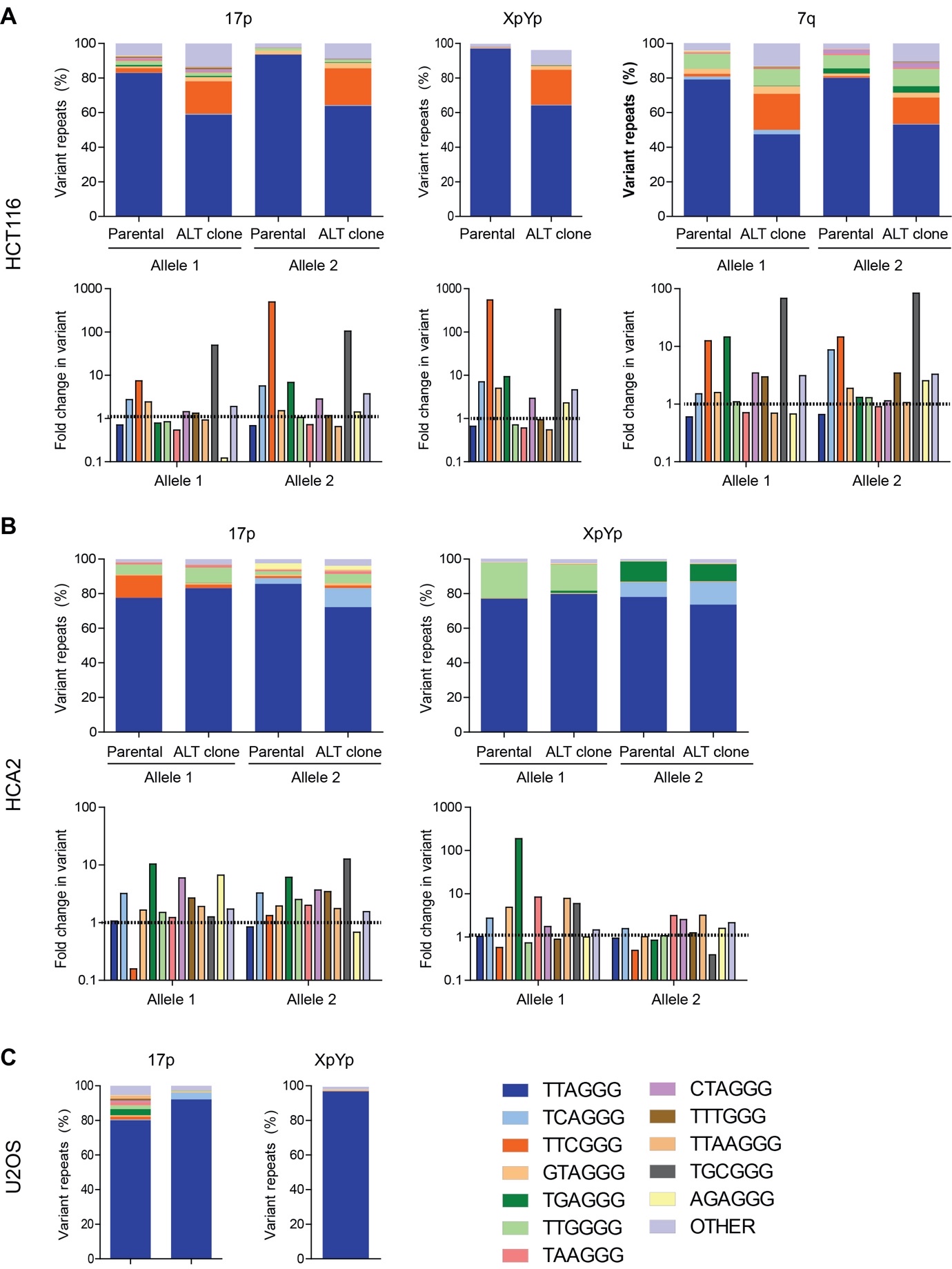


**S19 Fig: Altered telomere variant repeat patterns in ALT-positive clones.** The total proportion of specific variant repeats combining all reads expressed in percentage (calculated by combining and averaging the number of a specific variant normalised to the telomere length) for the parental and the ALT clone with corresponding bar charts expressing the fold change in variant repeat proportion when comparing parental and ALT clone using a log scale (with the score of 1 representing no change) for (A) HCT116 model; (B) HCA2 model; and (C) U2OS.
