# Supplementary material for: ATRX modulates the escape from a telomere crisis
Source: PLoS Genet. 2022 Nov 9;18(11):e1010485. doi: 10.1371/journal.pgen.1010485 (PMC9678338; doi:10.1371/journal.pgen.1010485)
Supplement: S20 Fig — Examples of telomere sequences obtained from PacBio sequencing of STELA amplicons obtained from HCT116ATRX-/o and HCA2HPVE6E7;ATRX-/o cells at the XpYp, 17p and 7q chromosome ends prior to crisis (Parental) and a single clone of each exhibiting characteristics of ALT post crisis, denoted as ALT from HCA2HPVE6E7;ATRX-/o fibroblasts and ALT-L (ALT-like) from HCT116ATRX-/o and ALT positive U2OS cells. Telomere and 6 nt variant repeat sequences are coded as follows: dark blue □ = TTAGGG; light blue □ = TCAGGG; dark orange □ = TTCGGG; beige □ = GTAGGG; dark green □ = TGAGGG; light green □ = TTGGGG; ochre □ = TAAGGG; lilac □ = CTAGGG; brown = □ TTTGGG; yellow = □ AGAGGG; white = □ TVRs ≶ 6 nt. (DOCX) [file pgen.1010485.s020.docx]

**HCT116**

XpYp telomere

Parental

TGG□□□□□□□□□□□□□□□□ □□□□□□□□□□□□□□□□□□□□□□□□□□□□□□□□□□□□□□□□□TT

TGG□□□□□□□□□□□□□□□□□□□□□□□□□□□□□□□□□□□□□□□□□□□□□□□□□□□□□□□□□□□□□□□□□□□□□□□□□□□□□□□□□□□□□□TT

TGG□□□□□□□□□□□□□□□□□□□□□□□□□□□□□□□□□□□□□□□□□□□□□□□□□□□□□□□□□□□□□□□□□□□□□□□□□□□□□□□□□□□□□□□□□□□□□□□□□□□□□□□□□□□□□□□□□□□□□□□□□□TT

TGG□□□□□□□□□□□□□□□□□□□□□□□□□□□□□□□□□□□□□□□□□□□□□□□□□□□□□□□□□□□□□□□□□□□□□□□□□□□□□□□□□□□□□□□□□□□□□□□□□□□□□□□□□□□□□□□□□□□□□□□□□□□□□□□□□□□□□□□□□□□□□□□□□TT

TGG□□□□□□□□□□□□□□□□□□□□□□□□ □□□□□□□□□□□□□□□□□□□□□□□□□□□□□□□□□□□□□□□□□□□□□□□□□□□□□□□□□□□□□□□□□□□□□□□□□□□□□□□□□□□□□□□□□□□□□□□□□□□□□□□□□□□□□□□□□□□□□□□□□□□□□□□□□□□□□□□□□□□□□□□□□□□□□□□□□□□□□□□□□□□□□□T

TGG□□□□□□□□□□□□□□□□□□□□□□□□□□□□□□□□□□□□□□□□□□□□□□□□□□□□□□□□□□□□□□□□□□□□□□□□□□□□□□□□□□□□□□□□□□□□□□□□□□□□□□□□□□□□□□□□□□□□□□□□□□□□□□□□□□□□□□□□□□□□□□□□□□□□□□□□□□□□□□□□□□□□□□□□□□□□□□□□□□□□□□□□□□□□□□□□□□□□□□□□□□□□□□□□□□□□□□□□□□□□□□□□□□□□□TT

TGG□□□□□□□□□□□□□□□□□□□□□□□□□□□□□□□□□□□□□□□□□□□□□□□□□□□□□□□□□□□□□□□□□□□□□□□□□□□□□□□□□□□□□□□□□□□□□□□□□□□□□□□□□□□□□□□□□□□□□□□□□□□□□□□□□□□□□□□□□□□□□□□□□□□□□□□□□□□□□□□□□□□□□□□□□□□□□□□□□□□□□□□□□□□□□□□□□□□□□□□□□□□□□□□□□□□□□□□□□□□□□□□□□□□□□□□□□□□□□□□□□□□□□□□□□□□□□□□TT

TGG□□□□□□□□□□□□□□□□□□□□□□□□□□□□□□□□□□□□□□□□□□□□□□□□□□□□□□□□□□□□□□□□□□□□□□□□□□□□□□□□□□□□□□□□□□□□□□□□□□□□□□□□□□□□□□□□□□□□□□□□□□□□□□□□□□□□□□□□□□□□□□□□□□□□□□□□□□□□□□□□□□□□□□□□□□□□□□□□□□□□□□□□□□□□□□□□□□□□□□□□□□□□□□□□□□□□□□□□□□□□□□□□□□□□□□□□□□□□□□□□□□□□□□□□□□□□□□□□□□□□□□□□□□□□□□□□□□□□□□□□□□□□□□□□□□□□□□□□□□□□□□□□□□□□□□□□□□□□□□□□□□□□□□□□□□□□□□□□□□□□□□□□□□□□□□□□□□□□□□□□□□□□□□□□□□□□□□□□□□□□□□□TT

ALT-like clone

TGG□□□□□□□□□□□□□□□□□□□□□□□□□□□□□□□□□□□□□□□□□□□□□□□□□□□□□□□□□□□□□□□TT

TGG□□□□□□□□□□□□□□□□□□□□□□□□□□□□□□□□□□□□□□□□□□□□□□□□□□□□□□□□□□□□□□□□□□□□□□□□□□□□□□□□□□□□□□□□□□□□□□□□□□□□□□□□□□□□□□□□□□□□□□□□□□□□□□□□□□□□□□□□□□□□□□□□□□□□□□□□□□□□□□□□□□□□□□□□□□□□□□□□□□□□□□□□□□□□□□□□□□□□□□□□□□□□□□□□□□□□□□□□□□□□□□□□□□□□□□□□□□□□□□□□□□□□□□□□□□□□□□□□□□□□□□□□□□□□□□□□□□□□□□□□□□□□□□□□□□□□□□□□□□□□□□□□□□□□□□□□□□□□□□□□□□□□□□T

TGG□□□□□□□□□□□□□□□□□□□□□□□□□□□□□□□□□□□□□□□□□□□□□□□□□□□□□□□□□□□□□□□TT

TGG□□□□□□□□□□□□□□□□□□□□□□□□□□□□□□□□□□□□□□□□□□□□□□□□□□□□□□□□□□□□□□□□□□□□□□□□□□□

TGG□□□□□□□□□□□□□□□□□□□□□□□□□□□□□□□□□□□□□□□□□□□□□□□□□□□□□□□□□□□□□□□□□□□□□□□□□□□□□□□□TT

TGG□□□□□□□□□□□□□□□□□□□□□□□□□□□□□□□□□□□□□□□□□□□□□□□□□□□□□□□□□□□□□□□□□□□□□□□□□□□□□□□□□□□TT

TGG□□□□□□□□□□□□□□□□□□□□□□□□□□□□□□□□□□□□□□□□□□□□□□□□□□□□□□□□□□□□□□□□□□□□□□□□□□□□□□□□□□□□□□□□□□TT

TGG□□□□□□□□□□□□□□□□□□□□□□□□□□□□□□□□□□□□□□□□□□□□□□□□□□□□□□□□□□□□□□□□□□□□□□□□□□□□□□□□□□□□□□□□□□□□□□□□□□TT

TGG□□□□□□□□□□□□□□□□□□□□□□□□□□□□□□□□□□□□□□□□□□□□□□□□□□□□□□□□□□□□□□□□□□□□□□□□□□□□□□□□□□□□□□□□□□□□□□□□□□□□□□□□□□□□□□TT

TGG□□□□□□□□□□□□□□□□□□□□□□□□□□□□□□□□□□□□□□□□□□□□□□□□□□□□□□□□□□□□□□□□□□□□□□□□□□□□□□□□□□□□□□□□□□□□□□□□□□□□□□□□□□□□□□□□□□□□□□TT

TGG□□□□□□□□□□□□□□□□□□□□□□□□□□□□□□□□□□□□□□□□□□□□□□□□□□□□□□□□□□□□□□□□□□□□□□□□□□□□□□□□□□□□□□□□□□□□□□□□□□□□□□□□□□□□□□□□□□□□□□□□□TT

TGG□□□□□□□□□□□□□□□□□□□□□□□□□□□□□□□□□□□□□□□□□□□□□□□□□□□□□□□□□□□□□□□□□□□□□□□□□□□□□□□□□□□□□□□□□□□□□□□□□□□□□□□□□□□□□□□□□□□□□□□□□□□□□□□T

TGG□□□□□□□□□□□□□□□□□□□□□□□□□□□□□□□□□□□□□□□□□□□□□□□□□□□□□□□□□□□□□□□□□□□□□□□□□□□□□□□□□□□□□□□□□□□□□□□□□□□□□□□□□□□□□□□□□□□□□□□□□□□□□□□□□□□□□□□□□□□□□TTA

TGG□□□□□□□□□□□□□□□□□□□□□□□□□□□□□□□□□□□□□□□□□□□□□□□□□□□□□□□□□□□□□□□□□□□□□□□□□□□□□□□□□□□□□□□□□□□□□□□□□□□□□□□□□□□□□□□□□□□□□□□□□□□□□□□□□□□□□□□□□□□□□□TT

TGG□□□□□□□□□□□□□□□□□□□□□□□□□□□□□□□□□□□□□□□□□□□□□□□□□□□□□□□□□□□□□□□□□□□□□□□□□□□□□□□□□□□□□□□□□□□□□□□□□□□□□□□□□□□□□□□□□□□□□□□□□□□□□□□□□□□□□□ □□□□□□□□□□□□□□□□□□□□□□□□□□□

TGG□□□□□□□□□□□□□□□□□□□□□□□□□□□□□□□□□□□□□□□□□□□□□□□□□□□□□□□□□□□□□□□□□□□□□□□□□□□□□□□□□□□□□□□□□□□□□□□□□□□□□□□□□□□□□□□□□□□□□□□□□□□□□□□□□□□□□□□□□□□□□□□□□□□□□□□□□□□□□□□□

TGG□□□□□□□□□□□□□□□□□□□□□□□□□□□□□□□□□□□□□□□□□□□□□□□□□□□□□□□□□□□□□□□□□□□□□□□□□□□□□□□□□□□□□□□□□□□□□□□□□□□□□□□□□□□□□□□□□□□□□□□□□□□□□□□□□□□□□□□□□□□□□□□□□□□□□□□□□□□□□□□□□□□□□□□□□□□□□T

TGG□□□□□□□□□□□□□□□□□□□□□□□□□□□□□□□□□□□□□□□□□□□□□□□□□□□□□□□□□□□□□□□□□□□□□□□□□□□□□□□□□□□□□□□□□□□□□□□□□□□□□□□□□□□□□□□□□□□□□□□□□□□□□□□□□□□□□□□□□□□□□□□□□□□□□□□□□□□□□□□□□□□□□□□□□□□□□□□□□□□□□□□□□□□□□□□□□□□□□□□□□□□TT

TGG□□□□□□□□□□□□□□□□□□□□□□□□□□□□□□□□□□□□□□□□□□□□□□□□□□□□□□□□□□□□□□□□□□□□□□□□□□□□□□□□□□□□□□□□□□□□□□□□□□□□□□□□□□□□□□□□□□□□□□□□□□□□□□□□□□□□□□□□□□□□□□□□□□□□□□□□□□□□□□□□□□□□□□□□□□□□□□□□□□□□□□□□□□□□□□□□□□□□□□□□□□□TT

TGG□□□□□□□□□□□□□□□□□□□□□□□□□□□□□□□□□□□□□□□□□□□□□□□□□□□□□□□□□□□□□□□□□□□□□□□□□□□□□□□□□□□□□□□□□□□□□□□□□□□□□□□□□□□□□□□□□□□□□□□□□□□□□□□□□□□□□□□□□□□□□□□□□□□□□□□□□□□□□□□□□□□□□□□□□□□□□□□□□□□□□□□□□□□□□□□□□□□□□□□□□□□□□□□□□□□□□□□□□□□□□□□□□□□□□□□□□□□□□□□□□□□□□□□□□□□□□□□□□□□□□TT

TGG□□□□□□□□□□□□□□□□□□□□□□□□□□□□□□□□□□□□□□□□□□□□□□□□□□□□□□□□□□□□□□□□□□□□□□□□□□□□□□□□□□□□□□□□□□□□□□□□□□□□□□□□□□□□□□□□□□□□□□□□□□□□□□□□□□□□□□□□□□□□□□□□□□□□□□□□□□□□□□□□□□□□□□□□□□□□□□□□□□□□□□□□□□□□□□□□□□□□□□□□□□□□□□□□□□□□□□□□□□□□□□□□□□□□□□□□□□□□□□□□□□□□□□□□□□□□□□□□□□□□TT

TGG□□□□□□□□□□□□□□□□□□□□□□□□□□□□□□□□□□□□□□□□□□□□□□□□□□□□□□□□□□□□□□□□□□□□□□□□□□□□□□□□□□□□□□□□□□□□□□□□□□□□□□□□□□□□□□□□□□□□□□□□□□□□□□□□□□□□□□□□□□□□□□□□□□□□□□□□□□□□□□□□□□□□□□□□□□□□□□□□□□□□□□□□□□□□□□□□□□□□□□□□□□□□□□□□□□□□□□□□□□□□□□□□□□□□□□□□□□□□□□□□□□□□□□□□□□□□□□□□□□□□□□□□□□□□□□□□□□□□□□□□□□□□□□□□□□□□□□□□□T

TGG□□□□□□□□□□□□□□□□□□□□□□□□□□□□□□□□□□□□□□□□□□□□□□□□□□□□□□□□□□□□□□□□□□□□□□□□□□□□□□□□□□□□□□□□□□□□□□□□□□□□□□□□□□□□□□□□□□□□□□□□□□□□□□□□□□□□□□□□□□□□□□□□□□□□□□□□□□□□□□□□□□□□□□□□□□□□□□□□□□□□□□□□□□□□□□□□□□□□□□□□□□□□□□□□□□□□□□□□□□□□□□□□□□□□□□□□□□□□□□□□□□□□□□□□□□□□□□□□□□□□□□□□□□□□□□□□□□□□□□□□□□□□□□□□□□□□□□□□□□□□□□□□□□□□□□□□□□□□□□□□□□□□□□□□□□□□□□□□□□□□□□□□□□□□□□□□□□□□□□□□□□□□□□□□□□□□□□□□□□□□□□□□□□□□□□□□□□□□□□□□□□□□□□□□□□□□□□□□□□□□TTA

17p telomere Allele 1

Parental

AAGGG□□□□□□□□□□□□□□□□□□□□□□□□□□□□□□□□□□□□□□□□□□□□□□□□□□□□□□□□□□□□□□□□□□□□□□□□□□□□□□□□□□□□□□□□□□□□□□□□□□□□□□□□□□□□□□□□□□□□□□□□□□□□□□□□□□□□□□□□□□□□□□□□□□□□□□□□□□□□□□□□□□□□□□□□□□□□□TT

AAGGG□□□□□□□□□□□□□□□□□□□□□□□□□□□□□□□□□□□□□□□□□□□□□□□□□□□□□□□□□□□□□□□□□□□□□□□□□□□□□□□□□□□□□□□□□□□□□□□□□□□□□□□□□□□□□□□□□□□□□□□□□□□□□□□□□□□□□□□□□□□□□□□□□□□□□□□□□□□□□□□□□□□□□□□□□□□□□□□□□□□□□□□TT

AAGGG□□□□□□□□□□□□□□□□□□□□□□□□□□□□□□□□□□□□□□□□□□□□□□□□□□□□□□□□□□□□□□□□□□□□□□□□□□□□□□□□□□□□□□□□□□□□□□□□□□□□□□□□□□□□□□□□□□□□□□□□□□□□□□□□□□□□□□□□□□□□□□□□□□□□□□□□□□□□□□□□□□□□□□□□□□□□□□□□□□□□□□□□□□□□□□□□□□□□□□□□□□□□T□□□□□□□□□□□□□□□□□□□□□□□□G□□□□□□TTAG

AAGGG□□□□□□□□□□□□□□□□□□□□□□□□□□□□□□□□□□□□□□□□□□□□□□□□□□□□□□□□□□□□□□□□□□□□□□□□□□□□□□□□□□□□□□□□□□□□□□□□□□□□□□□□□□□□□□□□□□□□□□□□□□□□□□□□□□□□□□□□□□□□□□□□□□□□□□□□□□□□□□□□□□□□□□□□□□□□□□□□□□□□□□□□□□□□□□□□□□□□□□□□□□□□□□□□□□□□□□□□□□□□□□□□□□□□□□□□□□□□□□□□□□□□□□□□□□□□□□□□□□□□TT

AAGGG□□□□□□□□□□□□□□□□□□□□□□□□□□□□□□□□□□□□□□□□□□□□□□□□□□□□□□□□□□□□□□□□□□□□□□□□□□□□□□□□□□□□□□□□□□□□□□□□□□□□□□□□□□□□□□□□□□□□□□□□□□□□□□□□□□□□□□□□□□□□□□□□□□□□□□□□□□□□□□□□□□□□□□□□□□□□□□□□□□□□□□□□□□□□□□□□□□□□□□□□□□□□□□□□□□□□□□□□□□□□□□□□□□□□□□□□□□□□□□□□□□□□□□□□□□□□□□□□□□□□□□□□□□□□□□□□□□□□□□□□□□□□□□□□□□□□□□□□□□□□□□□□□□□□□□□□□□□□□□□□□□□□□□□□□□□□□□□□□□□□□□□□□□□□□□□□□□□□□□□□□□□□□□□□□□□□□□□□□□□□□□□□□□□□□□□□□□□□□□□□□□□□□□□□□□□□□□□□□□□□□□□□□□□□□□□□□□□□□□□□□□□□□□□□□□□□□□□□□□□□□□□□□□□□□□□□□□□□□□□□□□□□□□□□□□□□□TT

AAGG□□□□□□□□□□□□□□□□□□□□□□□□□□□□□□□□□□□□□□□□□□□□□□□□□□□□□□□□□□□□□□□□□□□□□□□□□□□□□□□□□□□□□□□□□□□□□□□□□□□□□□□□□□□□□□□□□□□□□□□□□□□□□□□□□□□□□□□□□□□□□□□□□□□□□□□□□□□□□□□□□□□□□□□□□□□□□□□□□□□□□□□□□□□□□□□□□□□□□□□□□□□□□□□□□□□□□□□□□□□□□□□□□□□□□□□□□□□□□□□□□□□□□□□□□□□□□□□□□□□□□□□□□□□□□TT

AAGGG□□□□□□□□□□□□□□□□□□□□□□□□□□□□□□□□□□□□□□□□□□□□□□□□□□□□□□□□□□□□□□□□□□□□□□□□□□□□□□□□□□□□□□□□□□□□□□□□□□□□□□□□□□□□□□□□□□□□□□□□□□□□□□□□□□□□□□□□□□□□□□□□□□□□□□□□□□□□□□□□□□□□□□□□□□□□□□□□□□□□□□□□□□□TT

ALT-like clone

AAGGG□□□□□□□□□□□□□□□□□□T

AAGGG□□□□□□□□□□□□□□□□□□□□□□□□□□□□□□□□□□□□□□□□□□□□□□□□□□□□TT

AAGGG□□□□□□□□□□□□□□□□□□□□□□□□□□□□□□□□□□□□□□□□□□□□□□□□□□□□□□□□□□□□□□□□□□□□□□□□□□□□□□□□□□□□□□□□□□□□□□□□□□□□□□□□□□□□□□□□□□□□□□□□□□□□□□□□□□□□□□□□□□□□□□□□□□□□□□□□□□□□□□□□□□□□□□□□□□□□□□□□□□□□□□□□□□□□□□□□□□□□□□□□□□□□□□□□□□□□□□□□□□□□□□□□□□□□□□□□□□□□□□□□□□□□□□□□□□□□□□□□□□□□□□□□□□□□□□□□□□□□□□□□□□□□□□□□□□□□□□□□□□□□□□□□□□□□□□□□□□□□□□□□□□□□□□□□□□□□□□□□□□□□□□□□□□□□□□□□□□□□□□□□□□□□□□□□□□□□□□□□□□□□□□□□□□□□□□□□□□□□□□□□□□□□□□□□□□□□□□□□□TT

AAGGG□□□□□□□□□□□□□□□□□□□□□□□□□□□□□□□□□□□□□□□□□□□□□□□□□□□□□□□□□□□□□□□□□□□□□□□□□□□□□□□□□□□□□□□□□□□□□□□□□□□□□□□□□□□□□□□□□□□□□□□□□T

AAGGG□□□□□□□□□□□□□□□□□□□□□□□□□□□□□□□□□□□□□□□□□□□□□□□□□□□□□□□□□□□□□□□□□□□ □□□□□□□□□□□□□□□□□□□□□□□□□□□□□□□□□□□□□□□□□□□□□□□□□□□□□□TT

AAGGG□□□□□□□□□□□□□□□□□□□□□□□□□□□□□□□□□□□□□□□□□□□□□□□□□□□□□□□□□□□□□□□□□□□□□□□□□□□□□□□□□□□□□□□□□□□□□□□□□□□□□□□□□□□□□□□□□□□□□□□□□□□□□□□TT

AAGGG□□□□□□□□□□□□□□□□□□□□□□□□□□□□□□□□□□□□□□□□□□□□□□□□□□□□□□□□□□□□□□□□□□□□□□□□□□□□□□□□□□□□□□□□□□□□□□□□□□□□□□□□□□□□□□□□□□□□□□□□□□□□□□□□□TT

AAGGG□□□□□□□□□□□□□□□□□□□□□□□□□□□□□□□□□□□□□□□□□□□□□□□□□□□□□□□□□□□□□□□□□□□□□□□□□□□□□□□□□□□□□□□□□□□□□□□□□□□□□□□□□□□□□□□□□□□□□□□□□□□□□□□□TT

AAGGG□□□□□□□□□□□□□□□□□□□□□□□□□□□□□□□□□□□□□□□□□□□□□□□□□□□□□□□□□□□□□□□□□□□□□□□□□□□□□□□□□□□□□□□□□□□□□□□□□□□□□□□□□□□□□□□□□□□□□□□□□□□□□□□□□□TT

AAGGG□□□□□□□□□□□□□□□□□□□□□□□□□□□□□□□□□□□□□□□□□□□□□□□□□□□□□□□□□□□□□□□□□□□□□□□□□□□□□□□□□□□□□□□□□□□□□□□□□□□□□□□□□□□□□□□□□□□□□□□□□□□□□□□□□□□□T

AAGGG□□□□□□□□□□□□□□□□□□□□□□□□□□□□□□□□□□□□□□□□□□□□□□□□□□□□□□□□□□□□□□□□□□□□□□□□□□□□□□□□□□□□□□□□□□□□□□□□□□□□□□□□□□□□□□□□□□□□□□□□□□□□□□□□□□□□□TT

AAGGG□□□□□□□□□□□□□□□□□□□□□□□□□□□□□□□□□□□□□□□□□□□□□□□□□□□□□□□□□□□□□□□□□□□□□□□□□□□□□□□□□□□□□□□□□□□□□□□□□□□□□□□□□□□□□□□□□□□□□□□□□□□□□□□□□□□□□□□□□□□□□□□□TT

AAGGG□□□□□□□□□□□□□□□□□□□□□□□□□□□□□□□□□□□□□□□□□□□□□□□□□□□□□□□□□□□□□□□□□□□□□□□□□□□□□□□□□□□□□□□□□□□□□□□□□□□□□□□□□□□□□□□□□□□□□□□□□□□□□□□□□□□□□□□□□□□□□□□TT

AAGGG□□□□□□□□□□□□□□□□□□□□□□□□□□□□□□□□□□□□□□□□□□□□□□□□□□□□□□□□□□□□□□□□□□□□□□□□□□□□□□□□□□□□□□□□□□□□□□□□□□□□□□□□□□□□□□□□□□□□□□□□□□□□□□□□□□□□□□□□□□□□□□□□□□□□□□□□□□□□□TT

AAGGG□□□□□□□□□□□□□□□□□□□□□□□□□□□□□□□□□□□□□□□□□□□□□□□□□□□□□□□□□□□□□□□□□□□□□□□□□□□□□□□□□□□□□□□□□□□□□□□□□□□□□□□□□□□□□□□□□□□□□□□□□□□□□□□□□□□□□□□□□□□□□□□□□□□□□□□□□□□□□□□□□□□□□TT

AAGGG□□□□□□□□□□□□□□□□□□□□□□□□□□□□□□□□□□□□□□□□□□□□□□□□□□□□□□□□□□□□□□□□□□□□□□□□□□□□□□□□□□□□□□□□□□□□□□□□□□□□□□□□□□□□□□□□□□□□□□□□□□□□□□□□□□□□□□□□□□□□□□□□□□□□□□□□□□□□□□□□□□□□□□□□□□□□□□□□□□□□□□TT

AAGGG□□□□□□□□□□□□□□□□□□□□□□□□□□□□□□□□□□□□□□□□□□□□□□□□□□□□□□□□□□□□□□□□□□□□□□□□□□□□□□□□□□□□□□□□□□□□□□□□□□□□□□□□□□□□□□□□□□□□□□□□□□□□□□□□□□□□□□□□□□□□□□□□□□□□□□□□□□□□□□□□□□□□□□□□□□□□□□□□□□□□□□□□□□□□□□□□□TT

AAGGG□□□□□□□□□□□□□□□□□□□□□□□□□□□□□□□□□□□□□□□□□□□□□□□□□□□□□□□□□□□□□□□□□□□□□□□□□□□□□□□□□□□□□□□□□□□□□□□□□□□□□□□□□□□□□□□□□□□□□□□□□□□□□□□□□□□□□□□□□□□□□□□□□□□□□□□□□□□□□□□□□□□□□□□□□□□□□□□□□□□□□□□□□□□□□□□□□□□□□□□□□□□□□□□□□□□□□□□□□□□□□□□□□□□TT

AAGGG□□□□□□□□□□□□□□□□□□□□□□□□□□□□□□□□□□□□□□□□□□□□□□□□□□□□□□□□□□□□□□□□□□□□□□□□□□□□□□□□□□□□□□□□□□□□□□□□□□□□□□□□□□□□□□□□□□□□□□□□□□□□□□□□□□□□□□□□□□□□□□□□□□□□□□□□□□□□□□□□□□□□□□□□□□□□□□□□□□□□□□□□□□□□□□□□□□□□□□□□□□□□□□□□□□□□□□□□□□□□□□□□□□□□□□□□□□□□□□□□□□□□□□□□□□□□□□□□□□□□□TT

AAGGG□□□□□□□□□□□□□□□□□□□□□□□□□□□□□□□□□□□□□□□□□□□□□□□□□□□□□□□□□□□□□□□□□□□□□□□□□□□□□□□□□□□□□□□□□□□□□□□□□□□□□□□□□□□□□□□□□□□□□□□□□□□□□□□□□□□□□□□□□□□□□□□□□□□□□□□□□□□□□□□□□□□□□□□□□□□□□□□□□□□□□□□□□□□□□□□□□□□□□□□□□□□□□□□□□□□□□□□□□□□□□□□□□□□□□□□□□□□□□□□□□□□□□□□□□□□□□□□□□□□□□□□□□□□□□□□□□□□TT

AAGGG□□□□□□□□□□□□□□□□□□□□□□□□□□□□□□□□□□□□□□□□□□□□□□□□□□□□□□□□□□□□□□□□□□□□□□□□□□□□□□□□□□□□□□□□□□□□□□□□□□□□□□□□□□□□□□□□□□□□□□□□□□□□□□□□□□□□□□□□□□□□□□□□□□□□□□□□□□□□□□□□□□□□□□□□□□□□□□□□□□□□□□□□□□□□□□□□□□□□□□□□□□□□□□□□□□□□□□□□□□□□□□□□□□□□□□□□□□□□□□□□□□□□□□□□□□□□□□□□□□□□□□□□□□□□□□□□□□□□□□□□□□□□□□□□□□□□□□TT

AAGGG□□□□□□□□□□□□□□□□□□□□□□□□□□□□□□□□□□□□□□□□□□□□□□□□□□□□□□□□□□□□□□□□□□□□□□□□□□□□□□□□□□□□□□□□□□□□□□□□□□□□□□□□□□□□□□□□□□□□□□□□□□□□□□□□□□□□□□□□□□□□□□□□□□□□□□□□□□□□□□□□□□□□□□□□□□□□□□□□□□□□□□□□□□□□□□□□□□□□□□□□□□□□□□□□□□□□□□□□□□□□□□□□□□□□□□□□□□□□□□□□□□□□ □□□□□□□□□□□□□ □□□□□□□□□□□□□□□□□□□□□□□□□□□□□□□□□□□□□□□□□□□□□TTA

AAGGG□□□□□□□□□□□□□□□□□□□□□□□□□□□□□□□□□□□□□□□□□□□□□□□□□□□□□□□□□□□□□□□□□□□□□□□□□□□□□□□□□□□□□□□□□□□□□□□□□□□□□□□□□□□□□□□□□□□□□□□□□□□□□□□□□□□□□□□□□□□□□□□□□□□□□□□□□□□□□□□□□□□□□□□□□□□□□□□□□□□□□□□□□□□□□□□□□□□□□□□□□□□□□□□□□□□□□□□□□□□□□□□□□□□□□□□□□□□□□□□□□□□□□□□□□□□□□□□□□□□□□□□□□□□□□□□□□□□□□□□□□□□□□□□□□□□□□□□□□□□□□□□□□□□□□□□□□□□□□□□□□□□□□□T

17p telomere Allele 2

Parental

AAGGG□□□□□□□□□□□□□□□□□□□□□□□□□□□□□□□□□□□□□□□TT

AAGGG□□□□□□□□□□□□□□□□□□□□□□□□□□□□□□□□□□□□□□□□□□□□□□□TT

AAGGG□□□□□□□□□□□□□□□□□□□□□□□□□□□□□□□□□□□□□□□□□□□□□□□□□□□□□□□□□□□□□□□□□□□□□□□□□□□□□□□□□□□□□□□□□□□□□□□□□□□□□□□□□□□□□□□□□□□□□□□□□□□□□□□□□TT

AAGGG□□□□□□□□□□□□□□□□□□□□□□□□□□□□□□□□□□□□□□□□□□□□□□□□□□□□□□□□□□□□□□□□□□□□□□□□□□□□□□□□□□□□□□□□□□□□□□□□□□□□□□□□□□□□□□□□□□□□□□□□□□□□□□□□□□□□□□□□□□□□□□□□□□□□□□□□□□□□□□□□□□□□□□□□□□□□□□□□□□□□□TT

AAGGG□□□□□□□□□□□□□□□□□□□□□□□□□□□□□□□□□□□□□□□□□□□□□□□□□□□□□□□□□□□□□□□□□□□□□□□□□□□□□□□□□□□□□□□□□□□□□□□□□□□□□□□□□□□□□□□□□□□□□□□□□□□□□□□□□□□□□□□□□□□□□□□□□□□□□□□□□□□□□□□□□□□□□□□□□□□□□□□□□□□□□□□□□□□□□□□□□□□□□□□□□□□□□□□□□□□□□□□□□□□□□□□□□□□□□□□□□□□□□□□□□□□□□□□□□□□□□□□□□□□□□□TT

AAGGG□□□□□□□□□□□□□□□□□□□□□□□□□□□□□□□□□□□□□□□□□□□□□□□□□□□□□□□□□□□□□□□□□□□□□□□□□□□□□□□□□□□□□□□□□□□□□□□□□□□□□□□□□□□□□□□□□□□□□□□□□□□□□□□□□□□□□□□□□□□□□□□□□□□□□□□□□□□□□□□□□□□□□□□□□□□□□□□□□□□□□□□□□□□□□□□□□□□□□□□□□□□□□□□□□□□□□□□□□□□□□□□□□□□□□□□□□□□□□□□□□□□□□□□□□□□□□□□□□□□□□□□□□□□□□□□□□□□□□□□□□□□□□□□□□□□□□□□□□□□□□□□□□□□□□□□TT

AAGGG□□□□□□□□□□□□□□□□□□□□□□□□□□□□□□□□□□□□□□□□□□□□□□□□□□□□□□□□□□□□□□□□□□□□□□□□□□□□□□□□□□□□□□□□□□□□□□□□□□□□□□□□□□□□□□□□□□□□□□□□□□□□□□□□□□□□□□□□□□□□□□□□□□□□□□□□□□□□□□□□□□□□□□□□□□□□□□□□□□□□□□□□□□□□□□□□□□□□□□□□□□□□□□□□□□□□□□□□□□□□□□□□□□□□□□□□□□□□□□□□□□□□□□□□□□□□□□□□□□□□□□□□□□□□□□□□□□□□□□□□□□□□□□□□□□□□□□□□□□□□□□□□□□□□□□□□□□□□□□□□□□□□□□□□□□TT

AAGGG□□□□□□□□□□□□□□□□□□□□□□□□□□□□□□□□□□□□□□□□□□□□□□□□□□□□□□□□□□□□□□□□□□□□□□□□□□□□□□□□□□□□□□□□□□□□□□□□□□□□□□□□□□□□□□□□□□□□□□□□□□□□□□□□□□□□□□□□□□□□□□□□□□□□□□□□□□□□□□□□□□□□□□□□□□□□□□□□□□□□□□□□□□□□□□□□□□□□□□□□□□□□□□□□□□□□□□□□□□□□□□□□□□□□□□□□□□□□□□□□□□□□□□□□□□□□□□□□□□□□□□□□□□□□□□□□□□□□□□□□□□□□□□□□□□□□□□□□□□□□□□□□□□□□□□□□□□□□□□□□□□□□□□□□□□□□□□□□□□□□□□□□□□□□□□□□□□□□□□□□□□□□□□□□□□□□□□□□□□□□□□□□□□TT

AAGGG□□□□□□□□□□□□□□□□□□□□□□□□□□□□□□□□□T□□□□□□□□□□□□□□□□□□□□□□□□□□□□□□□□□□□□□□□□□□□□□□□□□□□□□□□□□□□□□□□□□□□□□□□□□□□□□□□□□□□□□□□□□□□□□□□□□□□□□□□□□□□□□□□□□□□□□□□□□□□□□□□□□□□□□□□□□□□□□□□□□□□□□□□□□□□□□□□□□□□□□□□□□□□□□□□□□□□□□□□□□□□□□□□□□□□□□□□□□□□□□□□□□□□□□□□□□□□□□□□□□□□□□□□□□□□□□□□□□□□□□□□□□□□□□□□□□□□□□□□□□□□□□□□□□□□□□□□□□□□□□□□□□□□□□□□□□□□□□□□□□□□□□□□□□□□□□□□□□□□□□□□□□□□□□□□□□□□□□□□□□□□□□□□□□□□□□□□□□□□□□□□□□□□□□□□□□□□□□□□□□□□□□□□□□□□□TT

ALT-like clone

AAGGG□□□□□□□□□□□□□□□□T

AAGGG□□□□□□□□□□□□□□□□□□□□□□□□□□□□□□□□□□□□□□□□□□□□□□□□□□□□□□□□□□□□□□□□□□□T

AAGGG□□□□□□□□□□□□□□□□□□□□□□□□□□□□□□□□□□□□□□□□□□□□□□□□□□□□□□□□□□□□□□□□□□□□□□□□□□□□□□□□□□□□□□□□□□□□□□□□□□□TT

AAGGG□□□□□□□□□□□□□□□□□□□□□□□□□□□□□□□□□□□□□□□□□□□□□□□□□□□□□□□□□□□□□□□□□□□□□□□□□□□□□□□□□TT

AAGGG□□□□□□□□□□□□□□□□□□□□□□□□□□□□□□□□□□□□□□□□□□□□□□□□□□□□□□□□□□□□□□□□□□□□□□□□□□□□□□□□□□□□□□□TT

AAGGG□□□□□□□□□□□□□□□□□□□□□□□□□□□□□□□□□□□□□□□□□□□□□□□□□□□□□□□□□□□□□□□□□□□□□□□□□□□□□□□□□□□□□□□□□TT

AAGGG□□□□□□□□□□□□□□□□□□□□□□□□□□□□□□□□□□□□□□□□□□□□□□□□□□□□□□□□□□□□□□□□□□□□□□□□□□□□□□□□□□□□□□□□□□□□□□□□□□□□TTA

AAGGG□□□□□□□□□□□□□□□□□□□□□□□□□□□□□□□□□□□□□□□□□□□□□□□□□□□□□□□□□□□□□□□□□□□□□□□□□□□□□□□□□□□□□□□□□□□□□□□□□□□□□□□□□T□□□□□□□□□□□□□□□□□□□□□□□□□T

AAGGG□□□□□□□□□□□□□□□□□□□□□□□□□□□□□□□□□□□□□□□□□□□□□□□□□□□□□□□□□□□□□□□□□□□□□□□□□□□□□□□□□□□□□□□□□□□□□□□□□□□□□□□□□□□□□□□□□□□□□□□□□□□□□□□□□□□□TT

AAGGG□□□□□□□□□□□□□□□□□□□□□□□□□□□□□□□□□□□□□□□□□□□□□□□□□□□□□□□□□□□□□□□□□□□□□□□□□□□□□□□□□□□□□□□□□□□□□□□□□□□□□□□□□□□□□□□□□□□□□□□□□□□□□□□□□□□□□□□□□□□□□□□□□□□□□□□□□□□□□□□□□□□□TTA

AAGGG□□□□□□□□□□□□□□□□□□□□□□□□□□□□□□□□□□□□□□□□□□□□□□□□□□□□□□□□□□□□□□□□□□□□□□□□□□□□□□□□□□□□□□□□□□□□□□□□□□□□□□□□□□□□□□□□□□□□□□□□□□□□□□□□□□□□□□□□□□□□□□□□□□□□□□□□□□□□□□□□□□□□□□□□□□□□□□T

AAGGG□□□□□□□□□□□□□□□□□□□□□□□□□□□□□□□□□□□□□□□□□□□□□□□□□□□□□□□□□□□□□□□□□□□□□□□□□□□□□□□□□□□□□□□□□□□□□□□□□□□□□□□□□□□□□□□□□□□□□□□□□□□□□□□□□□□□□□□□□□□□□□□□□□□□□□□□□□□□□□□□□□□□□□□□□□□□□□□□□□□□□□□□□□□□□□□□□□□□□□□T

AAGGG□□□□□□□□□□□□□□□□□□□□□□□□□□□□□□□□□□□□□□□□□□□□□□□□□□□□□□□□□□□□□□□□□□□□□□□□□□□□□□□□□□□□□□□□□□□□□□□□□□□□□□□□□□□□□□□□□□□□□□□□□□□□□□□□□□□□□□□□□□□□□□□□□□□□□□□□□□□□□□□□□□□□□□□□□□□□□□□□□□□□□□□□□□□□□□□□□□□□□□□□□□T□□□□□□□□□□□□□□□□□□□□□□□T

AAGGG□□□□□□□□□□□□□□□□□□□□□□□□□□□□□□□□□□□□□□□□□□□□□□□□□□□□□□□□□□□□□□□□□□□□□□□□□□□□□□□□□□□□□□□□□□□□□□□□□□□□□□□□□□□□□□□□□□□□□□□□□□□□□□□□□□□□□□□□□□□□□□□□□□□□□□□□□□□□□□□□□□□□□□□□□□□□□□□□□□□□□□□□□□□□□□□□□□□□□□□□□□□□□□□□□□□□□□□□□□□□□□□□□□□□□□□□□□□□TT

AAGGG□□□□□□□□□□□□□□□□□□□□□□□□□□□□□□□□□□□□□□□□□□□□□□□□□□□□□□□□□□□□□□□□□□□□□□□□□□□□□□□□□□□□□□□□□□□□□□□□□□□□□□□□□□□□□□□□□□□□□□□□□□□□□□□□□□□□□□□□□□□□□□□□□□□□□□□□□□□□□□□□□□□□□□□□□□□□□□□□□□□□□□□□□□□□□□□□□□□□□□□□□□□□□□□□□□□□□□□□□□□□□□□□□□□□□□□□□□□□□□□□TT

AAGGG□□□□□□□□□□□□□□□□□□□□□□□□□□□□□□□□□□□□□□□□□□□□□□□□□□□□□□□□□□□□□□□□□□□□□□□□□□□□□□□□□□□□□□□□□□□□□□□□□□□□□□□□□□□□□□□□□□□□□□□□□□□□□□□□□□□□□□□□□□□□□□□□□□□□□□□□□□□□□□□□□□□□□□□□□□□□□□□□□□□□□□□□□□□□□□□□□□□□□□□□□□□□□□□□□□□□□□□□□□□□□□□□□□□□□□□□□□□□□□□□□□TT

AAGGG□□□□□□□□□□□□□□□□□□□□□□□□□□□□□□□□□□□□□□□□□□□□□□□□□□□□□□□□□□□□□□□□□□□□□□□□□□□□□□□□□□□□□□□□□□□□□□□□□□□□□□□□□□□□□□□□□□□□□□□□□□□□□□□□□□□□□□□□□□□□□□□□□□□□□□□□□□□□□□□□□□□□□□□□□□□□□□□□□□□□□□□□□□□□□□□□□□□□□□□□□□□□□□□□□□□□□□□□□□□□□□□□□□□□□□□□□□□□□□□□□□□□□□T

AAGGG□□□□□□□□□□□□□□□□□□□□□□□□□□□□□□□□□□□□□□□□□□□□□□□□□□□□□□□□□□□□□□□□□□□□□□□□□□□□□□□□□□□□□□□□□□□□□□□□□□□□□□□□□□□□□□□□□□□□□□□□□□□□□□□□□□□□□□□□□□□□□□□□□□□□□□□□□□□□□□□□□□□□□□□□□□□□□□□□□□□□□□□□□□□□□□□□□□□□□□□□□□□□□□□□□□□□□□□□□□□□□□□□□□□□□□□□□□□□□□□□□□□□□□□□□□□□□□□□□□□□□□□TT

AAGGG□□□□□□□□□□□□□□□□□□□□□□□□□□□□□□□□□□□□□□□□□□□□□□□□□□□□□□□□□□□□□□□□□□□□□□□□□□□□□□□□□□□□□□□□□□□□□□□□□□□□□□□□□□□□□□□□□□□□□□□□□□□□□□□□□□□□□□□□□□□□□□□□□□□□□□□□□□□□□□□□□□□□□□□□□□□□□□□□□□□□□□□□□□□□□□□□□□□□□□□□□□□□□□□□□□□□□□□□□□□□□□□□□□□□□□□□□□□□□□□□□□□□□□□□□□□□□□□□□□□□□□□□□□□□□□□□□□□□□□□□□□□□□TT

AAGGG□□□□□□□□□□□□□□□□□□□□□□□□□□□□□□□□□□□□□□□□□□□□□□□□□□□□□□□□□□□□□□□□□□□□□□□□□□□□□□□□□□□□□□□□□□□□□□□□□□□□□□□□□□□□□□□□□□□□□□□□□□□□□□□□□□□□□□□□□□□□□□□□□□□□□□□□□□□□□□□□□□□□□□□□□□□□□□□□□□□□□□□□□□□□□□□□□□□□□□□□□□□□□□□□□□□□□□□□□□□□□□□□□□□□□□□□□□□□□□□□□□□□□□□□□□□□□□□□□□□□□□□□□□□□□□T□□□□□□□□□□□□□□□□□□□□□□□□□□□□□□□□□□□□□□□□□□□□□□□□TTA

AAGGG□□□□□□□□□□□□□□□□□□□□□□□□□□□□□□□□□□□□□□□□□□□□□□□□□□□□□□□□□□□□□□□□□□□□□□□□□□□□□□□□□□□□□□□□□□□□□□□□□□□□□□□□□□□□□□□□□□□□□□□□□□□□□□□□□□□□□□□□□□□□□□□□□□□□□□□□□□□□□□□□□□□□□□□□□□□□□□□□□□□□□□□□□□□□□□□□□□□□□□□□□□□□□□□□□□□□□□□□□□□□□□□□□□□□□□□□□□□□□□□□□□□□□□□□□□□□□□□□□□□□□□□□□□□□□□□□□□□□□□□□□□□□□□□□□□□□□□□□□□□□□□□□□□□□□□□□□□□□□□□□□□□□TG

AAGGG□□□□□□□□□□□□□□□□□□□□□□□□□□□□□□□□□□□□□□□□□□□□□□□□□□□□□□□□□□□□□□□□□□□□□□□□□□□□□□□□□□□□□□□□□□□□□□□□□□□□□□□□□□□□□□□□□□□□□□□□□□□□□□□□□□□□□□□□□□□□□□□□□□□□□□□□□□□□□□□□□□□□□□□□□□□□□□□□□□□□□□□□□□□□□□□□□□□□□□□□□□□□□□□□□□□□□□□□□□□□□□□□□□□□□□□□□□□□□□□□□□□□□□□□□□□□□□□□□□□□□□□□□□□□□□□□□□□□□□□□□□□□□□□□□□□□□□□□□□□□□□□□□□□□□□□□□□□□□□□□□□□□□□□□□□□□□□□□TT

AAGGG□□□□□□□□□□□□□□□□□□□□□□□□□□□□□□□□□□□□□□□□□□□□□□□□□□□□□□□□□□□□□□□□□□□□□□□□□□□□□□□□□□□□□□□□□□□□□□□□□□□□□□□□□□□□□□□□□□□□□□□□□□□□□□□□□□□□□□□□□□□□□□□□□□□□□□□□□□□□□□□□□□□□□□□□□□□□□□□□□□□□□□□□□□□□□□□□□□□□□□□□□□□□□□□□□□□□T□□□□□□□□□□□□□□□□□□□□□□□□□□□□□□□□□□□□□□□□□□□□□□□□□□□□□□□□□□□□□□□□□□□□□□□□□□□□□□□□□□□□□□□□□□□□□□□□□□□□□□□□□□□□□□□□□□□□□□□□□□□□□□□□□□□□□□□□□□□□□□□□□□□□□□□□□□□□□□T

AAGGG□□□□□□□□□□□□□□□□□□□□□□□□□□□□□□□□□□□□□□□□□□□□□□□□□□□□□□□□□□□□□□□□□□□□□□□□□□□□□□□□□□□□□□□□□□□□□□□□□□□□□□□□□□□□□□□□□□□□□□□□□□□□□□□□□□□□□□□□□□□□□□□□□□□□□□□□□□□□□□□□□□□□□□□□□□□□□□□□□□□□□□□□□□□□□□□□□□□□□□□□□□□□□□□□□□□□□□□□□□□□□□□□□□□□□□□□□□□□□□□□□□□□□□□□□□□□□□□□□□□□□□□□□□□□□□□□□□□□□□□□□□□□□□□□□□□□□□□□□□□□□□□□□□□□□□□□□□□□□□□□□□□□□□□□□□□□□□□□□□□□□□□□□□□□□□□□□□□□□□□□□□□□□□□□□□□□□□□□□□□□□□□□□□□□□□□□□□□□□□□□□□□□□□□□□□T

AAGGG□□□□□□□□□□□□□□□□□□□□□□□□□□□□□□□G□□□□□□□□□□□□□□□□□□□□□□□□□□□□□□□□□□□□□□□□□□□□□□□□□□□□□□□□□□□□□□□□□□□□□□□□□□□□□□□□□□□□□□□□□□□□□□□□□□□□□□□□□□□□□□□□□□□□□□□□□□□□□□□□□□□□□□□□□□□□□□□□□□□□□□□□□□□□□□□□□□□□□□□□□□□□□□□□□□□□□□□□□□□□□□□□□□□□□□□□□□□□□□□□□□□□□□□□□□□□□□□□□□□□□□□□□□□□□□□□□□□□□□□□□□□□□□□□□□□□□□□□□□□□□□□□□□□□□□□□□□□□□□□□□□□□□□□□□□□□□□□□□□□□□□□□□□□□□□□□□□□□□□□□□□□□□□□□□□□□□□□□□□□□□□□□□□□□□□□□□□□□□□□□□□□□□□□□□□□□□□□TT

AAGGG□□□□□□□□□□□□□□□□□□□□□□□□□□□□□□□□□□□□□□□□□□□□□□□□□□□□□□□□□□□□□□□□□□□□□□□□□□□□□□□□□□□□□□T□□□□□□□□□□□□□□□□□□□□□□□□□□□□□□□□□□□□□□□□□□□□□□□□□□□□□□□□□□□□□□□□□□□□□□□□□□□□□□□□□□□□□□□□□□□□□□□□□□□□□□□□□□□□□□□□□□□□□□□□□□□□□□□□□□□□□□□□□□□□□□□□□□□□□□□□□□□□□□□□□□□□□□□□□□□□□□□□□□□□□□□□□□□□□□□□□□□□□□□□□□□□□□□□□□□□□□□□□□□□□□□□□□□□□□□□□□□□□□□□□□□□□□□□□□□□□□□□□□□□□□□□□□□□□□□□□□□□□□□□□□□□□□□□□□□□□□□□□□□□□□□□□□□□□□□□□□□□□□□□□□□□□□□□□□□□□□

7q telomere Allele 1

Parental

AGTG□□□□□□□□□□□□□□□□□□□TT

AGTG□□□□□□□□□□□□□□□□□□□□□□□□□□□□□□□□□□□□□□□□□□□□□□□□□□□□□□□□□□□□□□□□□□□□□□□□□□□□□□□□□□□□□□□□□□□□□□□□□□□□□□□□□□□□□□□□□□□□□□□□□□□□□□□□□□□□□□□□□□□□□□□□□□□□□□□□□□□□□□□□□□□□□□□□□□□□□□□□□□□□□□□□□□TT

AGTG□□□□□□□□□□□□□□□□□□□□□□□□□□□□□□□□□□□□□□□□□□□□□□□□□□□□□□□□□□□□□□□□□□□□□□□□□□□□□□□□□□□□□□□□□□□□□□□□□□□□□□□□□□□□□□□□□□□□□□□□□□□□□□□□□□□□□□□□T□□□□□□□□□□□□□□□□□□□□□□□□□□□□□□□□□□□□□□□□□□□□□□□□□□□□□□□□□□□□□□□□□□□□□□□□□□□□□□□□□□□□□□□□□□□□□□□□□□□□□TT

AGTG□□□□□□□□□□□□□□□□□□□□□□□□□□□□□□□□□□□□□□□□□□□□□□□□□□□□□□□□□□□□□□□□□□□□□□□□□□□□□□□□□□□□□□□□□□□□□□□□□□□□□□□□□□□□□□□□□□□□□□□□□□□□□□□□□□□□□□□□□□□□□□□□□□□□□□□□□□□□□□□□□□□□□□□□□□□□□□□□□□□□□□□□□□□□□□□□□□□□□□□□□□□□□□□□□□□□□□□□□□□□□□□□□□□□□□□□□□□□□□□□□□□□□□□□□□□□□□□□□□□□□□□□□□□□□□□□□□□□T

AGTG□□□□□□□□□□□□□□□□□□□□□□□□□□□□□□□□□□□□□□□□□□□□□□□□□□□□□□□□□□□□□□□□□□□□□□□□□□□□□□□□□□□□□□□□□□□□□□□□□□□□□□□□□□□□□□□□□□□□□□□□□□□□□□□□□□□□□□□□□□□□□□□□□□□□□□□□□□□□□□□□□□□□□□□□□□□□□□□□□□□□□□□□□□□□□□□□□□□□□□□□□□□□□□□□□□□□□□□□□□□□□□□□□□□□□□□□□□□□□□□□□□□□□□□□□□□□□□□□□□□□□□□□□□□□□□□□□□□□□□□□□□□□□□□□□□□□□□□□□□□□□□□□□□□□□□□□□□□□□□□□□□□□□□TT

AGTG□□□□□□□□□□□□□□□□□□□□□□□□□□□□□□□□□□□□□□□□□□□□□□□□□□□□□□□□□□□□□□□□□□□□□□□□□□□□□□□□□□□□□□□□□□□□□□□□□□□□□□□□□□□□□□□□□□□□□□□□□□□□□□□□□□□□□□□□□□□□□□□□□□□□□□□□□□□□□□□□□□□□□□□□□□□□□□□□□□□□□□□□□□□□□□□□□□□□□□□□□□□□□□□□□□□□□□□□□□□□□□□□□□□□□□□□□□□□□□□□□□□□□□□□□□□□□□□□□□□□□□□□□□□□□□□□□□□□□□□□□□□□□□□□□□□□□□□□□□□□□□□□□□□□□□□□□□□□□□□□□□□□□□□□□□□□□□□□□□□□□□□□□□□□□□□□□□□□□□□□□□□□□□□□□□□□□□□□□□□□□□□□□□□□□□□□□□□□□□□TT

AGTG□□□□□□□□□□□□□□□□□□□□□□□□□□□□□□□□□□□□□□□□□□□□□□□□□□□□□□□□□□□□□□□□□□□□□□□□□□□□□□□□□□□□□□□□□□□□□□□□□□□□□□□□□□□□□□□□□□□□□□□□□□□□□□□□□□□□□□□□□□□□□□□□□□□□□□□□□□□□□□□□□□□□□□□□□□□□□□□□□□□□□□□□□□□□□□□□□□□□□□□□□□□□□□□□□□□□□□□□□□□□□□□□□□□□□□□□□□□□□□□□□□□□□□□□□□□□□□□□□□□□□□□□□□□□□□□□□□□□□□□□□□□□□□□□□□□□□□□□□□□□□□□□□□□□□□□□□□□□□□□□□□□□□□□□□□□□□□□□□□□□□□□□□□□□□□□□□□□□□□□□□□□□□□□□□□□□□□□□□□□□□□□□□□□□□□□□□□□□□□□□□□□□□□□□□□□□□□□□□□□□□□□□□□□□□□□□□□□□□□□□□□□□□□TT

AGTG□□□□□□□□□□□□□□□□□□□□□□□□□□□□□□□□□□□□□□□□□□□□□□□□□□□□□□□□□□□□□□□□□□□□□□□□□□□□□□□□□□□□□□□□□□□□□□□□□□□□□□□□□□□□□□□□□□□□□□□□□□□□□□□□□□□□□□□□□□□□□□□□□□□□□□□□□□□□□□□□□□□□□□□□□□□□□□□□□□□□□□□□□□□□□□□□□□□□□□□□□□□□□□□□□□□□□□□□□□□□□□□□□□□□□□□□□□□□□□□□□□□□□□□□□□□□□□□□□□□□□□□□□□□□□□□□□□□□□□□□□□□□□□□□□□□□□□□□□□□□□□□□□□□□□□□□□□□□□□□□□□□□G□□□□□□□□□□□□□□□□□□□□□□□□□□□□□□□□□□□□□□□□□□□□□□□T□□□□□□□□□□□□□□□□□□□□□□□□□□□□□□□□□□□□□□□□□□□□□□□□□□□□□□□□□□□□□□□□□□□□□□□□□□□□□□□□□□□□□□□□□□□□□□□□□□□□□□□□□□□□□□□□□□□□□□□□□□□□□□□□□□□□□□□□□□□□□□□□□□□□□□□□□□□□□□□□□□□□□□□□□□□□□□□□□□□□□□□□□□□□□□□□□□□□□□□□□□□TTA

ALT-like clone

AGTG□□□□□□□□□□□□□□□□□□□□□□□□□□T

AGTG□□□□□□□□□□□□□□□□□□□□□□□□□□□□□□□□□□□□□□□□□□□□□□□□□□□□□□□□□□□□□□□□□□□□□□□□

AGTG□□□□□□□□□□□□□□□□□□□□□□□□□□□□□□□□□□□□□□□□□□□□□□□□□□□□□□□□□□□□□□□□□□□□□□□□□□□□□□□T

AGTG□□□□□□□□□□□□□□□□□□□□□□□□□□□□□□□□□□□□□□□□□□□□□□□□□□□□□□□□□□□□□□□□□□□□□□□□□□□□□□□□□□□□□□□□□□□□□□□□□□□□□□□□□□□□□□□□□□□□□□□□□□□□□□□□□□□□□□□□□□□□□□□□□□□□□□□□□□□□□□□□□□□□□□□□□□□□□□□□□□□□□□□□□□□□□□□□□□□□□□□□□□□□□□□□□□□□□□□□□□□□□□□□□□□□□□□□□□□□□□□□□□□□□□□□□□□□□□□□□□□□□□□□□□□□□□□□□□□□□□□□□□□□□□□□□□□□□□□□□□□□□□□□□□□□□□□□□□□□□□□□□□□□□□□□□□□□□□□□□□□□□□□□□□□□□□□□□□□□□□□□□□□□□□□□□□□□□□□□□□□□□□□□□□□□□□□□□□□□□□□□□□□TTT

AGTG□□□□□□□□□□□□□□□□□□□□□□□□□□□□□□□□□□□□□□□□□□□□□□□□□□□□□□□□□□□□□□□□□□□□□□□□□□□□□□□□□□□□□□□□□□□□□□□□□□□□□□□□□□□□□□□□□□□□□□□TT

AGTG□□□□□□□□□□□□□□□□□□□□□□□□□□□□□□□□□□□□□□□□□□□□□□□□□□□□□□□□□□□□□□□□□□□□□□□□□□□□□□□□□□□□□□□□□□□□□□□□□□□□□□□□□□□□□□□□□□□□□□□□□□□□□□□□□□□□□□□□□□□□□□□□□□□□□□□□□□□□□□□□□□□□□□□□□□□□□□□□□TT

AGTG□□□□□□□□□□□□□□□□□□□□□□□□□□□□□□□□□□□□□□□□□□□□□□□□□□□□□□□□□□□□□□□□□□□□□□□□□□□□□□□□□□□□□□□□□□□□□□□□□□□□□□□□□□□□□□□□□□□□□□□□□□□□□□□□□□□□□□□□□□□□□□□□□□□□□□□□□□□□□□□□□□□□□□□□□□□□□□□□□□□□□□□□□□□□□□□□□□□□□□□□□□□□□□□□□□□□□□□TT

AGTG□□□□□□□□□□□□□□□□□□□□□□□□□□□□□□□□□□□□□□□□□□□□□□□□□□□□□□□□□□□□□□□□□□□□□□□□□□□□□□□□□□□□□□□□□□□□□□□□□□□□□□□□□□□□□□□□□□□□□□□□□□□□□□□□□□□□□□□□□□□□□□□□□□□□□□□□□□□□□□□□□□□□□□□□□□□□□□□□□□□□□□□□□□□□□□□□□□□□□□□□□□□□□□□□□□□□□□□□□□TT

AGTG□□□□□□□□□□□□□□□□□□□□□□□□□□□□□□□□□□□□□□□□□□□□□□□□□□□□□□□□□□□□□□□□□□□□□□□□□□□□□□□□□□□□□□□□□□□□□□□□□□□□□□□□□□□□□□□□□□□□□□□□□□□□□□□□□□□□□□□□□□□□□□□□□□□□□□□□□□□□□□□□□□□□□□□□□□□□□□□□□□□□□□□□□□□□□□□□□□□□□□□□□□□□□□□□□□□□□□□□□□□□□□□□□□T

AGTG□□□□□□□□□□□□□□□□□□□□□□□□□□□□□□□□□□□□□□□□□□□□□□□□□□□□□□□□□□□□□□□□□□□□□□□□□□□□□□□□□□□□□□□□□□□□□□□□□□□□□□□□□□□□□□□□□□□□□□□□□□□□□□□□□□□□□□□□□□□□□□□□□□□□□□□□□□□□□□□□□□□□□□□□□□□□□□□□□□□□□□□□□□□□□□□□□□□□□□□□□□□□□□□□□□□□□□□□□□□□□□□□□□□□□□□□□□TTTA

AGTG□□□□□□□□□□□□□□□□□□□□□□□□□□□□□□□□□□□□□□□□□□□□□□□□□□□□□□□□□□□□□□□□□□□□□□□□□□□□□□□□□□□□□□□□□□□□□□□□□□□□□□□□□□□□□□□□□□□□□□□□□□□□□□□□□□□□□□□□□□□□□□□□□□□□□□□□□□□□□□□□□□□□□□□□□□□□□□□□□□□□□□□□□□□□□□□□□□□□□□□□□□□□□□□□□□□□□□□□□□□□□□□□□□□□□□□□□□□□□□□□□□□□TT

AGTG□□□□□□□□□□□□□□□□□□□□□□□□□□□□□□□□□□□□□□□□□□□□□□□□□□□□□□□□□□□□□□□□□□□□□□□□□□□□□□□□□□□□□□□□□□□□□□□□□□□□□□□□□□□□□□□□□□□□□□□□□□□□□□□□□□□□□□□□□□□□□□□□□□□□□□□□□□□□□□□□□□□□□□□□□□□□□□□□□□□□□□□□□□□□□□□□□□□□□□□□□□□□□□□□□□□□□□□□□□□□□□□□□□□□□□□□□□□□□□□□□□□□□□□□TT

AGTG□□□□□□□□□□□□□□□□□□□□□□□□□□□□□□□□□□□□□□□□□□□□□□□□□□□□□□□□□□□□□□□□□□□□□□□□□□□□□□□□□□□□□□□□□□□□□□□□□□□□□□□□□□□□□□□□□□□□□□□□□□□□□□□□□□□□□□□□□□□□□□□□□□□□□□□□□□□□□□□□□□□□□□□□□□□□□□□□□□□□□□□□□□□□□□□□□□□□□□□□□□□□□□□□□□□□□□□□□□□□□□□□□□□□□□□□□□□□□□□□□□□□□□□□□□□□□□□□□□TTA

GGTG□□□□□□□□□□□□□□□□□□□□□□□□□□□□□□□□□□□□□□□□□□□□□□□□□□□□□□□□□□□□□□□□□□□□□□□□□□□□□□□□□□□□□□□□□□□□□□□□□□□□□□□□□□□□□□□□□□□□□□□□□□□□□□□□□□□□□□□□□□□□□□□□□□□□□□□□□□□□□□□□□□□□□□□□□□□□□□□□□□□□□□□□□□□□□□□□□□□□□□□□□□□□□□□□□□□□□□□□□□□□□□□□□□□□□□□□□□□□□□□□□□□□□□□□□□□□□□□□□□□□□□□□□□TT

AGTG□□□□□□□□□□□□□□□□□□□□□□□□□□□□□□□□□□□□□□□□□□□□□□□□□□□□□□□□□□□□□□□□□□□□□□□□□□□□□□□□□□□□□□□□□□□□□□□□□□□□□□□□□□□□□□□□□□□□□□□□□□□□□□□□□□□□□□□□□□□□□□□□□□□□□□□□□□□□□□□□□□□□□□□□□□□□□□□□□□□□□□□□□□□□□□□□□□□□□□□□□□□□□□□□□□□□□□□□□□□□□□□□□□□□□□□□□□□□□□□□□□□□□□□□□□□□□□□□□□□□□□□□□□□□□□□□□□□□□□□□□□□□□TT

AGTG□□□□□□□□□□□□□□□□□□□□□□□□□□□□□□□□□□□□□□□□□□□□□□□□□□□□□□□□□□□□□□□□□□□□□□□□□□□□□□□□□□□□□□□□□□□□□□□□□□□□□□□□□□□□□□□□□□□□□□□□□□□□□□□□□□□□□□□□□□□□□□□□□□□□□□□□□□□□□□□□□□□□□□□□□□□□□□□□□□□□□□□□□□□□□□□□□□□□□□□□□□□□□□□□□□□□□□□□□□□□□□□□□□□□□□□□□□□□□□□□□□□□□□□□□□□□□□□□□□□□□□□□□□□□□□□□□□□□□□□□□□□□□□□□□□□□□□□□□□□□□□□□□□□□TTCGC

AGTG□□□□□□□□□□□□□□□□□□□□□□□□□□□□□□□□□□□□□□□□□□□□□□□□□□□□□□□□□□□□□□□□□□□□□□□□□□□□□□□□□□□□□□□□□□□□□□□□□□□□□□□□□□□□□□□□□□□□□□□□□□□□□□□□□□□□□□□□□□□□□□□□□□□□□□□□□□□□□□□□□□□□□□□□□□□□□□□□□□□□□□□□□□□□□□□□□□□□□□□□□□□□□□□□□□□□□□□□□□□□□□□□□□□□□□□□□□□□□□□□□□□□□□□□□□□□□□□□□□□□□□□□□□□□□□□□□□□□□□□□□□□□□□□□□□□□□□□□□□□□□□□□□□□□□□□□□□□□□□□□□TT

AGTG□□□□□□□□□□□□□□□□□□□□□□□□□□□□□□□□□□□□□□□□□□□□□□□□□□□□□□□□□□□□□□□□□□□□□□□□□□□□□□□□□□□□□□□□□□□□□□□□□□□□□□□□□□□□□□□□□□□□□□□□□□□□□□□□□□□□□□□□□□□□□□□□□□□□□□□□□□□□□□□□□□□□□□□□□□□□□□□□□□□□□□□□□□□□□□□□□□□□□□□□□□□□□□□□□□□□□□□□□□□□□□□□□□□□□□□□□□□□□□□□□□□□□□□□□□□□□□□□□□□□□□□□□□□□□□□□□□□□□□□□□□□□□□□□□□□□□□□□□□□□□□□□□□□□□□□□□□□□□□□□□□□□□□□□TT

AGTG□□□□□□□□□□□□□□□□□□□□□□□□□□□□□□□□□□□□□□□□□□□□□□□□□□□□□□□□□□□□□□□□□□□□□□□□□□□□□□□□□□□□□□□□□□□□□□□□□□□□□□□□□□□□□□□□□□□□□□□□□□□□□□□□□□□□□□□□□□□□□□□□□□□□□□□□□□□□□□□□□□□□□□□□□□□□□□□□□□□□□□□□□□□□□□□□□□□□□□□□□□□□□□□□□□□□□□□□□□□□□□□□□□□□□□□□□□□□□□□□□□□□□□□□□□□□□□□□□□□□□□□□□□□□□□□□□□□□□□□□□□□□□□□□□□□□□□□□□□□□□□□□□□□□□□□□□□□□□□□□□□□□□□□□□□□□□□□□□□□□□□□□□□□□□□□□□□□□□□□□□□□□□□□□TTAGG

AGTG□□□□□□□□□□□□□□□□□□□□□□□□□□□□□□□□□□□□□□□□□□□□□□□□□□□□□□□□□□□□□□□□□□□□□□□□□□□□□□□□□□□□□□□□□□□□□□□□□□□□□□□□□□□□□□□□□□□□□□□□□□□□□□□□□□□□□□□□□□□□□□□□□□□□□□□□□□□□□□□□□□□□□□□□□□□□□□□□□□□□□□□□□□□□□□□□□□□□□□□□□□□□□□□□□□□□□□□□□□□□□□□□□□□□□□□□□□□□□□□□□□□□□□□□□□□□□□□□□□□□□□□□□□□□□□□□□□□□□□□□□□□□□□□□□□□□□□□□□□□□□□□□□□□□□□□□□□□□□□□□□□□□□□□□□□□□□□□□□□□□□□□□□□□□□□□□□□□□□□□□□□□□□□□□□□□□TT

AGTG□□□□□□□□□□□□□□□□□□□□□□□□□□□□□□□□□□□□□□□□□□□□□□□□□□□□□□□□□□□□□□□□□□□□□□□□□□□□□□□□□□□□□□□□□□□□□□□□□□□□□□□□□□□□□□□□□□□□□□□□□□□□□□□□□□□□□□□□□□□□□□□□□□□□□□□□□□□□□□□□□□□□□□□□□□□□□□□□□□□□□□□□□□□□□□□□□□□□□□□□□□□□□□□□□□□□□□□□□□□□□□□□□□□□□□□□□□□□□□□□□□□□□□□□□□□□□□□□□□□□□□□□□□□□□□□□□□□□□□□□□□□□□□□□□□□□□□□□□□□□□□□□□□□□□□□□□□□□□□□□□□□□□□□□□□□□□□□□□□□□□□□□□□□□□□□□□□□□□□□□□□□□□□□□□□□□□□□□□G□□□□□□□□□□□□□□□□□□□□□□□□□□□□□□□□□□□□□□□□□□□□□□□□□□□□□□□□□□□□□□□□□□□□□□□□□□□□□□□□□□□□□□□□□□□□□□□□□□□□□□□□□□□□□□□□□□□□□□□□□□□□□□□□□□□□□□□□□□□□□□□□□□□□□□□□□□□□□□□TTAT

7q telomere allele 2

Parental

AGTG□□□□□□□□□□□□□□□□□□□□□□□□□□□□□□□□□□□□□□□□□□□□□□□□□□□□□□TT

AGTG□□□□□□□□□□□□□□□□□□□□□□□□□□□□□□□□□□□□□□□□□□□□□□□□□□□□□□□□□□□□□□□□□□□□□□□□□□□□□□□□□□□□□□□□□□□□□□□□□□□□□□□□□□□□□□□□□□□□□□□□□□□□□□□□□□□□□□□□□□□□□□□□□□□□□□□□□□□□□□□□□□□□□□□□□□□□□□□□□□□□□□TT

AGTG□□□□□□□□□□□□□□□□□□□□□□□□□□□□□□□□□□□□□□□□□□□□□□□□□□□□□□□□□□□□□□□□□□□□□□□□□□□□□□□□□□□□□□□□□□□□□□□□□□□□□□□□□□□□□□□□□□□□□□□□□□□□□□□□□□□□□□□□□□□□□□□□□□□□□□□□□□□□□□□□□□□□□□□□□□□□□□□□□□□□□□□□□□□□□□□□□□□□□□□□□□□□□□□□□□□□□□□□□□□□□□□□□□□□□□□□□□T

AGTG□□□□□□□□□□□□□□□□□□□□□□□□□□□□□□□□□□□□□□□□□□□□□□□□□□□□□□□□□□□□□□□□□□□□□□□□□□□□□□□□□□□□□□□□□□□□□□□□□□□□□□□□□□□□□□□□□□□□□□□□□□□□□□□□□□□□□□□□□□□□□□□□□□□□□□□□□□□□□□□□□□□□□□□□□□□□□□□□□□□□□□□□□□□□□□□□□□□□□□□□□□□□□□□□□□□□□□□□□□□□□□□□□□□□□□□□□□□□□□□□□□□□□□□□□□□□□□□□□□□□□□□□□□□□□□TT

AGTG□□□□□□□□□□□□□□□□□□□□□□□□□□□□□□□□□□□□□□□□□□□□□□□□□□□□□□□□□□□□□□□□□□□□□□□□□□□□□□□□□□□□□□□□□□□□□□□□□□□□□□□□□□□□□□□□□□□□□□□□□□□□□□□□□□□□□□□□□□□□□□□□□□□□□□□□□□□□□□□□□□□□□□□□□□□□□□□□□□□□□□□□□□□□□□□□□□□□□□□□□□□□□□□□□□□□□□□□□□□□□□□□□□□□□□□□□□□□□□□□□□□□□□□□□□□□□□□□□□□□□□□□□□□□□□□□□□□□□□□□□□□□□□□□□□□□□□□□□□□□□□□□□□□□□□□□□□□□□□□TT

AGTG□□□□□□□□□□□□□□□□□□□□□□□□□□□□□□□□□□□□□□□□□□□□□□□□□□□□□□□□□□□□□□□□□□□□□□□□□□□□□□□□□□□□□□□□□□□□□□□□□□□□□□□□□□□□□□□□□□□□□□□□□□□□□□□□□□□□□□□□□□□□□□□□□□□□□□□□□□□□□□□□□□□□□□□□□□□□□□□□□□□□□□□□□□□□□□□□□□□□□□□□□□□□□□□□□□□□□□□□□□□□□□□□□□□□□□□□□□□□□□□□□□□□□□□□□□□□□□□□□□□□□□□□□□□□□□□□□□□□□□□□□□□□□□□□□□□□□□□□□□□□□□□□□□□□□□□□□□□□□□□□□□□□□□□□□□□□□□□□□□□□□□□□□□□□□□□□□□□□□□□□□□□□□□□□□□□□□□□□□□□□□□□□□□□□□□□T

AGTG□□□□□□□□□□□□□□□□□□□□□□□□□□□□□□□□□□□□□□□□□□□□□□□□□□□□□□□□□□□□□□□□□□□□□□□□□□□□□□□□□□□□□□□□□□□□□□□□□□□□□□□□□□□□□□□□□□□□□□□□□□□□□□□□□□□□□□□□□□□□□□□□□□□□□□□□□□□□□□□□□□□□□□□□□□□□□□□□□□□□□□□□□□□□□□□□□□□□□□□□□□□□□□□□□□□□□□□□□□□□□□□□□□□□□□□□□□□□□□□□□□□□□□□□□□□□□□□□□□□□□□□□□□□□□□□□□□□□□□□□□□□□□□□□□□□□□□□□□□□□□□□□□□□□□□□□□□□□□□□□□□□□□□□□□□□□□□□□□□□□□□□□□□□□□□□□□□□□□□□□□□□□□□□□□□□□□□□□□□□□□□□□□□□□□□□□□□□□□□□□□□□□□□□□□□□□□□□□□□□□□□□□□□□□□□□TT

AGTG□□□□□□□□□□□□□□□□□□□□□□□□□□□□□□□□□□□□□□□□□□□□□□□□□□□□□□□□□□□□□□□□□□□□□□□□□□□□□□□□□□□□□□□□□□□□□□□□□□□□□□□□□□□□□□□□□□□□□□□□□□□□□□□□□□□□□□□□□□□□□□□□□□□□□□□□□□□□□□□□□□□□□□□□□□□□□□□□□□□□□□□□□□□□□□□□□□□□□□□□□□□□□□□□□□□□□□□□□□□□□□□□□□□□□□□□□□□□□□□□□□□□□□□□□□□□□□□□□□□□□□□□□□□□□□□□□□□□□□□□□□□□□□□□□□□□□□□□□□□□□□□□□□□□□□□□□□□□□□□□□□□□□□□□□□□□□□□□□□□□□□□□□□□□□□□□□□□□□□□□□□□□□□□□□□□□□□□□□□□□□□□□□□□□□□□□□□□□□□□□□□□□□□□□□□□□□□□□□□□□□□□□□□□□□□□□□□□□□□□□□□□□□□□□□□□□□□□□□□□□□□□□□□□□□□□□□□□□□□□□□□□□□□□□□□□□□□□□□□□□□□□□□□□□□□□□□□□□□□□□□□□□□□□□□□□□□□□□□□□□□□□□□□□□□□□□□□□□□□□□□□□□□□□□□TT

ALT-like clone

AGTG□□□□□□□□□□□□□□□□□□□□□□□□□□□□□□□□□□□□□□□□□□□□□□□□T

AGTG□□□□□□□□□□□□□□□□□□□□□□□□□□□□□□□□□□□□□□□□□□□□□□□□□□□□□□□□□□□□□□□□□□□□□□□□□□□□□□□□□□□□□□□□□□□□□□□□□□□□□□□□□□□□□□□□□□□□□□□□□□□□□□□□□□□□□□□□□□□□□□□□□□□□□□□□□□□□□□□□□□□□□□□□□□□□□□□□□□□□□□□□□□□□□□□□□□□□□□□□□□□□□□□□□□□□□□□□□□□□□□□□□□□□□□□□□□□□□□□□□□□□□□□□□□□□□□□□□□□□□□□□□□□□□□□□□□□□□□□□□□□□□□□□□□□□□□□□□□□□□□□□□□□□□□□□□□□□□□□□□□□□□□□□□□□□□□□□□□□□□□□□□□□□□□□□□□□□□□□□□□□□□□□□□□□□□□□□□□□□□□□□□□□□□□□□□□□□□□□□□□□□□□□□□□□□□□□□□□□□□□□□□□□□□□□□□□□□□□□□□□□□□□□□□□□□□□□□□□□□□□□□□□□□□□□□□□□□□□TTAG

AGTG□□□□□□□□□□□□□□□□□□□□□□□□□□□□□□□□□□□□□□□□□□□□□□□□□□□□□□□□□□□□□□□□□□□□□□□□□□□□□□□□□□□□□□□□□□□□□□□□□□□□□□□□□□□□□□□□□□□□□□□□□□□□□□□□□□□□□□□□□□□□□□TT

AGTG□□□□□□□□□□□□□□□□□□□□□□□□□□□□□□□□□□□□□□□□□□□□□□□□□□□□□□□□□□□□□□□□□□□□□□□□□□□□□□□□□□□□□□□□□□□□□□□□□□□□□□□□□□□□□□□□□□□□□□□□□□□□□□□□□□□□□□□□□□□□□□□□□□□□TT

AGTG□□□□□□□□□□□□□□□□□□□□□□□□□□□□□□□□□□□□□□□□□□□□□□□□□□□□□□□□□□□□□□□□□□□□□□□□□□□□□□□□□□□□□□□□T

AGTG□□□□□□□□□□□□□□□□□□□□□□□□□□□□□□□□□□□□□□□□□□□□□□□□□□□□□□□□□□□□□□□□□□□□□□□□□□□□□□□□□□□□□□□□□□□□□□□□□□□□□□□□□□□□□□□□□□□□□□□□□□□□□□□□□□□□□□□□□□□□□□□□□□□□□□□□□□□□□□□□□□□□□□□□□□□□□□□□□□□□□□□□□□□□□□□□□□□□□□□□□□□□□□□□□□□□□□□□□□□□□□□□□□□□□□□□□□□□□□□□□□□□□□□□□□□□□□□□□□□□□□□□□□□□□□□□□□□□□□□□□□□□□□□□□□□□□□□□□□□□□□□□□□□□□□□□□□□□□□□□□□□□□□□□□□□□□□□□□□□□□□□□□□□□□□□□□□□□□□□□□□□□□□□□□□□□□□□□□□□□□□□□□□□□□□□□□□□□□□□□□□□□□□□□□□□□□□□□□□□□□□□□□□□□□□□□□□□□□□□□□□□□□□□□□□□□□□□□□□□□□□□□□□□□□□□□□□□□□□□□□□□□□□□□□□□□□□□□□□□□□□□□□T

AGTG□□□□□□□□□□□□□□□□□□□□□□□□□□□□□□□□□□□□□□□□□□□□□□□□□□□□□□□□□□□□□□□□□□□□□□□□□□□□□□□□□□□□□□□□□□□□□□□□□□□□□□□□□□□□□□□□□□□□□□□□□□□□□□□□□□□□□□□□□□□□□□□□□□□□□□□□□□□□□□□□□□□□□□□□□□□□□□□□□□□□□□□□□□□□□□□□□□□□□□□□□□□□□□□□□□□□□□□□□□□□□□□□□□□□□□□□□□□□□□□□□□□□□□□□□□□□□□□□□□□□□□□□□□□□□□□□□□□□□□□□□□□□□□□□□□□□□□□□□□□□□□□□□□□□□□□□□□□□□□□□□□□□□□□□□□□□□□□□□□□□□□G□□□□□□□□□□□□□□□□□□□□□□□□□□□□□□□□□□□□□□□□□□□□□□□□□□□□□□□□□□□□□□□□□□□□□□□□□□□□□□□□□□□□□□□□□□□□□□□□□□□□□□□□□□□□□□□□□□□□□□□□□□□□□□□□□□□□□□□□□□□□□□□□□□□□□□□□□□□□□□□□□□□□□□□□□□□□□□□□□□□□□□□□□□□□□□□□□TGAG

AGTG□□□□□□□□□□□□□□□□□□□□□□□□□□□□□□□□□□□□□□□□□□□□□□□□□□□□□□□□□□□□□□□□□□□□□□□□□□□□□□□□□□□□□□□□□□□□□□□□□□□□□□□□□□□□□□□□□□□□□□□□□□□□□□□□□□□□□□□□□□□□□□□□□□□□□□□□□□□□□□□□□□□□□□□□□□□□□□□□□□□□□□□□□□□□□□□□□□□□□□□□□□□□□□□□□□□□□□□□□□□□□□□□□□□□□□□□□□□□□A

AGTG□□□□□□□□□□□□□□□□□□□□□□□□□□□□□□□□□□□□□□□□□□□□□□□□□□□□□□□□□□□□□□□□□□□□□□□□□□□□□□□□□□□□□□□□□□□□□□□□□□□□□□□□□□□□□□□□□□□□□□□□□□□□□□□□□□□□□□□□□□□□□□G□□□□□□□□□□□□□□□□□□□□□□□□□□□□□□□□□□□□□□□□□□□□□□□□□□□□□□□□□□□□□□□□□□□□□□□□□□□□□□□□□□□□□□□□□□□□□□□□□□□□TT

AGTG□□□□□□□□□□□□□□□□□□□□□□□□□□□□□□□□□□□□□□□□□□□□□□□□□□G□□□□□□□□□□□□□□□□□□□□□□□□□□□□□□□□□□□□□□□□□□□□□□□□□□□□□□□□□□□□□□□□□□□□□□□□□□□□□□□□□□□□□□□□□□□□□□□□□□□

AGTG□□□□□□□□□□□□□□□□□□□□□□□□□□□□□□□□□□□□□□□□□□□□□□□□□□□□□□□□□□□□□□□□□□□□□□□□□□□□□□□□□□□□□□□□□□□□□□□□□□□□□□□□□□□□□□□□□□□□□□□□□□□□□□□□□□□□□□□□□□□□□□□□□□□TT

AGTG□□□□□□□□□□□□□□□□□□□□□□□□□□□□□□□□□□□□□□□□□□□□□□□□□□□□□□□□□□□□□□□□□□□□□□□□□□□□□□□□□□□□□□□□□□□□□□□□□□□□□□□□□□□□□□□□□□□□□□□□□□□□□□□□□□□□□□□□□□□□□□□□□□□□□□□□□□□□□□□□□□□□□□□□□□□□□□□□□□□□□□□□□□□□□□□□□□□□□□□□□□□□□□□□□□□□□TT

AGTG□□□□□□□□□□□□□□□□□□□□□□□□□□□□□□□□□□□□□□□□□□□□□□□□□□□□□□□□□□□□□□□□□□□□□□□□□□□□□□□□□□□□□□□□□□□□□□□□□□□□□□□□□□□□□□□□□□□□□□□□□□□□□□□□□□□□□□□□□□□□□□□□□□□□□□□□□□□□□□□□□□□□□□□□□□□□□□□□□□□□□□□□□□□□□□□□□□□□□□□□□□□□□□□□□□□□□□□□□□□□□□□□□□□□□□□□□□□□□□□□□□□□□□□□□□□□□□□□□□□□□□□□□□□□□□□□□□□□□□□□□□□□□□□□□□□□□□□□□□□□□□□□□□□□□□□□□□□□□□□□□□□□□□□□□□□□□□□□□□□□□□□□□□□□□□□□□□□□□□□□□□□□□□□□□□□□□□□□□□□□□□□□□□□□□□□□□□□□□□□□□□□□□□□□□□□□□□□□□□□□□□□□□□□□□□□□□□□□□□□□□□□□□□□□□□□□□TT

AGTG□□□□□□□□□□□□□□□□□□□□□□□□□□□□□□□□□□□□□□□□□□□□□□□□□□□□□□□□□□□□□□□□□□□□□□□□□□□□□□□□□□□□□□□□□□□□□□□□□□□□□□□□□□□□□□□□□□□□□□□□□□□□□□□□□□□□□□□□□□□□□□□□□□□□□□□□□□□□□□□□□□□□□□□□□□□□□□□□□□□□□□□□□□□□□□□□□□□□□□□□□□□□□□□□□□□□□□□□□□□□□□□□□□□□□□□□□□□□□□□□□□□□□□□□□□□□□□□□□□□□□□□□□□□□□□□□□□□□□□□□□□□□□□□□□□□□□□□□□□□□□□□□□□□□□□□□□□□□□□□□□□□□□□□□□□□□□□□□□□□□□□□□□□□□□□□□□□□□□□□□□□□□□□□□□□□□□□□□□□□□□□□□□□□□□□□□□□□□□□□□□□□□□□□□□□□□□□□□□□□□□□□□□□□□□□□□□□□□□□□□□□□□□□□□□□□□□□□□□□□□□□□□□□□□□□□□□□□□□□□□□□□□□□□□□□□□□□□□□□□□□□□□□□□□□□□□□□□□□□□□□□□□□□□□□□□□□□□□□□□□□□□□□□□□□□□□□□□□□□□□□□□□□□□□□□□□□□□□□□TT

**HCA2**

XpYp telomere allele 1

Parental

GAGGG□□□□□□□□□□□□□□□□□□□□□□□□□□T

GAGGG□□□□□□□□□□□□□□□□□□□□□□□□□□□□□□□□□□□□□□□□□□□□□□□□□□□□□□□□□□□□□□□□□□□□□□□□□□□□□□□□□TT

GAGGG□□□□□□□□□□□□□□□□□□□□□□□□□□□□□□□□□□□□□□□□□□□□□□□□□□□□□□□□□□□□□□□□□□□□□□□□□□□□□□□□□□□□□□□□□□□□□□□□□□□□□□□TT

GAGGG□□□□□□□□□□□□□□□□□□□□□□□□□□□□□□□□□□□□□□□□□□□□□□□□□□□□□□□□□□□□□□□□□□□□□□□□□□□□□□□□□□□□□□□□□□□□□□□□□□□□□□□□□□□□□□□□□□□□□□□□□□□□TT

GAGGG□□□□□□□□□□□□□□□□□□□□□□□□□□□□□□□□□□□□□□□□□□□□□□□□□□□□□□□□□□□□□□□□□□□□□□□□□□□□□□□□□□□□□□□□□□□□□□□□□□□□□□□□□□□□□□□□□□□□□□□□□□□□□□□□□□□□□□□□□□□□□□□□□□□□□□□□□□□□□□□□□□□□□□□TT

GAGGG□□□□□□□□□□□□□□□□□□□□□□□□□□□□□□□□□□□□□□□□□□□□□□□□□□□□□□□□□□□□□□□□□□□□□□□□□□□□□□□□□□□□□□□□□□□□□□□□□□□□□□□□□□□□□□□□□□□□□□□□□□□□□□□□□□□□□□□□□□□□□□□□□□□□□□□□□□□□□□□□□□□□□□□□□□□□□□□□□□□□□□□□□□□□□□□□□□□□□□□□□□□□□□TT

GAGGG□□□□□□□□□□□□□□□□□□□□□□□□□□□□□□□□□□□□□□□□□□□□□□□□□□□□□□□□□□□□□□□□□□□□□□□□□□□□□□□□□□□□□□□□□□□□□□□□□□□□□□□□□□□□□□□□□□□□□□□□□□□□□□□□□□□□□□□□□□□□□□□□□□□□□□□□□□□□□□□□□□□□□□□□□□□□□□□□□□□□□□□□□□□□□□□□□□□□□□□□□□□□□□□□□□□□□□□□□□□□□□□□□□□□□□□□□□□□□□□□□□□□□□□□□□□□□□□□□□□□□□□□□□□□□□□□□□□□□□□□□□□TT

GAGGG□□□□□□□□□□□□□□□□□□□□□□□□□□□□□□□□□□□□□□□□□□□□□□□□□□□□□□□□□□□□□□□□□□□□□□□□□□□□□□□□□□□□□□□□□□□□□□□□□□□□□□□□□□□□□□□□□□□□□□□□□□□□□□□□□□□□□□□□□□□□□□□□□□□□□□□□□□□□□□□□□□□□□□□□□□□□□□□□□□□□□□□□□□□□□□□□□□□□□□□□□□□□□□□□□□□□□□□□□□□□□□□□□□□□□□□□□□□□□□□□□□□□□□□□□□□□□□□□□□□□□□□□□□□□□□□□□□□□□□□□□□□□□□□□□□□□□□□□□□□□□□□□□□□□□□□□□□□□□□□□□□□□□□□□□□□□□□□□□□□□□□□□□□□□□□□□□□□□□□□□□□□□□□□□□□□□□□□□□□□□□□□□□□□□□□□□□□□□□□□□□□□□□□□□□□□□□□□□□□□□□□□□□□□□□□□□□□□□□□□□□□□□□□□□□□□□□□□□□□□□□□□□□□□□□□□□□□□□□□□□□□□□□□□□□□□□□□□□□□□□□□□□□□□□□□□□□□□□□□□□□□□□□□□□□□□□□□□□□□□□□□□□□□□□□□□□□□□□□□□□□□□□□□□□□□□□□□□□□□□□□□□□□□□□□□□□□□□□□□□□□□□□□□□□□□□□□□□□□□□□□□□□□□□□□□□□□□□□□□□□□□□□□□□□□□□□□□□□□□□□□□□□□□□□□□□□□□□□□□□□□□□□□□□□□□□□□□□□□□□□□□□□□□□□□□□□□□□□□□□□□□□□□□□□□□□□□□□□□□□□□□□□□□□□□□□□□□□□□□□□□□□□□□□□□□□□□□□□□□□□□□□□□□□□□□□□□□□□□□□□□□□□□□□□□□□□□□□□□□□□□□□□□□□□□□□□□□□□□□□□□□□□□□□□□□□□□□□□□□□□□□□□□□□□□□□□□□□□□□□□□□□□□□□□□□□□□□□□□□□□□□□□□□□□□□□□□□□□□□□□□□□□□□□□□□□□□□□□□□□□□□□□□□□□□□□□□□□□□□□□□□□□□□□□□□□□□□□□□□□□□□□□□□□□□□□□□□□□□□□□□□□□□□□□□□□□□□□□□□□□□□□□□□□□□□□□□□□□□□□□□□□□□□□□□□□□□□□□□□□□□□□□□□□□□□□□□□□□□□□□□□□□□□□□□□□□□□□□□□□□□□□□□□□□□□□□□□□□□□□□□□□□□□□□□□□□□□□□□□□□□□□□□□□□□□□□□□□□□□□□□□□□□□□□□□□□□□□□□□□□□□□□□□□□□□□□□□□□□□□□□□□□□□□□□□□□□□□□□□□□□□□□□□□□□□□□□□□□□□□□□□□□□□□□□□□□□□□□□□□□□□□□□□□□□□□□□□□□□□□□□□□□□□□□□□□□□□□□□□□□□□□□□□□□□□□□□□□□□□□□□□□□□□□□□□□□□□□□□□□□□□□□□□□□□□□□□□□□□□□□□□□□□□□□□□□□□□□□□□□□□□□□□□□□□□□□□□□□□□□□□□□□□□□□□□□□□□□□□□□□□□□□□□□□□□□□□□□□□□□□□□□□□□□□□□□□T

ALT clone

GAGGG□□□□□□□□□□□□□□□□□□□□□□□□□□□TT

GAGGG□□□□□□□□□□□□□□□□□□□□□□□□□□□□□□□□□□□□□□□□□□□□□□□□□□□□□□□□TT

GAGGG□□□□□□□□□□□□□□□□□□□□□□□□□□□□□□□□□□□□□□□□□□□□□□□□□□□□□□□□□□□□□□□□□□□□□□□□□□□□□□□□□□□□TT

GAGGG□□□□□□□□□□□□□□□□□□□□□□□□□□□□□□□□□□□□□□□□□□□□□□□□□□□□□□□□□□□□□□□□□□□□□□□□□□□□□□□□□□□□□□□□□□□□□□□□□□□□□□□□□□□□□□□□□□□□□□□□□□□□□□□□□□□□□□□□□□□□□□□□□□□□□□□□□□□□□□□□□□□□□□□□□□□□□□□□□□□□□□□□□□□□□□□□□□□TAGGG□□□□□□□□□□□□□□□□□□□□□□□□□□□□□□□□□□□□□□□□□□□□□□□□□□□□□□□□□□□□□□□□□□□□□□□□□□□□□□□□□□□□□□□□□□□□□□□□□□□□□□□□□□□□□□□□□□□□□□□□□□□□□□□□□□□□□□□□□□□□□□□□□□□□□□□□□□□□□□□□□□□□□□□□□□□□□□□□□□□□□□□G□□□□□□□□□□□□□□□□□□□□□□□□□□□□□□□□□□□□□□□TT

GAGGG□□□□□□□□□□□□□□□□□□□□□□□□□□□□□□□□□□□□□□□□□□□□□□□□□□□□□□□□□□□□□□□□□□□□□TT

GAGGG□□□□□□□□□□□□□□□□□□□□□□□□□□□□□□□□□□□□□□□□□□□□□□□□□□□□□□□□□□□□□□□□□□□□□□□□□□□T

GAGGG□□□□□□□□□□□□□□□□□□□□□□□□□□□□□□□□□□□□□□□□□□□□□□□□□□□□□□□□□□□□□□□□□□□□□□□□□□□TT

GAGGG□□□□□□□□□□□□□□□□□□□□□□□□□□□TAGGG□G□G□GT□□□TAGGG□G□G□GT□□□□□□□□□□□□□□□□□□□□□□□□□□□□□□□□□□□□□□□□□□□□□□□□□□TT

GAGGG□□□□□□□□□□□□□□□□□□□□□□□□□□□□□□□□□□□□□□□□□□□□□□□□□□□□□□□□□□□□□□□□□□□□□□□□□□□□□□□□□□□□□□□□□□□□□□□□□□□TT

GAGGG□□□□□□□□□□□□□□□□□□□□□□□□□□□□□□□□□□□□□□□□□□□□□□□□□□□□□□□□□□□□□□□□□□□□□□□□□□□□□□□□□□□□□□□□□□□□□□□□□□□□□□□TT

GAGGG□□□□□□□□□□□□□□□□□□□□□□□□□□□□□□□□□□□□□□□□□□□□□□□□□□□□□□□□□□□□□□□□□□□□□□□□□□□□□□□□□□□□□□□□□□□□□□□□□□□□□□□□□□□□□□□□□□□□□□□□□TT

GAGGG□□□□□□□□□□□□□□□□□□□□□□□□□□□□□□□□□□□□□□□□□□□□□□□□□□□□□□□□□□□□□□□□□□□□□□□□□□□□□□□□□□□□□□□□□□□□□□□□□□□□□□□□□□□□□□□□□□□□□□□□□□□□□□□□□□□□□□□□□□□□□□□□□□□□□□□□□TG

GAGGG□□□□□□□□□□□□□□□□□□□□□□□□□□□□□□□□□□□□□□□□□□□□□□□□□□□□□□□□□□□□□□□□□□□□□□□□□□□□□□□□□□□□□□□□□□□□□□□□□□□□□□□□□□□□□□□□□□□□□□□□□□□□□□□□□□□□□□□□□□□□□□□□□□□□□□□□□□□□□□□□□□□□□□□□□□□□□□□□□□□□□□□□□□□□□□□□□□□TT

GAGGG□□□□□□□□□□□□□□□□□□□□□□□□□□□□□□□□□□□□□□□□□□□□□□□□□□□□□□□□□□□□□□□□□□□□□□□□□□□□□□□□□□□□□□□□□□□□□□□□□□□□□□□□□□□□□□□□□□□□□□□□□□□□□□□□□□□□□□□□□□□□□□□□□□□□□□□□□□□□□□□□□□□□□□□□□□□□□□□□□□□□□□□□□□□□□□□□□□□□□□□□□□□□□□□TT

GAGGG□□□□□□□□□□□□□□□□□□□□□□□□□□□□□□□□□□□□□□□□□□□□□□□□□□□□□□□□□□□□□□□□□□□□□□□□□□□□□□□□□□□□□□□□□□□□□□□□□□□□□□□□□□□□□□□□□□□□□□□□□□□□□□□□□□□□□□□□□□□□□□□□□□□□□□□□□□□□□□□□□□□□□□□□□□□□□□□□□□□□□□□□□□□□□□□□□□□□□□□□□□□□□□□□□□□□□□□□TT

GAGGG□□□□□□□□□□□□□□□□□□□□□□□□□□□□□□□□□□□□□□□□□□□□□□□□□□□□□□□□□□□□□□□□□□□□□□□□□□□□□□□□□□□□□□□□□□□□□□□□□□□□□□□□□□□□□□□□□□□□□□□□□□□□□□□□□□□□□□□□□□□□□□□□□□□□□□□□□□□□□□□□□□□□□□□□□□□□□□□□□□□□□□□□□□□□□□□□□□□□□□□□□□□□□□□□□□□□□□□□□□□□□□TT

GAGGG□□□□□□□□□□□□□□□□□□□□□□□□□□□□□□□□□□□□□□□□□□□□□□□□□□□□□□□□□□□□□□□□□□□□□□□□□□□□□□□□□□□□□□□□□□□□□□□□□□□□□□□□□□□□□□□□□□□□□□□□□□□□□□□□□□□□□□□□□□□□□□□□□□□□□□□□□□□□□□□□□□□□□□□□□□□□□□□□□□□□□□□□□□□□□□□□□□□□□□□□□□□□□□□□□□□□□□□□□□□□□□□□□□□□□TT

GAGGG□□□□□□□□□□□□□□□□□□□□□□□□□□□□□□□□□□□□□□□□□□□□□□□□□□□□□□□□□□□□□□□□□□□□□□□□□□□□□□□□□□□□□□G□□□□□□□□□□□□□□□□□□□□□□□□□□□□□□□□□□□□□□□□□□□□□□□□□□□□□□□□□□□□□□□□□□□□□□□□□□□□□□□□□□□□□□□□□□□□□□□□□□□□□□□G□□□□□□□□□□□□□□□□□□□□□□□□□□□□□□□□□□□□□□□□□□□□□□□□□□□□□□□□□□□□□□□□□□□□□□□□□□□TT

GAGGG□□□□□□□□□□□□□□□□□□□□□□□□□□□□□□□□□□□□□□□□□□□□□□□□□□□□□□□□□□□□□□□□□□□□□□□□□□□□□□□□□□□□□□□□□□□□□□□□□□□□□□□□□□□□□□□□□□□□□□□□□□□□□□□□□□□□□□□□□□□□□□□□□□□□□□□□□□□□□□□□□□□□□□□□□□□□□□□□□□□□□□□□□□□□□□□□□□□□□□□□□□□□□□□□□□□□□□□□□□□□□□□□□□□□□□□□□□□□□□□□□□□□□□□□□□□□□□□□□□□□□□□□□□□□□□□□□□□□□□□□□□□□□□□□□□□□□□□□□□□□□□□□□□□□□□□□□□□□□□□□□□□□□□TT

GAGGG□□□□□□□□□□□□□□□□□□□□□□□□□□□□□□□□□□□□□□□□□□□□□□□□□□□□□□□□□□□□□□□□□□□□□□□□□□□□□□□□□□□□□□□□□□□□□□□□□□□□□□□□□□□□□□□□□□□□□□□□□□□□□□□□□□□□□□□□□□□□□□□□□□□□□□□□□□□□□□□□□□□□□□□□□□□□□□□□□□□□□□□□□□□□□□□□□□□□□□□□□□□□□□□□□□□□□□□□□□□□□□□□□□□□□□□□□□□□□□□□□□□□□□□□□□□□□□□□□□□□□□□□□□□□□□□□□□□□□□□□□□□□□□□□□□□□□□□□□□□□□□□□□□□□□□□□□□□□□□□□□□□□□□□□□□□□□□□□□□□□□□□□□□□□□□□□□□□□□□□□□□□□□□□□□□□□□□□□□□□□□□□□□□□□□□□□□□□□□□□□□□□□□□□□□□□□□□□□□□□□□□□□□□□□□□□□□□□□□□□□□□□□□□□□□□□□□□□□□□□□□□□□□□□□□□□□□□□□□□□□□□□□□□□□□□□□□□□□□□□□□□□□□□□□□□□□□□□□□□□□□□□□□□□□□□□□□□□□□□□□□□□□□□□□□□□□□□□□□□□□□□□□□□□□□□□□□TT

XpYp telomere allele 2

Parental

GAGGG□□□□□□□□□□□□□□□□□□□T

GAGGG□□□□□□□□□□□□□□□□□□□□□□□□□□□□□□□□□□□□□□□□□□□□□□□□□□□□□□□□□□□□□□□□□□□□□TT

GAGGG□□□□□□□□□□□□□□□□□□□□□□□□□□□□□□□□□□□□□□□□□□□□□□□□□□□□□□□□□□□□□□□□□□□□□□□□□□□□□□□□□□□□□□□□□□□□□□□□□□□□□□□□□□□□□□□□□□□□□□□□□□□□□□□□□□□□□□□□□□□□□□□□□□□□□□□□□□□□□□□□□□□□□□□□□□□□□□TT

GAGGG□□□□□□□□□□□□□□□□□□□□□□□□□□□□□□□□□□□□□□□□□□□□□□□□□□□□□□□□□□□□□□□□□□□□□□□□□□□□□□□□□□□□□□□□□□□□□□□□□□□□□□□□□□□□□□□□□□□□□□□□□□□□□□□□□□□□□□□□□□□□□□□□□□□□□□□□□□□□□□□□□□□□□□□□□□□□□□□□□□□□□□□□□□□□□□□□□□□□□□□□□□□□□□□□□□□□□□□□□□□□□□□□□□□□□□□□□□□□□□□□□□□□□□□□□□□□□□□□TT

GAGGG□□□□□□□□□□□□□□□□□□□□□□□□□□□□□□□□□□□□□□□□□□□□□□□□□□□□□□□□□□□□□□□□□□□□□□□□□□□□□□□□□□□□□□□□□□□□□□□□□□□□□□□□□□□□□□□□□□□□□□□□□□□□□□□□□□□□□□□□□□□□□□□□□□□□□□□□□□□□□□□□□□□□□□□□□□□□□□□□□□□□□□□□□□□□□□□□□□□□□□□□□□□□□□□□□□□□□□□□□□□□□□□□□□□□□□□□□□□□□□□□□□□□□□□□□□□□□□□□□□□□□□□□□□□□□□□□□□□□□□□□□□□□□□□□□□□□□□□□□□□□TT

GAGGG□□□□□□□□□□□□□□□□□□□□□□□□□□□□□□□□□□□□□□□□□□□□□□□□□□□□□□□□□□□□□□□□□□□□□□□□□□□□□□□□□□□□□□□□□□□□□□□□□□□□□□□□□□□□□□□□□□□□□□□□□□□□□□□□□□□□□□□□□□□□□□□□□□□□□□□□□□□□□□□□□□□□□□□□□□□□□□□□□□□□□□□□□□□□□□□□□□□□□□□□□□□□□□□□□□□□□□□□□□□□□□□□□□□□□□□□□□□□□□□□□□□□□□□□□□□□□□□□□□□□□□□□□□□□□□□□□□□□□□□□□□□□□□□□□□□□□□□□□□□□□□□□□□□□□□□□□□□□□□□□□□□□TT

GAGGG□□□□□□□□□□□□□□□□□□□□□□□□□□□□□□□□□□□□□□□□□□□□□□□□□□□□□□□□□□□□□□□□□□□□□□□□□□□□□□□□□□□□□□□□□□□□□□□□□□□□□□□□□□□□□□□□□□□□□□□□□□□□□□□□□□□□□□□□□□□□□□□□□□□□□□□□□□□□□□□□□□□□□□□□□□□□□□□□□□□□□□□□□□□□□□□□□□□□□□□□□□□□□□□□□□□□□□□□□□□□□□□□□□□□□□□□□□□□□□□□□□□□□□□□□□□□□□□□□□□□□□□□□□□□□□□□□□□□□□□□□□□□□□□□□□□□□□□□□□□□□□□□□□□□□□□□□□□□□□□□□□□□□□□□□□□□□□□□□□□□□□□□□□□□□□□□□□□□□□□□□□□□□□□□TT

GAGGG□□□□□□□□□□□□□□□□□□□□□□□□□□□□□□□□□□□□□□□□□□□□□□□□□□□□□□□□□□□□□□□□□□□□□□□□□□□□□□□□□□□□□□□□□□□□□□□□□□□□□□□□□□□□□□□□□□□□□□□□□□□□□□□□□□□□□□□□□□□□□□□□□□□□□□□□□□□□□□□□□□□□□□□□□□□□□□□□□□□□□□□□□□□□□□□□□□□□□□□□□□□□□□□□□□□□□□□□□□□□□□□□□□□□□□□□□□□□□□□□□□□□□□□□□□□□□□□□□□□□□□□□□□□□□□□□□□□□□□□□□□□□□□□□□□□□□□□□□□□□□□□□□□□□□□□□□□□□□□□□□□□□□□□□□□□□□□□□□□□□□□□□□□□□□□□□□□□□□□□□□□□□□□□□□□□□□□□□□□□□□□□□□□□□□□□□□□□□□□□□□□□□□□□□□□□□□TT

GAGGG□□□□□□□□□□□□□□□□□□□□□□□□□□□□□□□□□□□□□□□□□□□□□□□□□□□□□□□□□□□□□□□□□□□□□□□□□□□□□□□□□□□□□□□□□□□□□□□□□□□□□□□□□□□□□□□□□□□□□□□□□□□□□□□□□□□□□□□□□□□□□□□□□□□□□□□□□□□□□□□□□□□□□□□□□□□□□□□□□□□□□□□□□□□□□□□□□□□□□□□□□□□□□□□□□□□□□□□□□□□□□□□□□□□□□□□□□□□□□□□□□□□□□□□□□□□□□□□□□□□□□□□□□□□□□□□□□□□□□□□□□□□□□□□□□□□□□□□□□□□□□□□□□□□□□□□□□□□□□□□□□□□□□□□□□□□□□□□□□□□□□□□□□□□□□□□□□□□□□□□□□□□□□□□□□□□□□□□□□□□□□□□□□□□□□□□□□□□□□□□□□□□□□□□□□□□□□□□□□□□□□□□□□□□□□□□□□□□□□□□□□□□□□□□□□□□□□□□□□□□□□□□□□□□□□□□□□□□□□□□□□□□□□□□□□□□□□□□□□□□□□□□□□□□□□□□□□□□□□□□□□□□□□□□□□□□□□□□TT

ALT clone

GAGGG□□□□□□□□□□□□□□□□TC

GAGGG□□□□□□□□□□□□□□□□□□□□□□□□□□□□□□□□□□□□□□□□□□□□□□TT

GAGGG□□□□□□□□□□□□□□□□□□□□□□□□□□□□□□□□□□□□□□□□□□□□□□□□□□□□□□□□□□□□□□□□□□□□□□□□□□□□TT

GAGGG□□□□□□□□□□□□□□□□□□□□□□□□□□□□□□□□□□□□□□□□□□□□□□□□□□□□□□□□□□□□□□□□□□□□□□□□□□□□□□□□□□□□□□□□□□□□□□□□□□□□□□□□□□TG

GAGGG□□□□□□□□□□v□□□□□□□□□□□□□□□□□□□□□□□□□□□□□□□□□□□□□□□□□□□□□□□□□□□□□□□□□□□□□□□□□□□□□□□□□□□□□□□□□□□□□□□□□□□□□□□□□□□□□□□□□□□□□TT

GAGGG□□□□□□□□□□□□□□□□□□□□□□□□□□□□□□□□□□□□□□□□□□□□□□□□□□□□□□□□□□□□□□□□□□□□□□□□□□□□□□□□□□□□□□□□□□□□□□□□□□□□□□□□□□□□□□□□□□□□□□□□□T

GAGGG□□□□□□□□□□□□□□□□□□□□□□□□□□□□□□□□□□□□□□□□□□□□□□□□□□□□□□□□□□□□□□□□□□□□□□□□□□□□□□□□□□□□□□□□□□□□□□□□□□□□□□□□□□□□□□□□□□□□□□G□□□□□□T□□GTTAA

GAGGG□□□□□□□□□□□□□□□□□□□□□□□□□□□□□□□□□□□□□□□□□□□□□□□□□□□□□□□□□□□□□□□□□□□□□□□□□□□□□□□□□□□□□□□□□□□□□□□□□□□□□□□□□□□□□□□□□□□□□□□□□□□□□□□□TT

GAGGG□□□□□□□□□□□□□□□□□□□□□□□□□□□□□□□□□□□□□□□□□□□□□□□□□□□□□□□□□□□□□□□□□□□□□□□□□□□□□□□□□□□□□□□□□□□□□□□□□□□□□□□□□□□□□□□□□□□□□□□□□□□□□□□□□□□□□□□□□□□□□□□□□□□□□□□□TTA

GAGGG□□□□□□□□□□□□□□□□□□□□□□□□□□□□□□□□□□□□□□□□□□□□□□□□□□□□□□□□□□□□□□□□□□□□□□□□□□□□□□□□□□□□□□□□□□□□□□□□□□□□□□□□□□□□□□□□□□□□□□□□□□□□□□□□□□□□□□□□□□□□□□□□□□□□□□□□□□TT

GAGGG□□□□□□□□□□□□□□□□□□□□□□□□□□□□□□□□□□□□□□□□□□□□□□□□□□□□□□□□□□□□□□□□□□□□□□□□□□□□□□□□□□□□□□□□□□□□□□□□□□□□□□□□□□□□□□□□□□□□□□□□□□□□□□□□□□□□□□□□□□□□□□□□□□□□□□□□□□□□□□□□□□□□□□□□□□TT

GAGGG□□□□□□□□□□□□□□□□□□□□□□□□□□□□□□□□□□□□□□□□□□□□□□□□□□□□□□□□□□□□□□□□□□□□□□□□□□□□□□□□□□□□□□□□□□□□□□□□□□□□□□□□□□□□□□□□□□□□□□□□□□□□□□□□□□□□□□□□□□□□□□□□□□□□□□□□□□□□□□□□□□□□□□□□□□□□□□□□□□□□□□□TT

GAGGG□□□□□□□□□□□□□□□□□□□□□□□□□□□□□□□□□□□□□□□□□□□□□□□□□□□□□□□□□□□□□□□□□□□□□□□□□□□□□□□□□□□□□□□□□□□□□□□□□□□□□□□□□□□□□□□□□□□□□□□□□□□□□□□□□□□□□□□□□□□□□□□□□□□□□□□□□□□□□□□□□□□□□□□□□□□□□□□□□□□□□□□□□□□□□□□□□□□□□□□□□□□□□TT

GAGGG□□□□□□□□□□□□□□□□□□□□□□□□□□□□□□□□□□□□□□□□□□□□□□□□□□□□□□□□□□□□□□□□□□□□□□□□□□□□□□□□□□□□□□□□□□□□□□□□□□□□□□□□□□□□□□□□□□□□□□□□□□□□□□□□□□□□□□□□□□□□□□□□□□□□□□□□□□□□□□□□□□□□□□□□□□□□□□□□□□□□□□□□□□□□□□□□□□□□□□□□□□□□□□□□TG

GAGGG□□□□□□□□□□□□□□□□□□□□□□□□□□□□□□□□□□□□□□□□□□□□□□□□□□□□□□□□□□□□□□□□□□□□□□□□□□□□□□□□□□□□□□□□□□□□□□□□□□□□□□□□□□□□□□□□□□□□□□□□□□□□□□□□□□□□□□□□□□□□□□□□□□□□□□□□□□□□□□□□□□□□□□□□□□□□□□□□□□□□□□□□□□□□□□□□□□□□□□□□□□□□□□T□□□□□□□□□□□□□TT

GAGGG□□□□□□□□□□□□□□□□□□□□□□□□□□□□□□□□□□□□□□□□□□□□□□□□□□□□□□□□□□□□□□□□□□□□□□□□□□□□□□□□□□□□□□□□□□□□□□□□□□□□□□□□□□□□□□□□□□□□□□□□□□□□□□□□□□□□□□□□□□□□□□□□□□□□□□□□□□□□□□□□□□□□□□□□□□□□□□□□□□□□□□□□□□□□□□□□□□□□□□□□□□□□□□□□□□□□□□□□□□□□□□□□□□□□□□□□□□□T

GAGGG□□□□□□□□□□□□□□□□□□□□□□□□□□□□□□□□□□□□□□□□□□□□□□□□□□□□□□□□□□□□□□□□□□□□□□□□□□□□□□□□□□□□□□□□□□□□□□□□□□□□□□□□□□□□□□□□□□□□□□□□□□□□□□□□□□□□□□□□□□□□□□□□□□□□□□□□□□□□□□□□□□□□□□□□□□□□□□□□□□□□□□□□□□□□□□□□□□□□□□□□□□□□□□□□□□□□□□□□□□□□□□□□□□□□□□□□□□□□□□□□□□□□□□□□TT

GAGGG□□□□□□□□□□□□□□□□□□□□□□□□□□□□□□□□□□□□□□□□□□□□□□□□□□□□□□□□□□□□□□□□□□□□□□□□□□□□□□□□□□□□□□□□□□□□□□□□□□□□□□□□□□□□□□□□□□□□□□□□□□□□□□□□□□□□□□□□□□□□□□□□□□□□□□□□□□□□□□□□□□□□□□□□□□□□□□□□□□□□□□□□□□□□□□□□□□□□□□□□□□□□□□□□□□□□□□□□□□□□□□□□□□□□□□□□□□□□□□□□□□□□□□□□□□□□□□□□□□□□□□□□□□□□□□□□□□□□□□□□□□□□□□□□□□□□□□□□□□□□□□□□□TT

GAGGG□□□□□□□□□□□□□□□□□□□□□□□□□□□□□□□□□□□□□□□□□□□□□□□□□□□□□□□□□□□□□□□□□□□□□□□□□□□□□□□□□□□□□□□□□□□□□□□□□□□□□□□□□□□□□□□□□□□□□□□□□□□□□□□□□□□□□□□□□□□□□□□□□□□□□□□□□□□□□□□□□□□□□□□□□□□□□□□□□□□□□□□□□□□□□□□□□□□□□□□□□□□□□□□□□□□□□□□□□□□□□□□□□□□□□□□□□□□□□□□□□□□□□□□□□□□□□□□□□□□□□□□□□□□□□□□□□□□□□□□□□□□□□□□□□□□□□□□□□□□□□□□□□□□□□□□□□□□□□□□□□□□□□□□□□□□□□□□□□□□□□□□□□□□□□□□□□□□□□□□□□□□□□□□□□□□□□□□□□□□□□□□□□□□□□□□TT

GAGGG□□□□□□□□□□□□□□□□□□□□□□□□□□□□□□□□□□□□□□□□□□□□□□□□□□□□□□□□□□□□□□□□□□□□□□□□□□□□□□□□□□□□□□□□□□□□□□□□□□□□□□□□□□□□□□□□□□□□□□□□□□□□□□□□□□□□□□□□□□□□□□□□□□□□□□□□□□□□□□□□□□□□□□□□□□□□□□□□□□□□□□□□□□□□□□□□□□□□□□□□□□□□□□□□□□□□□□□□□□□□□□□□□□□□□□□□□□□□□□□□□□□□□□□□□□□□□□□□□□□□□□□□□□□□□□□□□□□□□□□□□□□□□□□□□□□□□□□□□□□□□□□□□□□□□□□□□□□□□□□□□□□□□□□□□□□□□□□□□□□□□□□□□□□□□□□□□□□□□□□□□□□□□□□□□□□□□□□□□□□□□□□□□□□□□□□□□□□□□□□□□□□□□□□□□□□□□□□□□□□□□□□□□□□□□□□□□□□□□□□□□□□□□□□□□□□□□□□□□□□□□□□□□□□□□□□□□□□□□□□□□□□□□□□□□□□□□□□□□□□□□□□□□□□□□□□□□□□□□□□□□□□□□□□□□□□□□□□□□□□□□□□□□□□□□□□□□□□□□□□□□□□□□□□□□□□□□□□□□□□□□□□□□□□□□□□□□□□□□□□□□□□□□□□□□□□□□□□□□□□□□□□□□□□□□□□□□□□□□□□□□□□□□□□□□□□□□□□□□□□□□□□□□□□□□□□□□□□□□□□□□□□□□□□□□□□□□□□□□□□□□□□□□□□□□□□□□□□□□□□□□□□□□□□□□□□□□□□□□□□□□□□□□□□□□□□□□□□□□□□□□□□□□□□□□□□□□□□□□□□□□□□□□□□□□□□□□□□□□□□□□□□□□□□□□□□□□□□□□□□□□□□□□□□□□□□□□□□□□□□□□□□□□□□□□□□□□□□□□□□□□□□□□□□□□□□□□□□□□□□□□□□□□□□□□□□□□□□□□□□□□□□□□□□□□□□□□□□□□□□□□□□□□□□□□□□□□□□□□□□□□□□□□□□□□□□□□□□□□□□□□□□□□□□□□□□□□□□□□□□□□□□□□□□□□□□□□□□□□□□□□□□□□□□□□□□□□□□□□□□□□□□□□□□□□□□□□□□□□□□□□□□□□□□□□□□□□□□□□□□□□□□□□□□□□□□□□□□□□□□□□□□□□□□□□□□□□□□□□□□□□□□□□□□□□□□□□□□□□□□□□□□□□□□□□□□□□□□□□□□□□□□□□□□□□□□□□□□□□□T

17p telomere allele 1

Parental

AAGGG□□□□□□□□□□□□□□□□□□□□□□□□□□□□□□□□□□□□□□□□□□□□□□□□□□□□□□□□TT

AAGGG□□□□□□□□□□□□□□□□□□□□□□□□□□□□□□□□□□□□□□□□□□□□□□□□□□□□□□□□□□□□□□□□□□□□□□□□□□□□□□□□□□□□□□□□□□□□□□□□□□□□□□□□□□□□□□□□□□□□□□□□□□□□□□□□□□□□□□□□□□TT

AAGGG□□□□□□□□□□□□□□□□□□□□□□□□□□□□□□□□□□□□□□□□□□□□□□□□□□□□□□□□□□□□□□□□□□□□□□□□□□□□□□□□□□□□□□□□□□□□□□□□□□□□□□□□□□□□□□□□□□□□□□□□□□□□□□□□□□□□□□□□□□□□□□□□□□□□□□□□□□□□□□□□□□□□□□TT

AAGGG□□□□□□□□□□□□□□□□□□□□□□□□□□□□□□□□□□□□□□□□□□□□□□□□□□□□□□□□□□□□□□□□□□□□□□□□□□□□□□□□□□□□□□□□□□□□□□□□□□□□□□□□□□□□□□□□□□□□□□□□□□□□□□□□□□□□□□□□□□□□□□□□□□□□□□□□□□□□□□□□□□□□□□□□□□□□□□□□□□□□□□□□□□□□□□□□□□□□□□□□□□□□□T

AAGGG□□□□□□□□□□□□□□□□□□□□□□□□□□□□□□□□□□□□□□□□□□□□□□□□□□□□□□□□□□□□□□□□□□□□□□□□□□□□□□□□□□□□□□□□□□□□□□□□□□□□□□□□□□□□□□□□□□□□□□□□□□□□□□□□□□□□□□□□□□□□□□□□□□□□□□□□□□□□□□□□□□□□□□□□□□□□□□□□□□□□□□□□□□□□□□□□□□□□□□□□□□□□□□□□□□□□□□□□□□□□□□□□□□□□□□□□□□□□□□□□□□□□□TT

AAGGG□□□□□□□□□□□□□□□□□□□□□□□□□□□□□□□□□□□□□□□□□□□□□□□□□□□□□□□□□□□□□□□□□□□□□□□□□□□□□□□□□□□□□□□□□□□□□□□□□□□□□□□□□□□□□□□□□□□□□□□□□□□□□□□□□□□□□□□□□□□□□□□□□□□□□□□□□□□□□□□□□□□□□□□□□□□□□□□□□□□□□□□□□□□□□□□□□□□□□□□□□□□□□□□□□□□□□□□□□□□□□□□□□□□□□□□□□□□□□□□□□□□□□□□□□□□□□□□□□□□□□□□□□□□□□□□□□□□□□□□□□□□□□TT

AAGGG□□□□□□□□□□□□□□□□□□□□□□□□□□□□□□□□□□□□□□□□□□□□□□□□□□□□□□□□□□□□□□□□□□□□□□□□□□□□□□□□□□□□□□□□□□□□□□□□□□□□□□□□□□□□□□□□□□□□□□□□□□□□□□□□□□□□□□□□□□□□□□□□□□□□□□□□□□□□□□□□□□□□□□□□□□□□□□□□□□□□□□□□□□□□□□□□□□□□□□□□□□□□□□□□□□□□□□□□□□□□□□□□□□□□□□□□□□□□□□□□□□□□□□□□□□□□□□□□□□□□□□□□□□□□□□□□□□□□□□□□□□□□□□□□□□□□□□□□□□□□□□□□□□□□□□□□□□□□□□□□□□□□□□□□□□□□TT

AAGGG□□□□□□□□□□□□□□□□□□□□□□□□□□□□□□□□□□□□□□□□□□□□□□□□□□□□□□□□□□□□□□□□□□□□□□□□□□□□□□□□□□□□□□□□□□□□□□□□□□□□□□□□□□□□□□□□□□□□□□□□□□□□□□□□□□□□□□□□□□□□□□□□□□□□□□□□□□□□□□□□□□□□□□□□□□□□□□□□□□□□□□□□□□□□□□□□□□□□□□□□□□□□□□□□□□□□□□□□□□□□□□□□□□□□□□□□□□□□□□□□□□□□□□□□□□□□□□□□□□□□□□□□□□□□□□□□□□□□□□□□□□□□□□□□□□□□□□□□□□□□□□□□□□□□□□□□□□□□□□□□□□□□□□□□□□□□□□□□□□□□□□□□□□□□□□□□□□□□□□□□□□□□□□□□□□□□□□□□□□□□□□□□□□□□□□□□□□□□□□□□□□□□□□□□□□□□□□□□□□□□□□□□□□□□□□□□□□□□□□□□□□□□□□□□□□□□□□□□□□□□□□□□□□□□□□□□□□□□□□□□□□□□□□□□□□□□□□□□□□□□□□□□□□□□□□□□□□□□□□□□□□□□□□□□□□□□□□□□□□□□□□□□□□□□□□□□□□□□□□□□□□□□□□□□□□□□□□□□□□□□□□□□□□□□□□□□□□□□□□□□□□□□□□□□□□□□□□□□□□□□□□□□□□□□□□□□□□□□□□□□□□□□□□□□□□□□□□□□□□□□□□□□□□□□□□□□□□□□□□□□□□□□□□□□□□□□□□□□□□□□□□□□□□□□□□□□□□□□□□□□□□□□□□□□□□□□□□□□□□□□□□□□□□□□□□□□□□□□□□□□□□□□□□□□□□□□□□□□□□□□□□□□□□□□□□□□□□□□□□□□□□□□□□□□□□□□□□□□□□□□□□□□□□□□□□□□□□□□□□□□□□□□□□□□□□□□□□□□□□□□□□□□□□□□□□□□□□□□□□□□□□□□□□□□□□□□□□□□□□□□□□□□□□□□□□□□□□□□□□□□□□□□□□□□□□□□□□□□□□□□□□□□□□□□□□□□□□□□□□□□□□□□□□□□□□□□□□□□□□□□□□□□□□□□□□□□□□□TT

ALT clone

AAGGG□□□□□□□□□□□□□□□□□□□□□□□□□TT

AAGGG□□□□□□□□□□□□□□□□□□□□□□□□□□□□□□□□□□□□□□□□□□□TTT

AAGGG□□□□□□□□□□□□□□□□□□□□□□□□□□□□□□□□□□□□□□□□□□□□□□□□□□□□□□□□□□□□□□□□□□□□□□□□□□□□□□□□□□□□□□□□□□□□□□□□□□□□□□□□□□□□□□□□□□□□□□□□□□□□□□□□□□□□□□□□□□□□□□□□□□□□□□□□□□□□□□□□□□□□□□□□□□□□□□□□□□□□□□□□□□□□□□□□□□□□□□□□□□□□□□□□□□□□□□□□□□□□□□□□□□□□□□□□□□□□□□□□□□□□□□□□□□□□□□□□□□□□□□□□□□□□□□□□□□□□□□□□□□□□□□□□□□□□□□□□□□□□□□□□□□□□□□□□□□□□□□□□□□□□□□□□□□□□□□□□□□□□□□□□□□□□□□□□□□□□□□□□□□□□□□□□□□□□□□□□□□□□□□□□□□□□□□□□□□□□□□□□□□□□□□□□□□□□□□□□□□□□□□□□□□□□□□□□□□□□□□□□□□□□□□□□□□□□□□□□□□TT

AAGGG□□□□□□□□□□□□□□□□□□□□□□□□□□□□□□□□□□□□□□□□□□□□□□□□□□□□□□□□□□□□□□□□□□TT

AAGGG□□□□□□□□□□□□□□□□□□□□□□□□□□□□□□□□□□□□□□□□□□□□□□□□□□□□□□□□□□□□□□□□□□□□□□□□□□□□□□□□□□□□□□□□□□□□TT

AAGGG□□□□□□□□□□□□□□□□□□□□□□□□□□□□□□□□□□□□□□□□□□□□□□□□□□□□□□□□□□□□□□□□□□□□□□□□□□□□□□□□□□□□□□□□□□□□□□TT

AAGGG□□□□□□□□□□□□□□□□□□□□□□□□□□□□□□□□□□□□□□□□□□□□□□□□□□□□□□□□□□□□□□□□□□□□□□□□□□□□□□□□□□□□□□□□□□□□□□□□T

AAGGG□□□□□□□□□□□□□□□□□□□□□□□□□□□□□□□□□□□□□□□□□□□□□□□□□□□□□□□□□□□□□□□□□□□□□□□□□□□□□□□□□□□□□□□□□□□□□□□□□□□□□□TT

AAGGG□□□□□□□□□□□□□□□□□□□□□□□□□□□□□□□□□□□□□□□□□□□□□□□□□□□□□□□□□□□□□□□□□□□□□□□□□□□□□□□□□□□□□□□□□□□□□□□□□□□□□□□□□□TT

AAGGG□□□□□□□□□□□□□□□□□□□□□□□□□□□□□□□□□□□□□□□□□□□□□□□□□□□□□□□□□□□□□□□□□□□□□□□□□□□□□□□□□□□□□□□□□□□□□□□□□□□□□□□□□□□□□□□TT

AAGGG□□□□□□□□□□□□□□□□□□□□□□□□□□□□□□□□□□□□□□□□□□□□□□□□□□□□□□□□□□□□□□□□□□□□□□□□□□□□□□□□□□□□□□□□□□□□□□□□□□□□□□□□□□□□□□□□□□□□□□□□□□□□□□TT

AAGGG□□□□□□□□□□□□□□□□□□□□□□□□□□□□□□□□□□□□□□□□□□□□□□□□□□□□□□□□□□□□□□□□□□□□□□□□□□□□□□□□□□□□□□□□□□□□□□□□□□□□□□□□□□□□□□□□□□□□□□□□□□□□□□□□□□□□□□□□□□□□□□□TT

AAGGG□□□□□□□□□□□□□□□□□□□□□□□□□□□□□□□□□□□□□□□□□□□□□□□□□□□□□□□□□□□□□□□□□□□□□□□□□□□□□□□□□□□□□□□□□□□□□□□□□□□□□□□□□□□□□□□□□□□□□□□□□□□□□□□□□□□□□□□□□□□□□□□□

AAGGG□□□□□□□□□□□□□□□□□□□□□□□□□□□□□□□□□□□□□□□□□□□□□□□□□□□□□□□□□□□□□□□□□□□□□□□□□□□□□□□□□□□□□□□□□□□□□□□□□□□□□□□□□□□□□□□□□□□□□□□□□□□□□□□□□□□□□□□□□□□□□□□□TT

AAGGG□□□□□□□□□□□□□□□□□□□□□□□□□□□□□□□□□□□□□□□□□□□□□□□□□□□□□□□□□□□□□□□□□□□□□□□G□□□□□□□□□□□□□□□□□□□□□□□□□□□□□□□□□□□□□□□□□□□□□□□□□□□□□□□□□□□□□□□□□□□□□□□□□TT

AAGGG□□□□□□□□□□□□□□□□□□□□□□□□□□□□□□□□□□□□□□□□□□□□□□□□□□□□□□□□□□□□□□□□□□□□□□□□□□□□□□□□□□□□□□□□□□□□□□□□□□□□□□□□□□□□□□□□□□□□□□□□□□□□□□□□□□□□□□□□□□□□□□□□□□TT

AAGGG□□□□□□□□□□□□□□□□□□□□□□□□□□□□□□□□□□□□□□□□□□□□□□□□□□□□□□□□□□□□□□□□□□□□□□□□□□□□□□□□□□□□□□□□□□□□□□□□□□□□□□□□□□□□□□□□□□□□□□□□□□□□□□□□□□□□□□□□□□□□□□□□□□□□□□□□□□□□□□□□□□□□□□□□□□□□□□□□□□□□□□□□□□□□□□□□□□□□□□□□□□□□□□□□□□□T

AAGGG□□□□□□□□□□□□□□□□□□□□□□□□□□□□□□□□□□□□□□□□□□□□□□□□□□□□□□□□□□□□□□□□□□□□□□□□□□□□□□□□□□□□□□□□□□□□□□□□□□□□□□□□□□□□□□□□□□□□□□□□□□□□□□□□□□□□□□□□□□□□□□□□□□□□□□□□□□□□□□□□□□□□□□□□□□□□□□□□□□□□□□□□□□□□□□□□□□□□□□□□□□□□□□□□□□□□□□□□□□□□□□□□□□□□TT

AAGGG□□□□□□□□□□□□□□□□□□□□□□□□□□□□□□□□□□□□□□□□□□□□□□□□□□□□□□□□□□□□□□□□□□□□□□□□□□□□□□□□□□□□□□□□□□□□□□□□□□□□□□□□□□□□□□□□□□□□□□□□□□□□□□□□□□□□□□□□□□□□□□□□□□□□□□□□□□□□□□□□□□□□□□□□□□□□□□□□□□□□□□□□□□□□□□□□□□□□□□□□□□□□□□□□□□□□□□□□□□□□□□□□□□□□□□□□□□□□□□□□□□□□□□□□□□□□□□□□□□□□□□□□□□□□□□□□□□□□□□□□□□□□□□□□□□□□□□□□□□□□□□□□□□TT

AAGGG□□□□□□□□□□□□□□□□□□□□□□□□□□□□□□□□□□□□□□□□□□□□□□□□□□□□□□□□□□□□□□□□□□□□□□□□□□□□□□□□□□□□□□□□□□□□□□□□□□□□□□□□□□□□□□□□□□□□□□□□□□□□□□□□□□□□□□□□□□□□□□□□□□□□□□□□□□□□□□□□□□□□□□□□□□□□□□□□□□□□□□□□□□□□□□□□□□□□□□□□□□□□□□□□□□□□□□□□□□□□□□□□□□□□□□□□□□□□□□□□□□□□□□□□□□□□□□□□□□□□□□□□□□□□□□□□□□□□□□□□□□□□□□□□□□□□□□□□□□□□□□□□□□□□□□□□□□□□□□□□□□□□□□□□□□□□□□□□□□□□□□□□□□□□□□□□□□□□□□□□□□□□□□□□□□□□□□□□□□□□□□□□□□□□□□□□□□□□□□□□□□□□□□□□□□□□□□□□□□□□□□□□□□□□□□□□□□□□□□□□□□□□□□□□□□□□□□□□□□□□□□□□□□□□□□□□□□□□□□□□□□□□□□□□□□□□□□□□□□□□□□□□□□□□□□□□□□□□□□□□□□□□□□□□□□□□□□□□□□□□□□□□□□□□□□□□□□□□□□□□□□□□□□□□□□□□□□□□□□□□□□□□□□□□□□□□□□□□□□□□□□□□□□□□□□□□□□□□□□□□□□□□□□□□□□□□□□□□□□□□□□□□□□□□□□□□□□□□□□□□□□□□□□□□□□□□□□□□□□□□□□□□□□□□□□□□□□□□□□□□□□□□□□□□□□□□□□□□□□□□□□□□□□□□□□□□□□□□□□□□□□□□□□□□□□□□□□□□□□□□□□□□□□□□□□□□□□□□□□□□□□□□□□□□□□□□□□□□□□□□□□□□□□□□□□□□□□□□□□□□□□□□□□□□□□□□□□□□□□□□□□□□□□□□□□□□□□□□□□□□□□□□□□□□□□□□□□□□□□□□□□□□□□□□□□□□□□□□□□□□□□□□□□□□□□□□□□□□□□□□□□□□□□□□□□□□□□□□□□□□□□□□□□□□□□□□□□□□□□□□□□□□□□□□□□□□□□□□□□□□□□□□□□□□□□□□□□□□□□□□□□□□□□□□□□□□□□□□□□□□□□□□□□□□□□□□□□□□□□□□□□□□□□□TT

17p telomere allele 2

Parental

AGGGG□□□□□□□□□□□□□□□□□□□□TCA

AAGGG□□□□□□□□□□□□□□□□□□□□□□□□□□□□□□□□□□□□□□□□□□□□□□□□□□□□□□□□□□□□□□□□□□□□□□□□□□□□□□□□□□□□□□□□□□□□□□□□□□□□□□□□□□□□□□□□□□□□□□□□□□□□□□TT

AAGGG□□□□□□□□□□□□□□□□□□□□□□□□□□□□□□□□□□□□□□□□□□□□□□□□□□□□□□□□□□□□□□□□□□□□□□□□□□□□□□□□□□□□□□□□□□□□□□□□□□□□□□□□□□□□□□□□□□□□□□□□□□□□□□□□□□□□□□□□□□□□□□□□□□□□□□□□□□□□□□□□□□□□□□□□□□□□□□□□□□□□□□□□□□□□□□□□□□□□□□□□□□□□□□□□□□□□□□□□□□□□□□□□□□□□□□□□□□□□□□□□□□□□□□□□□□□□□□□□□□□□□□□□□□□□□□□□□□□□□□□□□□□□□□□□□□□□□□□□□□□□□□□□□□□□□□□□□□□□□□□□□□□□TT

AAGGG□□□□□□□□□□□□□□□□□□□□□□□□□□□□□□□□□□□□□□□□□□□□□□□□□□□□□□□□□□□□□□□□□□□□□□□□□□□□□□□□□□□□□□□□□□□□□□□□□□□□□□□□□□□□□□□□□□□□□□□□□□□□□□□□□□□□□□□□□□□□□□□□□□□□□□□□□□□□□□□□□□□□□□□□□□□□□□□□□□□□□□□□□□□□□□□□□□□□□□□□□□□□□□□□□□□□□□□□□□□□□□□□□□□□□□□□□□□□□□□□□□□□□□□□□□□□□□□□□□□□□□□□□□□□□□□□□□□□□□□□□□□□□□□□□□□□□□□□□□□□□□□□□□□□□□□□□□□□□□□□□□□□□□□□□□□□□□□□□□□□□□□□□□□□□□□□□□□□□□□□□□□□□□□□□□□□□□□□□□□□□□□□□□□□□□□□□□□□□□□□□□□□□□□□□□□□□□□□□□□□□□□□□□□□□□□□□□□□□□□□□□□□□□□□□□□□□□□□□□□□□□□□□□□□□□□□□□□□□□□□□□□□□□□□□□□□□□□□□□□□TT

AAGGG□□□□□□□□□□□□□□□□□□□□□□□□□□□□□□□□□□□□□□□□□□□□□□□□□□□□□□□□□□□□□□□□□□□□□□□□□□□□□□□□□□□□□□□□□□□□□□□□□□□□□□□□□□□□□□□□□□□□□□□□□□□□□□□□□□□□□□□□□□□□□□□□□□□□□□□□□□□□□□□□□□□□□□□□□□□□□□□□□□□□□□□□□□□□□□□□□□□□□□□□□□□□□□□□□□□□□□□□□□□□□□□□□□□□□□□□□□□□□□□□□□□□□□□□□□□□□□□□□□□□□□□□□□□□□□□□□□□□□□□□□□□□□□□□□□□□□□□□□□□□□□□□□□□□□□□□□□□□□□□□□□□□□□□□□□□□□□□□□□□□□□□□□□□□□□□□□□□□□□□□□□□□□□□□□□□□□□□□□□□□□□□□□□□□□□□□□□□□□□□□□□□□□□□□□□□□□□□□□□□□□□□□□□□□□□□□□□□□□□□□□□□□□□□□□□□□□□□□□□□□□□□□□□□□□□□□□□□□□□□□□□□□□□□□□□□□□□□□□□□□□□□□□□□□□□□□□□□□□□□□□□□□□□□□□□□□□□□□□□□□□□□□□□□□□□□□□□□□□□□□□□□□□□□□□□□□□□□□□□□□□□□□□□□□□□□□□□□□□□□□□□□□□□□□□□□□□□□□□□□□□□□□□□□□□□□□□□□□□□□□□TT

AAGGG□□□□□□□□□□□□□□□□□□□□□□□□□□□□□□□□□□□□□□□□□□□□□□□□□□□□□□□□□□□□□□□□□□□□□□□□□□□□□□□□□□□□□□□□□□□□□□□□□□□□□□□□□□□□□□□□□□□□□□□□□□□□□□□□□□□□□□□□□□□□□□□□□□□□□□□□□□□□□□□□□□□□□□□□□□□□□□□□□□□□□□□□□□□□□□□□□□□□□□□□□□□□□□□□□□□□□□□□□□□□□□□□□□□□□□□□□□□□□□□□□□□□□□□□□□□□□□□□□□□□□□□□□□□□□□□□□□□□□□□□□□□□□□□□□□□□□□□□□□□□□□□□□□□□□□□□□□□□□□□□□□□□□□□□□□□□□□□□□□□□□□□□□□□□□□□□□□□□□□□□□□□□□□□□□□□□□□□□□□□□□□□□□□□□□□□□□□□□□□□□□□□□□□□□□□□□□□□□□□□□□□□□□□□□□□□□□□□□□□□□□□□□□□□□□□□□□□□□□□□□□□□□□□□□□□□□□□□□□□□□□□□□□□□□□□□□□□□□□□□□□□□□□□□□□□□□□□□□□□□□□□□□□□□□□□□□□□□□□□□□□□□□□□□□□□□□□□□□□□□□□□□□□□□□□□□□□□□□□□□□□□□□□□□□□□□□□□□□□□□□□□□□□□□□□□□□□□□□□□□□□□□□□□□□□□□□□□□□□□□□□□□□□□□□□□□□□□□□□□□□□□□□□□□□□□□□□□□□□□□□□□□□□□□□□□□□□□□□□□□□□□□□□□□□□□□□□□□□□□□□□□□□□□□□□□□□□□□□□□□□□□□□□□□□□□□□□□□□□□□□□□□□□□□□□□□□□TT

AAGGG□□□□□□□□□□□□□□□□□□□□□□□□□□□□□□□□□□□□□□□□□□□□□□□□□□□□□□□□□□□□□□□□□□□□□□□□□□□□□□□□□□□□□□□□□□□□□□□□□□□□□□□□□□□□□□□□□□□□□□□□□□□□□□□□□□□□□□□□□□□□□□□□□□□□□□□□□□□□□□□□□□□□□□□□□□□□□□□□□□□□□□□□□□□□□□□□□□□□□□□□□□□□□□□□□□□□□□□□□□□□□□□□□□□□□□□□□□□□□□□□□□□□□□□□□□□□□□□□□□□□□□□□□□□□□□□□□□□□□□□□□□□□□□□□□□□□□□□□□□□□□□□□□□□□□□□□□□□□□□□□□□□□□□□□□□□□□□□□□□□□□□□□□□□□□□□□□□□□□□□□□□□□□□□□□□□□□□□□□□□□□□□□□□□□□□□□□□□□□□□□□□□□□□□□□□□□□□□□□□□□□□□□□□□□□□□□□□□□□□□□□□□□□□□□□□□□□□□□□□□□□□□□□□□□□□□□□□□□□□□□□□□□□□□□□□□□□□□□□□□□□□□□□□□□□□□□□□□□□□□□□□□□□□□□□□□□□□□□□□□□□□□□□□□□□□□□□□□□□□□□□□□□□□□□□□□□□□□□□□□□□□□□□□□□□□□□□□□□□□□□□□□□□□□□□□□□□□□□□□□□□□□□□□□□□□□□□□□□□□□□□□□□□□□□□□□□□□□□□□□□□□□□□□□□□□□□□□□□□□□□□□□□□□□□□□□□□□□□□□□□□□□□□□□□□□□□□□□□□□□□□□□□□□□□□□□□□□□□□□□□□□□□□□□□□□□□□□□□□□□□□□□□□□□□□□□□□□□□□□□□□□□□□□□□□□□□□□□□□□□□□□□□□□□□□□□□□□□□□□□□□□□□□□□□□□□□□□□□□□□□□□□□□□□□□□□□□□□□□□□□□□□□□□□□□□□□□□□□□□□□□□□□□□□□□□□□□□□□□□□□□□□□□□□□□TT

ALT clone

AAGGG□□□□□□□□□□□□□□□□□TT

AAGGG□□□□□□□□□□□□□□□□□□□□□□□□□□□□□□□□□□□□□□□□□□□□□□□□□□□□□□□□□□□□□□□□□□□□□□□□□□□□□□□□□TT

AAGGG□□□□□□□□□□□□□□□□□□□□□□□□□□□□□□□□□□□□□□□□□□□□□□□□□□□□□□□□□□□□□□□□□□□□□□□□□□□□□□□□□□□□□□□□□□□□□□□□□□□□□□□□□□□□□□□□□□□□□□□□□□□□□□□□□□□□□□□□□□□□□□□□□□□□□□□□□□□□□□□□□□□□□□□□□□□□□□□□□□□□□□□□□□□□□□□□□□□□□□□□□□□□□□□□□□□□□□□□□□□□□□□□□□□□□□□□□□□□□□□□□□□□□□□□□□□□□□□□□□□□□□□□□□□□□□□□□□□□□□□□□□□□□□□□□□□□□□□□□□□□□□□□□□□□□□□□□□□□□□□□□□□□□□□□□□□□□□□□□□□□□□□□□□□□□□□□□□□□□□□□□□□□□□□□□□□□□□□□□□□□□□□□□□□□□□□□□□□□□□□□□□□□□□□□□□□□□□□□□□□□□□□□□□□□□□□□□□□□□□□□□□□□□□□□□□□□□□□□□□□□□□□□□□□□□□□□□□□□□□□□□□□□□□□□□□□□□□□□□□□□□□□□□□□□□□□□□□□□□□□□□□□□□□□□□□□□□□□□□□□□□□□□□□□□□□□□□□□□□□□□□□□□□□□□□□□□□□□□□□□□□□□□□□□□□□□□□□□□□□□□□□□□□□□□□□□□□□□□□□□□□□□□□□□□□□□T□□□□□□□□□□□□□□□□□□□□□□□□□□□□□□□□□□□□□□□□□□□□□□□□□□□□□□□□□□□□□□□□□□□□□□□□□□□□□□□□□□□□□□□□□□□□□□□□□□□□□□□□□□□□□□□□□□□□□□□□□□□□□□□□□□□□□□□□□□□□□□□□□□□□□□□□□□□□□□□□□□□□□□□□□□□□□□□□□□□□□□□□□□□□□□□□□□□□□□□□□□□□□□□□□□□□□□□□□□□□□□□□□□□□□□□□□□□□□□□□□□□□□□□□□□□□□□□□□□□□□□□□□□□□□□□□□□□□□□□□□□□□□□□□□□□□□□□□□□□□□□□□T

AAGGG□□□□□□□□□□□□□□□□□□□□□□□□□□□□□□□□□□□□□□□□□□□□□□□□□TT

AAGGG□□□□□□□□□□□□□□□□□□□□□□□□□□□□□□□□□□□□□□□□□□□□□□□□□□□□□□□□□□□□□□□TT

AAGGG□□□□□□□□□□□□□□□□□□□□□□□□□□□□□□□□□□□□□□□□□□□□□□□□□□□□□□□□□□□□□□□□□□□□TT

AAGGG□□□□□□□□□□□□□□□□□□□□□□□□□□□□□□□□□□□□□□□□□□□□□□□□□□□□□□□□□□□□□□□□□□□□□□□□□□□□□□TT

AGGGG□□□□□□□□□□□□□□□□□□□□□□□□□□□□□□□□□□□□□□□□□□□□□□□□□□□□□□□□□□□□□□□□□□□□□□□□□□□□□□□□□□□□□T

AAGGG□□□□□□□□□□□□□□□□□□□□□□□□□□□□□□□□□□□□□□□□□□□□□□□□□□□□□□□□□□□□□□□□□□□□□□□□□□□□□□□□□□□□□□□□□□□TT

AAGGG□□□□□□□□□□□□□□□□□□□□□□□□□□□□□□□□□□□□□□□□□□□□□□□□□□□□□□□□□□□□□□□□□□□□□□□□□□□□□□□□□□□□□□□G□□□□□□□□□□□□□□TT

AAGGG□□□□□□□□□□□□□□□□□□□□□□□□□□□□□□□□□□□□□□□□□□□□□□□□□□□□□□□□□□□□□□□□□□□□□□□□□□□□□□□□□□□□□□□□□□□□□□□□□□□□□□□□□□TT

AAGGG□□□□□□□□□□□□□□□□□□□□□□□□□□□□□□□□□□□□□□□□□□□□□□□□□□□□□□□□□□□□□□□□□□□□□□□□□□□□□□□□□□G□□□□□□□□□□□□□□□□□□□□□□□□□□□□□□□□□□□□□□□□□□□□□TT

AAGGG□□□□□□□□□□□□□□□□□□□□□□□□□□□□□□□□□□□□□□□□□□□□□□□□□□□□□□□□□□□□□□□□□□□□□□□□□□□□□□□□□□□□□□□□□□□□□□□□□□□□□□□□□□□□□□□□□□□□□□□□□□□□□□□□□□□□□□□□TT

AAGGG□□□□□□□□□□□□□□□□□□□□□□□□□□□□□□□□□□□□□□□□□□□□□□□□□□□□□□□□□□□□□□□□□□□□□□□□□□□□□□□□□□□□□□□□□□□□□□□□□□□□□□□□□□□□□□□□□□□□□□□□□□□□□□□□□□□□□□□□□□□□□□□□□□□□TT

AAGGG□□□□□□□□□□□□□□□□□□□□□□□□□□□□□□□□□□□□□□□□□□□□□□□□□□□□□□□□□□□□□□□□□□□□□□□□□□□□□□□□□□□□□□□□□□□□□□□□□□□□□□□□□□□□□□□□□□□□□□□□□□□□□□□□□□□□□□□□□□□□□□□□□□□□□□□□□□□TT

AAGGG□□□□□□□□□□□□□□□□□□□□□□□□□□□□□□□□□□□□□□□□□□□□□□□□□□□□□□□□□□□□□□□□□□□□□□□□□□□□□□□□□□□□□□□□□□□□□□□□□□□□□□□□□□□□□□□□□□□□□□□□□□□□□□□□□□□□□□□□□□□□□□□□□□□□□□□□□□□□□□□□□□□□□□□□□□□□□□TT

AAGGG□□□□□□□□□□□□□□□□□□□□□□□□□□□□□□□□□□□□□□□□□□□□□□□□□□□□□□□□□□□□□□□□□□□□□□□□□□□□□□□□□□□□□□□□□□□□□□□□□□□□□□□□□□□□□□□□□□□□□□□□□□□□□□□□□□□□□□□□□□□□□□□□□□□□□□□□□□□□□□□□□□□□□□□□□□□□□□T

AAGGG□□□□□□□□□□□□□□□□□□□□□□□□□□□□□□□□□□□□□□□□□□□□□□□□□□□□□□□□□□□□□□□□□□□□□□□□□□□□□□□□□□□□□□□□□□□□□□□□□□□□□□□□□□□□□□□□□□□□□□□□□□□□□□□□□□□□□□□□□□□□□□□□□□□□□□□□□□□□□□□□□□□□□□□□□□□□□□□□□□□□□□□□□□□□□TT

AAGGG□□□□□□□□□□□□□□□□□□□□□□□□□□□□□□□□□□□□□□□□□□□□□□□□□□□□□□□□□□□□□□□□□□□□□□□□□□□□□□□□□□□□□□□□□□□□□□□□□□□□□□□□□□□□□□□□□□□□□□□□□□□□□□□□□□□□□□□□□□□□□□□□□□□□□□□□□□□□□□□□□□□□□□□□□□□□□□□□□□□□□□□□□□□□□□□□□□□□□□□□□□□□□□□□□□□□□□□□□□□□□□□□□TT

AAGGG□□□□□□□□□□□□□□□□□□□□□□□□□□□□□□□□□□□□□□□□□□□□□□□□□□□□□□□□□□□□□□□□□□□□□□□□□□□□□□□□□□□□□□□□□□□□□□□□□□□□□□□□□□□□□□□□□□□□□□□□□□□□□□□□□□□□□□□□□□□□□□□□□□□□□□□□□□□□□□□□□□□□□□□□□□□□□□□□□□□□□□□□□□□□□□□□□□□□□□□□□□□□□□□□□□□□□□□□□□□□□□□□□□□□□□□□□□□□□□□□□□□□□□□□□□□□□□□□□□□□□□□□□□□□□□□TT

AAGGG□□□□□□□□□□□□□□□□□□□□□□□□□□□□□□□□□□□□□□□□□□□□□□□□□□□□□□□□□□□□□□□□□□□□□□□□□□□□□□□□□□□□□□□□□□□□□□□□□□□□□□□□□□□□□□□□□□□□□□□□□□□□□□□□□□□□□□□□□□□□□□□□□□□□□□□□□□□□□□□□□□□□□□□□□□□□□□□□□□□□□□□□□□□□□□□□□□□□□□□□□□□□□□□□□□□□□□□□□□□□□□□□□□□□□□□□□□□□□□□□□□□□□□□□□□□□□□□□□□□□□□□□□□□□□□□□□□□□□□□□□□□□□□□□□□TT

AAGGG□□□□□□□□□□□□□□□□□□□□□□□□□□□□□□□□□□□□□□□□□□□□□□□□□□□□□□□□□□□□□□□□□□□□□□□□□□□□□□□□□□□□□□□□□□□□□□□□□□□□□□□□□□□□□□□□□□□□□□□□□□□□□□□□□□□□□□□□□□□□□□□□□□□□□□□□□□□□□□□□□□□□□□□□□□□□□□□□□□□□□□□□□□□□□□□□□□□□□□□□□□□□□□□□□□□□□□□□□□□□□□□□□□□□□□□□□□□□□□□□□□□□□□□□□□□□□□□□□□□□□□□□□□□□□□□□□□□□□□□□□□□□□□□□□□□□□□□□□□□□□□□□□□□□□□□□□□□□□□□□□□□□□□□□□□□□□□□□□□□□□□□□□□□□□□□□□□□□□□□□□□□□□□□□□□□□□□□□□□□□□□□□□□□□□□□□□□□□□□□□□□□□□□□□□□□□□□□□□□□□□□□□□□□□□□□□□□□□□□□□□□□□□□□□□□□□□□□□□□□□□□□□□□□□□□□□□□□□□□□□□□□□□□□□□□□□□□□□□□□□□□□□□□□□□□□□□□□□□□□□□□□□□□□□□□□□□□□□□□□□□□□□□□□□□□□□□□□□□□□□□□□□□□□□□□□□□□□□□□□□□□□□□□□□□□□□□□□□□□□□□□□□□□□□□□□□□□□□□□□□□□□□□□□□□□□□□□□□□□□□□□□□□□□□□□□□□□□□□□□□□□□□□□□□□□□□□□□□□□□□□□□□□□□□□□□□□□□□□□□□□□□□□□□□□□□□□□□□□□□□□□□□□□□□□□□□□□□□□□□□□□□□□□□□□□□□□□□□□□□□□□□□□□□□□□□□□□□□□□□□□□□□□□□□□□□□□□□□□□□□□□□□□□□□□□□□□□□□□□□□□□□□□□□□□□□□□□□□□□□□□□□□□□□□□□□□□□□□□□□□□□□□□□□□□□□□□□□□□□□□□□□□□□□□□□□□□□□□□□□□□□□□□□□□□□□□□□□□□□□□□□□□□□□□□□□□□□□□□□□□□□□□□□□□□□□□□□□□□□□□□□□□□□□□□□□□□□□□□□□□□□□□□□□□□□□□□□□□□□□□□□□□□□□□□□□□□□□□□□□□□□□□□□□□□□□□□□□□□□□□□□□□□□□□□□□□□□□□□□□□□□□□□□□□TT

**U2OS**

XpYp telomere

AGGG□□□□□□□□□□□□□□□□□□□□□□□□□□□□□□□□□□□□□□□□□□□□□□□□□□□□□□□□□□□□□□□□□□□□□□□□□□□□□□□□□□□□□□□□□□□□□□□□□TT

AGGG □□□□□□□□□□□□□□□□□□□□□□□□□□□□□□□□□□□□□□□□□□□□□□□□□□□□□□□□□□□□□□□□□□□□□□□□□□□□□□□□□□□□□□□□□□□□□□□□□□TT

AGGGG□□□□□□□□□□□□□□□□□□□□□□□□□□□□□□□□□□□□□□□□□□□□□□□□□□□□□□□□□□□□□□□□□□□□□□□□□□□□□□□□□□□□□□□□□□□□□□□□□□□□□□□□□□□□□□□□□□□□□□□□□□□□□□□□□□□□□□□□□□□□□TT

AGGG □□□□□□□□□□□□□□□□□□□□□□□□□□□□□□□□□□□□□□□□□□□□□□□□□□□□□□□□□□□□□□□□□□□□□□□□□□□□□□□□□□□□□□□□□□□□□□□□□□□□□□□□□□□□□□□□□□□□□□□□□□□□□□□□□□□□□□□□□□□□□□□□□□□□□□□□□□□□□□□□□□□□□□□□□□□□□□□□□□□□□□□□□□□□□□TT

AGGG□□□□□□□□□□□□□□□□□□□□□□□□□□□□□□□□□□□□□□□□□□□□□□□□□□□□□□□□□□□□□□□□□□□□□□□□□□□□□□□□□□□□□□□□□□□□□□□□□□□□□□□□□□□□□□□□□□□□□□□□□□□□□□□□□□□□□□□□□□□□□□□□□□□□□□□□□□□□□□□□□□□□□□□□□□□□□□□□□□□□□□□□□□□□□□□□□□□□□□□□□□□□□□□□□□□□□□□□□□□□□□TT

AGG□□□□□□□□□□□□□□□□□□□□□□□□□□□□□□□□□□□□□□□□□□□□□□□□□□□□□□□□□□□□□□□□□□□□□□□□□□□□□□□□□□□□□□□□□□□□□□□□□□□□□□□□□□□□□□□□□□□□□□□□□□□□□□□□□□□□□□□□□□□□□□□□□□□□□□□□□□□□□□□□□□□□□□□□□□□□□□□□□□□□□□□□□□□□□□□□□□□□□□□□□□□□□□□□□□□□□□□□□□□□□□□□□□□□□□□□□□□□□□□□□□□□□□□□□T

AGGG□□□□□□□□□□□□□□□□□□□□□□□□□□□□□□□□□□□□□□□□□□□□□□□□□□□□□□□□□□□□□□□□□□□□□□□□□□□□□□□□□□□□□□□□□□□□□□□□□□□□□□□□□□□□□□□□□□□□□□□□□□□□□□□□□□□□□□□□□□□□□□□□□□□□□□□□□□□□□□□□□□□□□□□□□□□□□□□□□□□□□□□□□□□□□□□□□□□□□□□□□□□□□□□□□□□□□□□□□□□□□□□□□□□□□□□□□□□□□□□□□□□□□□□□□□□□□□□□□□□□□□□□□□□□□□□□□□□□□□□□□□□□□□□□□TT

AGGG □□□□□□□□□□□□□□□□□□□□□□□□□□□□□□□□□□□□□□□□□□□□□□□□□□□□□□□□□□□□□□□□□□□□□□□□□□□□□□□□□□□□□□□□□□□□□□□□□□□□□□□□□□□□□□□□□□□□□□□□□□□□□□□□□□□□□□□□□□□□□□□□□□□□□□□□□□□□□□□□□□□□□□□□□□□□□□□□□□□□□□□□□□□□□□□□□□□□□□□□□□□□□□□□□□□□□□□□□□□□□□□□□□□□□□□□□□□□□□□□□□□□□□□□□□□□□□□□□□□□□□□□□□□□□□□□□□□□□□□□□□□□□□□□□□□□□□□□□□□□□□□□□□□□□□□T

AGGG□□□□□□□□□□□□□□□□□□□□□□□□□□□□□□□□□□□□□□□□□□□□□□□□□□□□□□□□□□□□□□□□□□□□□□□□□□□□□□□□□□□□□□□□□□□□□□□□□□□□□□□□□□□□□□□□□□□□□□□□□□□□□□□□□□□□□□□□□□□□□□□□□□□□□□□□□□□□□□□□□□□□□□□□□□□□□□□□□□□□□□□□□□□□□□□□□□□□□□□□□□□□□□□□□□□□□□□□□□□□□□□□□□□□□□□□□□□□□□□□□□□□□□□□□□□□□□□□□□□□□□□□□□□□□□□□□□□□□□□□□□□□□□□□□□□□□□□□□□□□□□□□□□□□□□□□□□□□□□□□□□□□□□□□□□□□□□□□□□□□□□□□□□□□□□□□□TT

AGGG□□□□□□□□□□□□□□□□□□□□□□□□□□□□□□□□□□□□□□□□□□□□□□□□□□□□□□□□□□□□□□□□□□□□□□□□□□□□□□□□□□□□□□□□□□□□□□□□□□□□□□□□□□□□□□□□□□□□□□□□□□□□□□□□□□□□□□□□□□□□□□□□□□□□□□□□□□□□□□□□□□□□□□□□□□□□□□□□□□□□□□□□□□□□□□□□□□□□□□□□□□□□□□□□□□□□□□□□□□□□□□□□□□□□□□□□□□□□□□□□□□□□□□□□□□□□□□□□□□□□□□□□□□□□□□□□□□□□□□□□□□□□□□□□□□□□□□□□□□□□□□□□□□□□□□□□□□□□□□□□□□□□□□□□□□□□□□□□□□□□□□□□□□□□□□□□□□□□□□□□□□□□□□□□□□□□T

AGGG□□□□□□□□□□□□□□□□□□□□□□□□□□□□□□□□□□□□□□□□□□□□□□□□□□□□□□□□□□□□□□□□□□□□□□□□□□□□□□□□□□□□□□□□□□□□□□□□□□□□□□□□□□□□□□□□□□□□□□□□□□□□□□□□□□□□□□□□□□□□□□□□□□□□□□□□□□□□□□□□□□□□□□□□□□□□□□□□□□□□□□□□□□□□□□□□□□□□□□□□□□□□□□□□□□□□□□□□□□□□□□□□□□□□□□□□□□□□□□□□□□□□□□□□□□□□□□□□□□□□□□□□□□□□□□□□□□□□□□□□□□□□□□□□□□□□□□□□□□□□□□□□□□□□□□□□□□□□□□□□□□□□□□□□□□□□□□□□□□□□□□□□□□□□□□□□□□□□□□□□□□□□□□□□□□□□TT

AGGG□□□□□□□□□□□□□□□□□□□□□□□□□□□□□□□□□□□□□□□□□□□□□□□□□□□□□□□□□□□□□□□□□□□□□□□□□□□□□□□□□□□□□□□□□□□□□□□□□□□□□□□□□□□□□□□□□□□□□□□□□□□□□□□□□□□□□□□□□□□□□□□□□□□□□□□□□□□□□□□□□□□□□□□□□□□□□□□□□□□□□□□□□□□□□□□□□□□□□□□□□□□□□□□□□□□□□□□□□□□□□□□□□□□□□□□□□□□□□□□□□□□□□□□□□□□□□□□□□□□□□□□□□□□□□□□□□□□□□□□□□□□□□□□□□□□□□□□□□□□□□□□□□□□□□□□□□□□□□□□□□□□□□□□□□□□□□□□□□□□□□□□□□□□□□□□□□□□□□□□□□□□□□□□□□□□□□□□□□□□□□□□□□□□□□□□□□□□□□□□□□□□□□□□□□□□□□□□□□□□□□□□□TT

AGGG□□□□□□□□□□□□□□□□□□□□□□□□□□□□□□□□□□□□□□□□□□□□□□□□□□□□□□□□□□□□□□□□□□□□□□□□□□□□□□□□□□□□□□□□□□□□□□□□□□□□□□□□□□□□□□□□□□□□□□□□□□□□□□□□□□□□□□□□□□□□□□□□□□□□□□□□□□□□□□□□□□□□□□□□□□□□□□□□□□□□□□□□□□□□□□□□□□□□□□□□□□□□□□□□□□□□□□□□□□□□□□□□□□□□□□□□□□□□□□□□□□□□□□□□□□□□□□□□□□□□□□□□□□□□□□□□□□□□□□□□□□□□□□□□□□□□□□□□□□□□□□□□□□□□□□□□□□□□□□□□□□□□□□□□□□□□□□□□□□□□□□□□□□□□□□□□□□□□□□□□□□□□□□□□□□□□□□□□□□□□□□□□□□□□□□□□□□□□□□□□□□□□□□□□□□□□□□□□□□□□□□□□□□□□□□□□□□□□□□□□□□□□□□□□□□□□□□□□□□□□□□□□□□□□□□□□□□□□□□□□□□□□□□□□□□□□□□□□□□□□□□□□□□□□□□□□□□□□□□□□□□□□□□TT

AGGG□□□□□□□□□□□□□□□□□□□□□□□□□□□□□□□□□□□□□□□□□□□□□□□□□□□□□□□□□□□□□□□□□□□□□□□□□□□□□□□□□□□□□□□□□□□□□□□□□□□□□□□□□□□□□□□□□□□□□□□□□□□□□□□□□□□□□□□□□□□□□□□□□□□□□□□□□□□□□□□□□□□□□□□□□□□□□□□□□□□□□□□□□□□□□□□□□□□□□□□□□□□□□□□□□□□□□□□□□□□□□□□□□□□□□□□□□□□□□□□□□□□□□□□□□□□□□□□□□□□□□□□□□□□□□□□□□□□□□□□□□□□□□□□□□□□□□□□□□□□□□□□□□□□□□□□□□□□□□□□□□□□□□□□□□□□□□□□□□□□□□□□□□□□□□□□□□□□□□□□□□□□□□□□□□□□□□□□□□□□□□□□□□□□□□□□□□□□□□□□□□□□□□□□□□□□□□□□□□□□□□□□□□□□□□□□□□□□□□□□□□□□□□□□□□□□□□□□□□□□□□□□□□□□□□□□□□□□□□□□□□□□□□□□□□□□□□□□□□□□□□□□□□□□□□□□□□□□□□□□□□□□□□□□□□□□□□□□□□□□□□□□□□□□□□□□□□□□□□□□□□□□□□□□□□□□□□□□□□□□□□□□□□□□□□□□□□□□□□□□□□□□□□□□□□□□□□□□□□□□□□□□□□□□□□□□□□□□□□□□□□□□□□□□□□□□□□□□□□□□□□□□□□□□□□□□□□□□□□□□□TT

17p telomere allele 1

AAGGG□□□□□□□□□□□□□□□□T

AAGGG□□□□□□□□□□□□□□□□□□□□□□□□□□□□TT

AAGGG□□□□□□□□□□□□□□□□□□□□□□□□□□□□□□□□□□□□□□□TTT

AAGGG□□□□□□□□□□□□□□□□□□□□□□□□□□□□□□□□□□□□□□□□□□□TTT

AAGGG□□□□□□□□□□□□□□□□□□□□□□□□□□□□□□□□□□□□□□□□□□□□□□□□□□□□□□□TT□

AAGGG□□□□□□□□□□□□□□□□□□□□□□□□□□□□□□□□□□□□□□□□□□□□□□□□□□□□□□□□□□TT

AAGGG□□□□□□□□□□□□□□□□□□□□□□□□□□□□□□□□□□□□□□□□□□□□□□□□□□□□□□□□□□□□□□□□TC

AAGGG□□□□□□□□□□□□□□□□□□□□□□□□□□□□□□□□□□□□□□□□□□□□□□□□□□□□□□□□□□□□□□□□□□□TCA

AAGGG□□□□□□□□□□□□□□□□□□□□□□□□□□□□□□□□□□□□□□□□□□□□□□□□□□□□□□□□□□□□□□□□□□□□□□□□□□□□□□□□□□□□□□□□□□□□□□□□□□□□□□□□□□□□□□□□□□□□□□□□□□□□□□□□□□□□□□□□□□□□□□□□□TT

AAGGG□□□□□□□□□□□□□□□□□□□□□□□□□□□□□□□□□□□□□□□□□□□□□□□□□□□□□□□□□□□□□□□□□□□□□□□□□□□□□□□□□□□□□□□□□□□□□□□□□□□□□□□□□□□□□□□□□□□□□□□□□□□□□□□□□□□□□□□□□□□□□□□□□□□□□□□□□□□□□□□□□□□□□□□□□□□□□□□□□□□□□□□□□□□□□□□□□□□□□□□□□□□□□□□□□□□□□□□□□□□□□□TT

AAGGG□□□□□□□□□□□□□□□□□□□□□□□□□□□□□□□□□□□□□□□□□□□□□□□□□□□□□□□□□□□□□□□□□□□□□□□□□□□□□□□□□□□□□□□□□□□□□□□□□□□□□□□□□□□□□□□□□□□□□□□□□□□□□□□□□□□□□□□□□□□□□□□□□□□□□□□□□□□□□□□□□□□□□□□□□□□□□□□□□□□□□□□□□□□□□□□□□□□□□□□□□□□□□□□□□□□□□□□□□□□□□□□□□□□□□□□□□□□□□□□□□□□□□□□□□□□□□□□□□□□□□□□□□□□□□□□□□□□□□□□□□□□□□□□□□□□□□□□□□□□□□□□□□□□□□□□□□□□□□□□□□□□□□□□□□□□□□□□□□□□□□□□□□□□□□□□□□□□□□□□□□□□□□□□□□□□□□□□□TT

AAGGG□□□□□□□□□□□□□□□□□□□□□□□□□□□□□□□□□□□□□□□□□□□□□□□□□□□□□□□□□□□□□□□□□□□□□□□□□□□□□□□□□□□□□□□□□□□□□□□□□□□□□□□□□□□□□□□□□□□□□□□□□□□□□□□□□□□□□□□□□□□□□□□□□□□□□□□□□□□□□□□□□□□□□□□□□□□□□□□□□□□□□□□□□□□□□□□□□□□□□□□□□□□□□□□□□□□□□□□□□□□□□□□□□□□□□□□□□□□□□□□□□□□□□□□□□□□□□□□□□□□□□□□□□□□□□□□□□□□□□□□□□□□□□□□□□□□□□□□□□□□□□□□□□□□□□□□□□□□□□□□□□□□□□□□□□□□□□□□□□□□□□□□□□□□□□□□□□□□□□□□□□□□□□□□□□□□□□□□□□□□□□□T

AAGGG□□□□□□□□□□□□□□□□□□□□□□□□□□□□□□□□□□□□□□□□□□□□□□□□□□□□□□□□□□□□□□□□□□□□□□□□□□□G□□□□□□□□□□□□□□□□□□□□□□□□□□□□□□□□□□□□□□□□□□□□□□□□□□□□□□□□□□□□□□□□□□□□□□□□□□□□□□□□□□□□□□□□□□□□□□□□□□□□□□□□□□□□□□□□□□□□□□□□□□□□□□□□□□□□□□□□□□□□□□□□□□□□□□□□□□□□□□□□□□□□□□□□□□□□□□□□□□□□□□□□□□□□□□□□□□□□□□□□□□□□□□□□□□□□□□□□□□□□□□□□□□□□□□□□□□□□□□□□□□□□□□□□□□□□□□□□□□□□□□□□□□□□□□□□□□□□□□□□□□□□□□□□□□□□□□□□□□□□□□□□□□□□□□□□□□□□□□□□□□□□□□□□□□□□□□□□□□□□□□□□□□□□□□□□□□□□□□□□□□□□□□□□□□□□□□□□TT

AAGGG□□□□□□□□□□□□□□□□□□□□□□□□□□□□□□□□□□□□□□□□□□□□□□□□□□□□□□□□□□□□□□□□□□□□□□□□□□□□□□□□□□□□□□□□□□□□□□□□□□□□□□□□□□□□□□□□□□□□□□□□□□□□□□□□□□□□□□□□□□□□□□□□□□□□□□□□□□□□□□□□□□□□□□□□□□□□□□□□□□□□□□□□□□□□□□□□□□□□□□□□□□□□□□□□□□□□□□□□□□□□□□□□□□□□□□□□□□□□□□□□□□□□□□□□□□□□□□□□□□□□□□□□□□□□□□□□□□□□□□□□□□□□□□□□□□□□□□□□□□□□□□□□□□□□□□□□□□□□□□□□□□□□□□□□□□□□□□□□□□□□□□□□□□□□□□□□□□□□□□□□□□□□□□□□□□□□□□□□□□□□□□□□□□□□□□□□□□□□□□□□□□□□□□□□□□□□□□□□□□□□□□□□□□□□□□□□□□□□□□□□□□□□□□□□□□□□□□□□□□□□□□□□□□□□□□□□□□□□□□□□□□□□□□□□□□□□□□□□□□□□□□□□□□□□□□□□□□□□□□□□□□□□□□□□□□□□□□□□□□□□□□□□□□□□□□□□□□□□□□□□□□□□□□□□□□□□□□□□□□□□□□□□□□□□□□□□□□□□□□□□□□□□□□□□□□□□□□□□□□□□□□□□□□□□□□□□□□□□□□□□□□□□□□□□□□□□□□□□□□□□□□□□□□□□□□□□□□□□□□□□□□□□□□□□□□□□□□□□□□□□□□□□□□□□□□□□□□□□□□□□□□□□□□□□□□□□□□□□□□□□□□□□□□□□□□□□□□□□□□□□□□□□□□□□□□□□□□□□□□□□□□□□□□□□□□□□□□□□□□□□□□□□□□□□□□□□□□□□□□□□□□□□□□□□□□□□□□□□□□□□□□□□□□□□□□□□□□□□□□□□□□□□□□□□□□□□□□□□□□□□□□□□□□□□□□□□□□□□□□□□□□□□□□□□□□□□□□□□□□□□□□□□□□□□□□□□□□□□□□□□□□□□□□□□□□□□□□□□□□T□□□□□□□□□□□□□□□□□□□□□□□□□□□□□□□□□□□□□□□□□□□□□□□□□□□□□□□□□□□□□□□□□□□□□□□□□□□□□□□□□□□□□□□□□□□□□□□□□□□□□□□□□□□□□□□□□□□□□□□□□□□□□□□□□□□□□□□□□□□□□□□□□□□□□□□□□□□□□□□□□□□□□□□□□□□□□□□□□□□□□□□□□□□□□□□□□□□□□□□□□□□□□□□□□□□□□□□□□□□□□□□□□□□□□□□□□□□□□□□□□□□□□□□□□□□□□□□□□□□□□□□□□□□□□□□□□□□□□□□□□□□□□□□□□□□□□□□□□□□□□□□□□□□□□□□□□□□□□□□□□□□□□□□□□□□□□□□□□□□□□□□□□□□□□□□□□□□□□□T□□□□□□□□□□□□□□□□□□□□□□□□□□□□□□□□□□□□□□□□□□□□□□□□□□□□□□□□□□□□□□□□□□□□□□□□□□□□□□□□□□□□□□□□□□□□□□□□□□□□□□□□□□□□□□□□□□□□□□□□□□□□□□□□□□□□□□□□□□□□□□□□□□□□□□□□□□□□□□□□□□□□□□□□□□□□□□□□□□□□□□□□□□□□□□□□□□□□□□□□□□□□□□□□□□□□□□□□□□□□□□□□□□□□□□□□□□□□□□□□□□□□□□□□□□□□□□□□□□□□□□□□□□□□□□□□□□□□□□□□□□□□□□□□□□□□□□□□□□□□□□□□□□□□□□□□□□□□□□□□□□□□□□□□□□□□□□□□□□□□□□□□□□□□□□□□□□□□□□□□□□□□□□□□□□□□□□□□□□□□□□□□□□□□□□□□G□□□□□□□□□□□□□□□□□□□□□□□□□□□□□□□□□□□□□□□□□□□□□□□□□□□□□□□□□□□□□□□□□□□□□□□□□□□□□□□□□□□□□□□□□□□□□□□□□□□□□□□□□□□□□□□□□□□□□□□□□□□□□□□□□□□□□□□□□□□□□□□□□□□□□□□□□□□□□□□□□□□□□□□□□□□□□□□□□□□□□□□□□□□□□□□□□□□□□□□□□□□□□□□□□□□□□□□□□□□□□□□□□□□□□□□□□□□□□□□□□□□□□□□□□□□□□□□□□□□□□□□□□□□□□□□□□□□□□□□□□□□□□□□□□□□□□□□□□□□□□□□□□□□□□□□□□□□□□□□□□□□□□□□□□□□□□□□□□□□□□□□□□□□□□□□□□□□□□□□□□□□□□□□□□□□□□□□□□□□□□□□□□□□□□□□□□□□□□□□□□□□□□□□□□□□□□□□□□□□□□□□□□□□□□□□□□□□□□□□□□□□□□□□□□□□□□□□□□□□□□□□□□□□□□□□□□□□□□□□□□□□□□□□□□□□□□TTA

AAGGG□□□□□□□□□□□□□□□□□□□□□□□□□□□□□□□□□□□□□□□□□TT

AAGGG□□□□□□□□□□□□□□□□□□□□□□□□□□□□□□□□□□□□□□□□□□□□□□□□□□TT

AAGGG□□□□□□□□□□□□□□□□□□□□□□□□□□□□□□□□□□□□□□□□□□□□□□□□□□□□□□□□□□□□□□□□□□□□□□□□□□□□□□□□□□□□□TT

AAGGG□□□□□□□□□□□□□□□□□□□□□□□□□□□□□□□□□□□□□□□□□□□□□□□□□□□□□□□□□□□□□□□□□□□□□□□□□□□□□□□□□□□□□□□□□□□□□□□□□□□□□□□□□□□□□□□□□□□□□□□□□□□□□□□□□□□□□□□□□□□□□□□□□□□□□□□□□□□□□□□□□□□□□□□□□□□□□□□□□□□□□□□□□□□□□□□□□TT

AAGGG□□□□□□□□□□□□□□□□□□□□□□□□□□□□□□□□□□□□□□□□□□□□□□□□□□□□□□□□□□□□□□□□□□□□□□□□□□□□□□□□□□□□□□□□□□□□□□□□□□□□□□□□□□□□□□□□□□□□□□□□□□□□□□□□□□□□□□□□□□□□□□□□□□□□□□□□□□□□□□□□□□□□□□□□□□□□□□□□□□□□□□□□□□□□□□□□□□□□□□□□□□□□□□□□□□□□□□□□□□□□□□□□□□□□□□□□□□□□□□□□□□□□□□□□□□□□□□□□□□□□□□□□□□□□TT

AAGGG□□□□□□□□□□□□□□□□□□□□□□□□□□□□□□□□□□□□□□□□□□□□□□□□□□□□□□□□□□□□□□□□□□□□□□□□□□□□□□□□□□□□□□□□□□□□□□□□□□□□□□□□□□□□□□□□□□□□□□□□□□□□□□□□□□□□□□□□□□□□□□□□□□□□□□□□□□□□□□□□□□□□□□□□□□□□□□□□□□□□□□□□□□□□□□□□□□□□□□□□□□□□□□□□□□□□□□□□□□□□□□□□□□□□□□□□□□□□□□□□□□□□□□□□□□□□□□□□□□□□□□□□□□□□□□□□□□□□□□□□□□□□□□□□□□□□□□□□□□□□□□□□□□□□□□□□□□□□□□□□□□□□□□□□□□□□□□□□□□□□□□□□□□□□□□□□□□□□□□□□□□□□□□□□□□□□□□□□□□□□□□□□□□□□□□□□□□□□□□□□□□□□□□□□□□□□□□□□□□□□□□□□□□□□□□□□□□□□□□□□□□□□□□□□□□□□□□□□□□□□□□□□□□□□□□□□□□□□□□□□□□□□□□□□□□□□□□□□□□□□□□□□□TT

AAGGG□□□□□□□□□□□□□□□□□□□□□□□□□□□□□□□□□□□□□□□□□□□□□□□□□□□□□□□□□□□□□□□□□□□□□□□□□□□□□□□□□□□□□□□□□□□□□□□□□□□□□□□□□□□□□□□□□□□□□□□□□□□□□□□□□□□□□□□□□□□□□□□□□□□□□□□□□□□□□□□□□□□□□□□□□□□□□□□□□□□□□□□□□□□□□□□□□□□□□□□□□□□□□□□□□□□□□□□□□□□□□□□□□□□□□□□□□□□□□□□□□□□□□□□□□□□□□□□□□□□□□□□□□□□□□□□□□□□□□□□□□□□□□□□□□□□□□□□□□□□□□□□□□□□□□□□□□□□□□□□□□□□□□□□□□□□□□□□□□□□□□□□□□□□□□□□□□□□□□□□□□□□□□□□□□□□□□□□□□□□□□□□□□□□□□□□□□□□□□□□□□□□□□□□□□□□□□□T□□□□□□□□□□□□□□□□□□□□□□□□□□□□□□□□□□□□□□□□□□□□□□□□□□□□□□□□□□□□□□□□□□□□□□□□□□□□□□□□□□□□□□□□□□□□□□□□□□□□□□□□□□□□□□□□□□□□□□□□□□□□□□□□□□□□□□□□□□□□□□□□□□□□□□□□□□□□□□□□□□□□□□□□□□□□□□□□□□□□□□□□□□□□□□□□□□□□□□□□□□□□□□□□□□□□□□□□□□□□□□□□□□□□□□□□□□□□□□□□□□□□□□□□□□□□□□□□□□□□□□□□□□□□□□□□□□□□□□□□□□□□□□□□□□□□□□□□□□□□□□□□□□□□□□□□□□□□□□□□□□□□□□□□□□□□□□□□□□□□□□□□□□□□□□□□□□□□□□□□□□□□□□□□□□□□□□□□□□□□□□□□□□□□□□□□□□□□□□□□□□□□□□□□□□□□□□□□□□□□□□□□□□□□□□□□□□□□□□□□□□□□□□□□□□□□□□□□□□□□□□□□□□□□□□□□□□□□□□□□□□□□□□□□□□□□□□□□□□□□□□□□□□□□□□□□□□□□□□□□□□□□□□□□□□□□□□□□□□□□□□□□□□□□□□□□□□□□□□□□□□□□□□□□□□□□□□□□□□□□□□□□□□□□□□□□□□□□□□□□□□□□□□□□□□□□□□□□□□□□□□□□□□□□□□□□□□□□□□□□□□□□□□□□□□□□□□□□□□□□□□□□□□□□□□□□□□□□□□□□□□□□□□□□□□□□□□□□□□□□□□□□□□□□□□□□□□□□□□□□□□□□□□□□□□□□□□□□□□□□□□□□□□□□□□□□□□□□□□□□□□□□□□□□□□□□□□□□□□□□□□□□□□□□□□□□□□□□□□□□□□□□□□□□□□□□□□□□□□□□□□□□□□□□□□□□□□□□□□□□□□□□□□□□□□□□□□□□□□□□□□□□□□□□□□□□□□□□□□□□□□□□□□□□□□□□□□□□□□□□□□□□□□□□□□□□□□□□□□□□□□□□□□□□□□□□□□□□□□□□□□□□□□□□□□□□□□□□□□□□□□□□□□□□□□□□□□□□□□□□□□□□□□□□□□□□□□□□□□□□□□□□□□□□□□□□□□□□□□□□□□□□□□□□□□□□□□□□G□□TT

17p telomere allele 2

AAGGG□□□□□□□□□□□□□□□□T

AAGGG□□□□□□□□□□□□□□□□□□□□□□□□□□□□□□□□□□□□□□□□TT

AAGGG□□□□□□□□□□□□□□□□□□□□□□□□□□□□□□□□□□□□□□□□□□□□□□□□□□□□□□□□□□□□□□□□□□□□□□□□□□□□TT

AAGGG□□□□□□□□□□□□□□□□□□□□□□□□□□□□□□□□□□□□□□□□□□□□□□□□□□□□□□□□□□□□□□□□□□□□□□□□□□□□□□□□□□□□□□□□□□□□TTAGG

AAGGG□□□□□□□□□□□□□□□□□□□□□□□□□□□□□□□□□□□□□□□□□□□□□□□□□□□□□□□□□□□□□□□□□□□□□□□□□□□□□□□□□□□□□□□□□□□□□□□□□□□□□□□□□□□□□□□□□TT

AAGGG□□□□□□□□□□□□□□□□□□□□□□□□□□□□□□□□□□□□□□□□□□□□□□□□□□□□□□□□□□□□□□□□□□□□□□□□□□□□□□□□□□□□□□□□□□□□□□□□□□□□□□□□□□□□□□□□□□□□□□□□□□□□□□□□□□□□□□□□□□□□□□□□□□TT

AAGGG□□□□□□□□□□□□□□□□□□□□□□□□□□□□□□□□□□□□□□□□□□□□□□□□□□□□□□□□□□□□□□□□□□□□□□□□□□□□□□□□□□□□□□□□□□□□□□□□□□□□□□□□□□□□□□□□□□□□□□□□□□□□□□□□□□□□□□□□□□□□□□□□□□□□□□TT

AAGGG□□□□□□□□□□□□□□□□□□□□□□□□□□□□□□□□□□□□□□□□□□□□□□□□□□□□□□□□□□□□□□□□□□□□□□□□□□□□□□□□□□□□□□□□□□□□□□□□□□□□□□□□□□□□□□□□□□□□□□□□□□□□□□□□□□□□□□□□□□□□□□□□□□□□□□□□□□□□□□□□□□□□□□□□□□□□□□□□□□□□□□□□□□□□□□□□□□□□□□□□□□□□□□□□□□□□□□□□□□□□□□□TT

AAGGG□□□□□□□□□□□□□□□□□□□□□□□□□□□□□□□□□□□□□□□□□□□□□□□□□□□□□□□□□□□□□□□□□□□□□□□□□□□□□□□□□□□□□□□□□□□□□□□□□□□□□□□□□□□□□□□□□□□□□□□□□□□□□□□□□□□□□□□□□□□□□□□□□□□□□□□□□□□□□□□□□□□□□□□□□□□□□□□□□□□□□□□□□□□□□□□□□□□□□□□□□□□□□□□□□□□□□□□□□□□□□□□□□□□□□□□□□□□□□□□□□□□□□□□□□□□□□□□□□□□□□□□□□TT

AAGGG□□□□□□□□□□□□□□□□□□□□□□□□□□□□□□□□□□□□□□□□□□□□□□□□□□□□□□□□□□□□□□□□□□□□□□□□□□□□□□□□□□□□□□□□□□□□□□□□□□□□□□□□□□□□□□□□□□□□□□□□□□□□□□□□□□□□□□□□□□□□□□□□□□□□□□□□□□□□□□□□□□□□□□□□□□□□□□□□□□□□□□□□□□□□□□□□□□□□□□□□□□□□□□□□□□□□□□□□□□□□□□□□□□□□□□□□□□□□□□□□□□□□□□□□□□□□□□□□□□□□□□□□□□□□□□□□□□□□□□□□□□□□□□□□□□□□□□□□□□□□□□□□□□TT

AAGGG□□□□□□□□□□□□□□□□□□□□□□□□□□□□□□□□□□□□□□□□□□□□□□□□□□□□□□□□□□□□□□□□□□□□□□□□□□□□□□□□□□□□□□□□□□□□□□□□□□□□□□□□□□□□□□□□□□□□□□□□□□□□□□□□□□□□□□□□□□□□□□□□□□□□□□□□□□□□□□□□□□□□□□□□□□□□□□□□□□□□□□□□□□□□□□□□□□□□□□□□□□□□□□□□□□□□□□□□□□□□□□□□□□□□□□□□□□□□□□□□□□□□□□□□□□□□□□□□□□□□□□□□□□□□□□□□□□□□□□□□□□□□□□□□□□□□□□□□□□□□□□□□□□□□□□□□□□□□□□□□□□□□□□□□□□□□□□□□□□□□□□□□□□□□□□□□□□□□□□□□□□□T

AAGGG□□□□□□□□□□□□□□□□□□□□□□□□□□□□□□□□□□□□□□□□□□□□□□□□□□□□□□□□□□□□□□□□□□□□□□□□□□□□□□□□□□□□□□□□□□□□□□□□□□□□□□□□□□□□□□□□□□□□□□□□□□□□□□□□□□□□□□□□□□□□□□□□□□□□□□□□□□□□□□□□□□□□□□□□□□□□□□□□□□□□□□□□□□□□□□□□□□□□□□□□□□□□□□□□□□□□□□□□□□□□□□□□□□□□□□□□□□□□□□□□□□□□□□□□□□□□□□□□□□□□□□□□□□□□□□□□□□□□□□□□□□□□□□□□□□□□□□□□□□□□□□□□□□□□□□□□□□□□□□□□□□□□□□□□□□□□□□□□□□□□□□□□□□□□□□□□□□□□□□□□□□□□□□□□□□□□□□□□□□□□□□□□□□□□□□□□□□□□□□□□□□□□□□□□□□□□□□□□□□□□□□□□□□□□□□□□□□□□□□□□□□□□□□□□□□□□□□□□□□□□□□□□□□□□□□□□□□□□□□□□□□□□□□□□□□□□□□□□□□□T

AAGGG□□□□□□□□□□□□□□□□□□□□□□□□□□□□□□□□□□□□□□□□□□□□□□□□□□□□□□□□□□□□□□□□□□□□□□□□□□□□□□□□□□□□□□□□□□□□□□□□□□□□□□□□□□□□□□□□□□□□□□□□□□□□□□□□□□□□□□□□□□□□□□□□□□□□□□□□□□□□□□□□□□□□□□□□□□□□□□□□□□□□□□□□□□□□□□□□□□□□□□□□□□□□□□□□□□□□□□□□□□□□□□□□□□□□□□□□□□□□□□□□□□□□□□□□□□□□□□□□□□□□□□□□□□□□□□□□□□□□□□□□□□□□□□□□□□□□□□□□□□□□□□□□□□□□□□□□□□□□□□□□□□□□□□□□□□□□□□□□□□□□□□□□□□□□□□□□□□□□□□□□□□□□□□□□□□□□□□□□□□□□□□□□□□□□□□□□□□□□□□□□□□□□□□□□□□□□□□□□□□□□□□□□□□□□□□□□□□□□□□□□□□□□□□□□□□□□□□□□□□□□□□□□□□□□□□□□□□□□□□□□□□□□□□□□□□□□□□□□□□□□□□□□□□□□□□□□□□□□□□□□□□□□□□□□□□□□□□□□□□□□□□□□□□□□□□□□□□□□□□□□□□□□□□□□□□□□□□□□□□□□□□□□□□□□□□□□□□□□□□□□□□□□□□□□□□□□□□□□□□□□□□□□□□□□□□□□□□□□□□□□□□□□□□□□□□□□□□□□□□□□□□□□□□□□□□□□□□□□□□□□□□□□□□□□□□□□□□□□□□□□□□□□□□□□□□□□□□□□□□□□□□□□□□□□□□□□□□□□□□□□□□□□□□□□□□□□□□□□□□□□□□□□□□□□□□□□□□□□□□□□□□□□□□□□□□□□□□□□□□□□□□□□□□□□□□□□□□□□□□□□□□□□□□□□□□□□□□□□□□□□□□□□□□□□□□□□□□□□□□□□□□□□□□□□□□□□□□□□□□□□□□□□□□□□□□□□□□□□□□□□□□□□□□□□□□□□□□□□□□□□□□□□□□□□□□□□□□□□□□□□□□□□□□□□□□□□□□□□□□□□□□□□□□□□□□□□□□□□□□□□□□□□□□□□□□□□□□□□□□□□□□□□□□□□□□□□□□□□□□□□□□□□□□□□□□□□□□□□□□□□□□□□□□□□□□□□□□□□□□□□□□□□□□□□□□□□□□□□□□□□□□□□□□□□□□□□□□□□□□□□□□□□□□□□□□□□□□□□□□□□□□□□□□□□□□□□□□□□□□□□□□□□□□□□□□□□□□□□□□□□□□□□□□□□□□□□□□□□□□□□□□□□□□□□□□□□□□□□□□□□□□□□□□□□□□□□□□□□□□□□□□□□□□□□□□□□□□□□□□□□□□□□□□□□□□□□□□□□□□□□□□□□□□□□□□□□□□□□□□□□□□□□□□□□□□□□□□□□□□□□□□□□□□□□□□□□□□□□□□□□□□□□□□□□□□□□□□□□□□□□□□□□□□□□□□□□□□□□□□□□□□□□□□□□□□□□□□□□□□□□□□□□□□□□□□□□□□□□□□□□□□□□□□□□□□□□□□□□□□□□□□□□□□□□□□□□□□□□□□□□□□□□□□□□□□□□□□□□□□□□□□□□□□□□□□□□□□□□□□□□□□□□□□□□□□□□□□□□□□□□□□□□□□□□□□□□□□□□□□□□□□□□□□□□□□□□□□□□□□□□□□□□□□□□□□□□□□□□□□□□□□□□□□□□□□□□□□□□□□□□□□□□□□□□□□□□□□□□□□□□□□□□□□□□□□□□□□□□□□□□□□□□□□□□□□□□□□□□□□□□□□□□□□□□□□□□□□□□□□□□□□□□□□□□□□□□□□□□□□□□□□□□□□□□□□□□□□□□□□□□□□□□□□□□□□□□□□□□□□□□□□□□□□□□□□□□□□□□□□□□□□□□□□□□□□□□□□□□□□□□□□□□□□□□□□□□□□□□□□□□□□□□□□□□□□□□□□□□□□□□□□□□□□□□□□□□□□□□□□□□□□□□□□□□□□□□□□□□□□□□□□□□□□□□□□□□□□□□□□□□□□□□□□□□□□□□□□□□□□□□□□□□□□□□□□□□□□□□□□□□□□□□□□□□□□□□□□□□□□□□□□□□□□□□□□□□□□□□□□□□□□□□□□□□□□□□□□□□□□□□□□□□□□□□□□□□□□□□□□□□□□□□□□□□□□□□□□□□□□□□□□□□□□□□□□□□□□□□□□□□□□□□□□□□□□□□□□□□□□□□□□□□□□□□□□□□□□□□□□□□□□□□□□□□□□□□□□□□□□□□□□□□□□□□□□□□□□□□□□□□□□□□□□□□□□□□□□□□□□□□□□□□□□□□□□□□□□□□□□□□□□□□□□□□□□□□□□□□□□□□□□□□□□□□□□□□□□□□□□□□□□□□□□□□□□□□□□□□□□□□□□□□□□□□□□□□□□□□□□□□□□□□□□□□□□□□□□□□□□□□□□□□□□□□□□□□□□□□□□□□TT

AAGGG□□□□□□□□□□□□□□□□□□□□□□□□□□□□□□□□□□□□□□□□□□□□□□□□□□□□□□□□□□□□□□□□□□□□□□□□□□TT

AAGGG□□□□□□□□□□□□□□□□□□□□□□□□□□□□□□□□□□□□□□□□□□□□□□□□□□□□□□□□□□□□□□□□□□□□□□□□□□□□□□□□□□□□□□□□□□□□□□□□□□□□□□□□□□□□□□□TT

AAGGG□□□□□□□□□□□□□□□□□□□□□□□□□□□□□□□□□□□□□□□□□□□□□□□□□□□□□□□□□□□□□□□□□□□□□□□□□□□□□□□□□□□□□□□□□□□□□□□□□□□□□□□□□□□□□□□□□□□□□□□□□□□□□□□□□□□□□□□□□□□□□□□□□□□□□□□□□□□□□□□□□□□□□□□□□□□□□□□□□□□□□□□TT

AAGGG□□□□□□□□□□□□□□□□□□□□□□□□□□□□□□□□□□□□□□□□□□□□□□□□□□□□□□□□□□□□□□□□□□□□□□□□□□□□□□□□□□□□□□□□□□□□□□□□□□□□□□□□□□□□□□□□□□□□□□□□□□□□□□□□□□□□□□□□□□□□□□□□□□□□□□□□□□□□□□□□□□□□□□□□□□□□□□□□□□□□□□□□□□□□□□□□□□□□TT

AAGGG□□□□□□□□□□□□□□□□□□□□□□□□□□□□□□□□□□□□□□□□□□□□□□□□□□□□□□□□□□□□□□□□□□□□□□□□□□□□□□□□□□□□□□□□□□□□□□□□□□□□□□□□□□□□□□□□□□□□□□□□□□□□□□□□□□□□□□□□□□□□□□□□□□□□□□□□□□□□□□□□□□□□□□□□□□□□□□□□□□□□□□□□□□□□□□□□□□□□□□□□□□□□□□□□□□□□□□□□□□□□□□□□□□□□□□□□□□□□□□□□□□□□□□□□□□□□□□□□□□□□□□

**S20 Fig: Insertion of TVRs is cell line specific and arises from multiple events.** Examples of telomere sequences obtained from PacBio sequencing of STELA amplicons obtained from HCT116^ATRX-/o^ and HCA2^HPVE6E7;ATRX-/o^ cells at the XpYp, 17p and 7q chromosome ends prior to crisis (Parental) and a single clone of each exhibiting characteristics of ALT post crisis, denoted as ALT from HCA2^HPVE6E7;ATRX-/o^ fibroblasts and ALT-L (ALT-like) from HCT116^ATRX-/o^ and ALT positive U2OS cells. Telomere and 6 nt variant repeat sequences are coded as follows: □ TTAGGG; □ TCAGGG; □ TTCGGG; □ GTAGGG; □ TGAGGG; □ TTGGGG; □ TAAGGG; □ CTAGGG; □ TTTGGG; □ AGAGGG; □ TVRs ≶ 6 nt.
