# Supplementary material for: ATRX modulates the escape from a telomere crisis
Source: PLoS Genet. 2022 Nov 9;18(11):e1010485. doi: 10.1371/journal.pgen.1010485 (PMC9678338; doi:10.1371/journal.pgen.1010485)
Supplement: S21 Fig — A) Structural variant counts for ALT-surviving, ALT-died and telomerase-positive clones with the P value as determined by a Mann-Whitney test stated above. Statistical difference highlighted in red (P value < 0.05). Clones and timepoints at which telomerase was active are highlighted in orange. B) Complex rearrangements on chromosomes 3 and 13 in ALT-like clone 147. (DOCX) [file pgen.1010485.s021.docx]

**
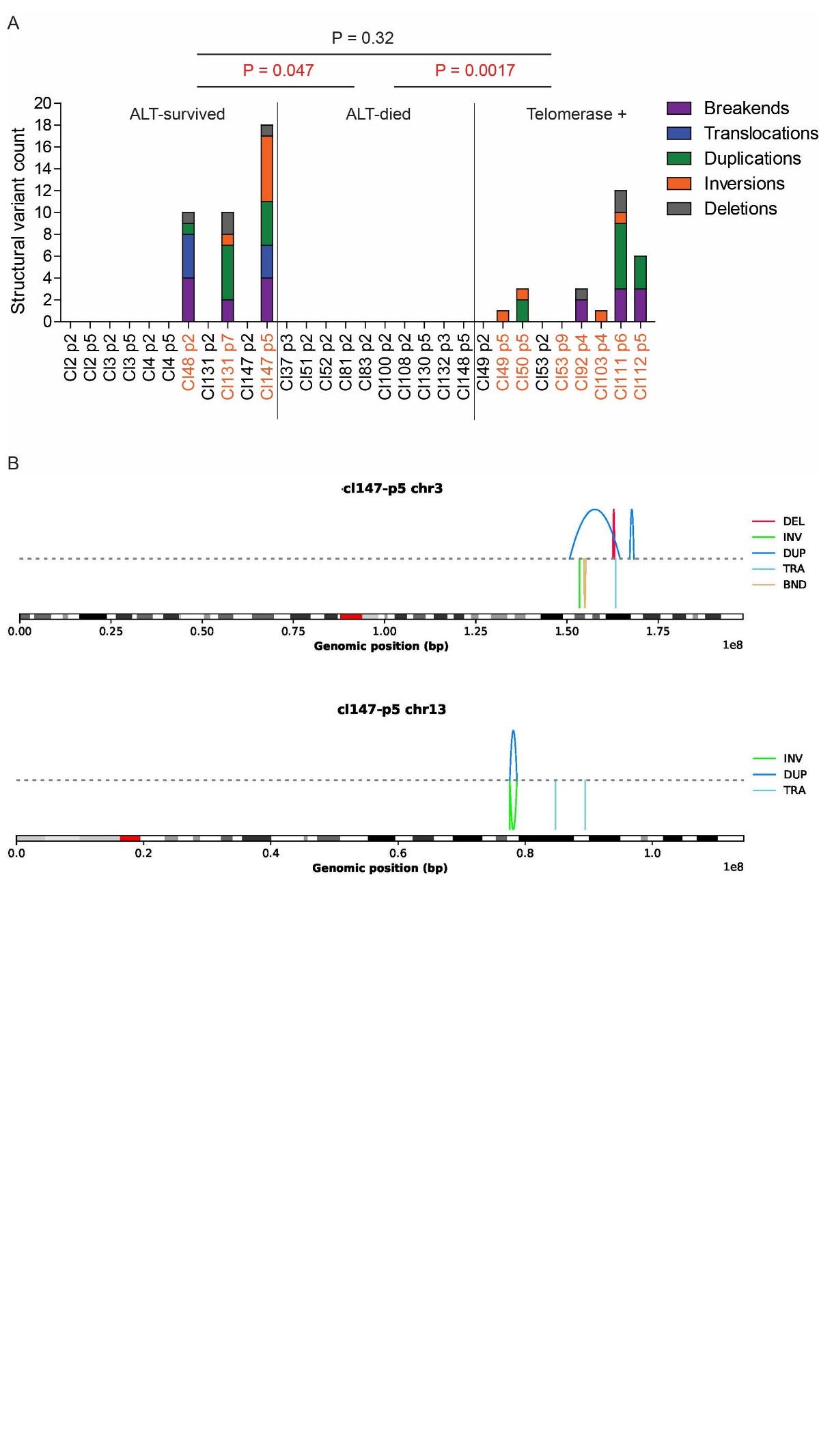
**

**S21 Fig: Increased rate of structural variants in telomerase escapees but not in cells exhibiting ALT-like characteristics.** A) Structural variant counts for ALT-surviving, ALT-died and telomerase-positive clones with the P value as determined by a Mann-Whitney test stated above. Statistical difference highlighted in red (P value < 0.05). Clones and timepoints at which telomerase was active are highlighted in orange. B) Complex rearrangements on chromosomes 3 and 13 in ALT-like clone 147.
