# Supplementary material for: ATRX modulates the escape from a telomere crisis
Source: PLoS Genet. 2022 Nov 9;18(11):e1010485. doi: 10.1371/journal.pgen.1010485 (PMC9678338; doi:10.1371/journal.pgen.1010485)
Supplement: S1 Table — (DOCX) [file pgen.1010485.s022.docx]

**S1 Table. Summarising data from HCT116 clones**

| Total clones | 149 |
| --- | --- |
| Survived/died | 33/116 |
| C-circle +/C-circle - | 25/124 |
| Telomere elongation/C-circle + | 6/25 |
| ALT-like escapee/Telomerase escapee | 4/29 |
