# Supplementary material for: ATRX modulates the escape from a telomere crisis
Source: PLoS Genet. 2022 Nov 9;18(11):e1010485. doi: 10.1371/journal.pgen.1010485 (PMC9678338; doi:10.1371/journal.pgen.1010485)
Supplement: S1 Methods — (PDF) [file pgen.1010485.s023.pdf]

ATRX modulates the escape from a telomere crisis

Helene E. B. Geiller[1], Adam Harvey[2], Rhiannon E. Jones[1], Julia W. Grimstead[1], Kez Cleal[1], Eric Hendrickson[2*] and Duncan M. Baird[1*].

[1]Division of Cancer and Genetics, School of Medicine, Cardiff University, Heath Park, Cardiff, CF14 4XN, United Kingdom

[2]Department of Biochemistry, Molecular Biology, and Biophysics, University of Minnesota Medical School, Minneapolis, MN 55455, USA

*Co-senior authors.

Supplementary Methods

Identifying sequencing primers

Raw sequencing reads were first filtered, keeping reads with a sub-telomere primer at one end and a telorette primer at the other in complementary orientations: *i.e.* Forward-Reverse, or Reverse-Forward. This was achieved by comparing the extremities of each read to primer or telorette sequences. Edlib was used to align the read and primer sequences, and an edit distance < 7 served as a threshold to determine the presence or absence of a primer [1].

Labelling of sequences and telmers

Sequences were labelled using a Hidden Markov Model (HMM) [2] to identify the telomere array, sub-telomere sequences, and any telomeric interstitial or end insertions. Initially, input sequences were broken into overlapping kmers of length 6 bp. Each kmer received a label of either ‘0’ which denoted a background sequence or non-telomeric sequence, ‘1’ which denoted a forward-strand telomere sequence (CCCTAA-like), or ‘2’ which denoted a reverse-strand telomere sequence (TTAGGG-like). This was achieved by generating all rotations of the canonical telomere repeat motif, for example the first

two rotations of TTAGGG correspond to GTTAGG and GGTTAG, in total generating two sets of 6 sequence rotations for forward and reverse telomere motifs. We refer to each of these disjoint sets of telomere sequence rotations as telmers which represent the forward and reverse canonical telomere repeat motif.

Next, each kmer was compared against telmer sets using Edlib to align sequences, and using an edit distance of < 2 as a threshold to determine a match. If a kmer was matched with a forward telmer then a label of 1 was given to the kmer, a label of 2 denoted a match with a reverse telmer, whilst 0 denoted no match.

The series of observations were then segmented by a HMM using the Pomegranate library [3]. The model comprised of three discrete distributions corresponding to the three hidden states of S_1 background sequence, S_2 forward telomere sequence, and S_3 reverse telomere sequence. The emission probabilities for each of the three states were as follows:

Background $S_1 = \{0: 0.8, 1: 0.1, 2: 0.1\}$

Forward telomere $S_2 = \{0: 0.1, 1: 0.8, 2: 0.1\}$

Reverse telomere $S_3 = \{0: 0.1, 1: 0.1, 2: 0.8\}$

Transition probabilities were manually set as follows:

$Start \rightarrow S_1 = 0.6$

$Start \rightarrow S_2 = 0.3$

$Start \rightarrow S_3 = 0.3$

$S_1 \rightarrow S_1 = 0.95$

$S_1 \rightarrow S_2 = 1e^{-9}$

$S_1 \rightarrow S_3 = 1e^{-9}$

$S_2 \rightarrow S_2 = 0.8$

$S_2 \rightarrow S_1 = 1e^{-9}$

$S_2 \rightarrow S_3 = 1e^{-9}$

$S_3 \rightarrow S_3 = 0.8$

$S_3 \rightarrow S_2 = 1e^{-9}$

$S_3 \rightarrow S_3 = 1e^{-9}$

The model was then normalised by calling the “bake” method. Sequences were then segmented and classified. Telomere array was identified by a segment label of 1 or 2. Sub-telomere sequences were identified as segments of background sequence extending from the PCR primer to the start of the telomere array. Interstitial insertions were identified as blocks of background sequence found within the telomere array, and end insertions were identified as blocks of background sequence positioned adjacent to the telorette sequence at the end of the telomere array.

Cleaning of sequence data

Further filtering steps were performed that aimed to remove potential sequencing or PCR artifacts and other anomalous sequences from the raw sequencing data. Together these aimed to 1) remove unexpected non-sub-telomeric sequences that were occasionally amplified due to low homology with sequencing primers; 2) remove STELA-like products that showed evidence of primer swapping, occurring when the sequenced primer did not match the expected sub-telomere sequence; 3) remove STELA-like products that had no discernible sub-telomere sequence; 4) remove apparent concatemers of STELA products deemed to be an artifact of PCR or PacBio sequencing.

Firstly, the expected sub-telomere sequences associated with each of the PCR primers were extracted from the GRCh38 human reference genome, corresponding to the reference sequence from the

primer site to the start of the telomere repeat array. For each input sequence, the sub-telomere segment was then aligned to the expected reference-derived sub-telomere sequence. Edlib was used for alignment with arguments mode = 'HW'. To meet the filtering goals 1, 2 and 3 listed above, sequences were discarded if any of the following conditions were met: no alignment with any reference-sub-telomeres; the edit distance of the alignment corresponded to > 0.1 x input sequence length; the total sub-telomere segment length was < 30 bp; the PCR primer did not match the expected sub-telomere class. To identify concatemers, interstitial insertions were mapped to the GRCh38 reference genome using bwa mem with options '-x PacBio -a' to generate all mappings. An optimal set of alignments was then chosen using dodi align [4] (found online at: https://github.com/kcleal/dodi), supplying the list of target sub-telomere loci in ".bed" format using the --include option. Dodi align outputs a spanning set of alignments consisting of primary and supplementary alignments, but filters out secondary alignments and nested alignments. Supplying a list of target regions with the --include option has the result of favouring alignments that fall within those target regions, and can thus be used to identify a spanning set of alignments that preferentially includes target regions of the genome. If an alignment was identified that overlapped one of the target sub-telomere regions then the sequence was regarded as a concatemer and discarded.

Telomere variant repeat abundance

Each of the target telomere variant repeats was converted into a corresponding telmer set, as described. Of these, 15 were 6 bp in length with a single 7-mer TTAAGGG, giving a set of 97 rotation sequences that mapped to 16 telmers. To quantify the abundance of telomere variant repeats, the whole telomere repeat array including any insertions was analysed, from the start of the telomere array to the beginning of the telorette sequence. For the 6 bp telmers, the telomere array sequence was decomposed into 6 bp kmers. If any kmer exactly matched a rotation sequence the corresponding telmer count was incremented. The same procedure was repeated for the 7-mer telomere variant

repeat, noting that the counts for the 7-mer are therefore not independent of the TAAGGG telmer, as every TTAAGGG will also be counted as TAAGGG, but not *vice versa*.

Allele separation by telomere variant repeat content

Different telomere alleles were first identified by manual inspection of reads. To generate a prototype signature for each of the alleles, a random selection of reads was drawn from the bulk data, corresponding to each of the target alleles, separating a minimum of 20 example reads for each allele. For each sequence, only the first 100 bp of the telomere repeat array was analysed further. Counts for the 16 telmer classes were then determined, as described. Additionally, the counts of any 6 bp kmers that did not match a telmer were also recorded. Thus, the first 100 bp of the telomere repeat array was converted into a count matrix with 17 columns. For each collection of input sequences, the mean across count matrices was taken, generating a single count matrix or signature for each allele. Next, each read from the bulk data was processed in the same way, deconstructing the first 100 bp of the telomere array into a count matrix. To classify reads into different alleles, the cosine similarity between the derived count matrix and each allele signature was calculated, and a threshold of 0.10 was used to identify a match.

Programming and statistics

Scripts were written using Python3 and statistical testing was carried out using the Scipy package [5].

1 Sosic, M. & Sikic, M. Edlib: a C/C ++ library for fast, exact sequence alignment using edit distance. *Bioinformatics* **33**, 1394-1395, doi:10.1093/bioinformatics/btw753 (2017).

2 Eddy, S. R. What is a hidden Markov model? *Nat Biotechnol* **22**, 1315-1316, doi:10.1038/nbt1004-131510.1038/nbt1004-1315. (2004).

3 Schreiber, J. *Pomegranate: fast and flexible probabilistic modeling in python*. Vol. 18 (JMLR.org, 2017).

4 Cleal, K., Jones, R. E., Grimstead, J. W., Hendrickson, E. A. & Baird, D. M. Chromothripsis during telomere crisis is independent of NHEJ, and consistent with a replicative origin. *Genome Res* **29**, 737-749, doi:10.1101/gr.240705.118 (2019).
5 Jones E, O. E., Peterson P. SciPy: Open Source Scientific Tools for Python. (2001).
